# Tuberculosis Treatment in HIV Infected Ugandans with CD4 Counts >350 Cells/mm^3^ Reduces Immune Activation with No Effect on HIV Load or CD4 Count

**DOI:** 10.1371/journal.pone.0009138

**Published:** 2010-02-22

**Authors:** C. Scott Mahan, Maria Walusimbi, Denise F. Johnson, Christina Lancioni, Edwin Charlebois, Joyce Baseke, Keith A. Chervenak, Roy D. Mugerwa, Diane V. Havlir, Harriet Mayanja-Kizza, Christopher C. Whalen, W. Henry Boom, for the Uganda-Case Western Research Collaboration

**Affiliations:** 1 Tuberculosis Research Unit, Case Western Reserve University School of Medicine, Cleveland, Ohio, United States of America; 2 Division of Infectious Diseases, Department of Medicine, MetroHealth Medical Center, Cleveland, Ohio, United States of America; 3 Makerere University School of Medicine, Mulago Hospital, Uganda-CWRU Research Collaboration, Kampala, Uganda; 4 HIV/AIDS Division, San Francisco General Hospital, University of California, San Francisco, San Francisco, California, United States of America; 5 Department of Epidemiology and Biostatistics, College of Public Health, University of Georgia, Athens, Georgia, United States of America; 6 University Hospitals-Case Medical Center, Cleveland, Ohio, United States of America; Faculty of Medicine Tel Aviv University, Israel

## Abstract

**Background:**

Both HIV and TB cause a state of heightened immune activation. Immune activation in HIV is associated with progression to AIDS. Prior studies, focusing on persons with advanced HIV, have shown no decline in markers of cellular activation in response to TB therapy alone.

**Methodology:**

This prospective cohort study, composed of participants within a larger phase 3 open-label randomized controlled clinical trial, measured the impact of TB treatment on immune activation in persons with non-advanced HIV infection (CD4>350 cells/mm^3^) and pulmonary TB. HIV load, CD4 count, and markers of immune activation (CD38 and HLA-DR on CD4 and CD8 T cells) were measured prior to starting, during, and for 6 months after completion of standard 6 month anti-tuberculosis (TB) therapy in 38 HIV infected Ugandans with smear and culture confirmed pulmonary TB.

**Results:**

Expression of CD38, and co-expression of CD38 and HLA-DR, on CD8 cells declined significantly within 3 months of starting standard TB therapy in the absence of anti-retroviral therapy, and remained suppressed for 6 months after completion of therapy. In contrast, HIV load and CD4 count remained unchanged throughout the study period.

**Conclusion:**

TB therapy leads to measurable decreases in immune activation in persons with HIV/TB co-infection and CD4 counts >350 cells/mm^3^.

## Introduction

Cellular markers of immune activation, CD38 and HLA-DR on CD4 and CD8 T lymphocytes, are elevated in HIV infected individuals. Opportunistic infections such as tuberculosis (TB) lead to further increases in immune activation and stimulate HIV viral replication [1]. Sulkowski et al. showed that successful treatment of opportunistic infections, excluding TB, led to declines in HIV load and decreased immune activation [2]. One would predict that treatment of TB would also lead to declines in HIV load and increases in CD4 count, but studies addressing this question have been contradictory. Studies within the United States and Europe have shown a decrease in HIV load in response to TB therapy [3], [4]. In contrast, studies from sub-Saharan Africa have failed to see declines in HIV load after several months of TB therapy [5]–[7]. It is a similar story for CD4 counts. In HIV negative individuals active TB disease induces CD4 lymphopenia which is responsive to TB therapy [8]. However, in persons with HIV/TB co-infection, treatment of TB has not impacted CD4 counts [5], [6].

Immune activation has been implicated as a critical driver of HIV disease progression. Expression of CD38 on CD8 T cells has been shown to be a strong predictor of HIV progression and death [9]. Despite multiple studies demonstrating increased CD38 and/or HLA-DR expression on CD8 T cells in persons with or without HIV co-infection during active TB there are few studies showing declines in these cellular markers of immune activation in response to TB treatment. Our earlier studies have shown that TB exerts its greatest effect on survival in persons with early stage HIV [10]. We therefore hypothesized that TB therapy alone in TB patients with less advanced HIV would lead to measurable declines in immune activation and corresponding declines in HIV load. To test this hypothesis we prospectively followed a cohort of 38 HIV positive persons with baseline CD4 counts >350 cells/mm^3^ in Uganda who had smear and culture confirmed pulmonary TB and was placed on standard anti-TB therapy.

## Materials and Methods

We present a cohort study of participants in a phase 3 open-labeled randomized clinical trial measuring the impact of early HAART combined with standard TB therapy versus TB therapy alone in persons with pulmonary TB and HIV infection with high CD4 counts. Subjects, between the ages of 18 and 60, were recruited from the Uganda-Case Western Reserve University Research Collaboration within the Mulago Hospital Complex in Kampala, Uganda. All subjects gave written informed consent. This sub-study focused on the anti-retroviral therapy naïve HIV positive persons with smear positive pulmonary TB and CD4 counts >350 cells/mm^3^ who were started on a standard regimen of directly observed TB therapy (two months of isoniazid, rifampin, pyrazinamide, and ethambutol, followed by four months of isoniazid and rifampin). Mid-way through study enrollment daily cotrimoxazole was added in response to new international guidelines. At the 3, 6, 9, and 12 month study visits 84%, 92%, 84%, and 87% of the participants were taking daily cotrimoxazole. The study protocol was approved by the institutional review boards of Case Western Reserve University (CWRU), University of California at San Francisco, the Joint Clinical Research Center in Kampala, Uganda and the Ugandan National Council for Science and Technology (Figures S1–S3).

Enrollment for the larger clinical trial began in October 2004. The protocol for this trial, supporting CONSORT checklist and flow diagram of enrollment are available as supporting information (Checklist S1, Protocol S1, Figure S4). In this substudy, data was accrued between March 2006 and February 2009. Participants gave informed consent and had blood obtained for immune analysis at baseline, 3, 6, 9, and 12 months after enrollment. All persons with flow data at baseline, 3 and/or 6 months, and 9 and/or 12 months were included in the analysis (n = 38). All patients completed TB treatment by month 6. Immunologic studies were performed at the Joint Clinical Research Center in Kampala, Uganda.

Plasma HIV-RNA copy levels (HIV load) were measured using Amplicor quantitative restriction transcriptase-polymerase chain reaction assay (Roche Amplicor 1.5) according to manufacturer instructions. The lower limit of detection of the assay was 400 copies/mm^3^.

Whole blood was collected in sodium heparin tubes and 200 ul was aliquotted to each of the 12×75 mm test tubes. For flow cytometry the following antibodies were used: anti-CD4 allophycocyananin (APC), anti-CD8 APC, anti-HLA-DR phycoerythrin (PE), and anti-CD38 phycoerythrin Cy5 (PE Cy5). Mouse monoclonal isotypic controls conjugated with PE, PE Cy5, and APC were used to determine non-specific binding and to set gating boundaries. All antibodies were obtained from BD Pharmingen (San Diego, California).

**Figure 1:**
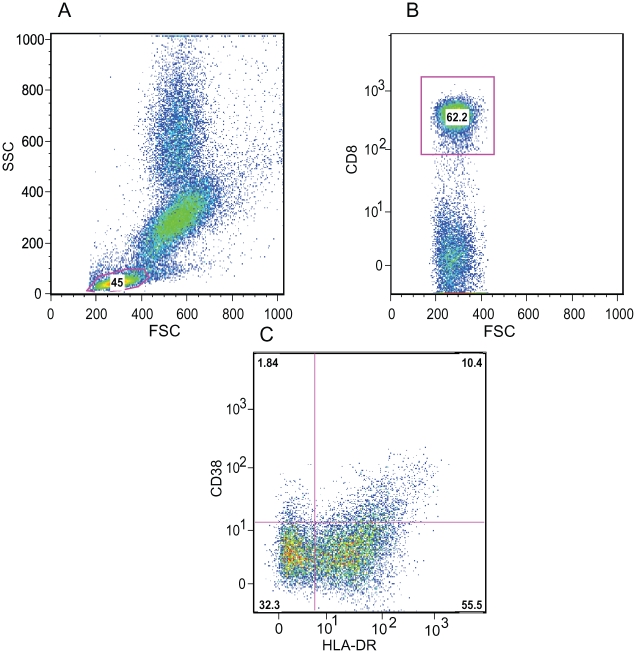
Representative Flow cytometric approach to define immune activation of CD8 T cells. A. Gating on lymphocytes based on forward and side scatter properties. B. Gating on CD8 T cells. C. Expression of CD38 and HLA-DR on CD8 T cells.

Using 4 color flow cytometry (Becton Dickinson FACSCalibur) 25–50,000 cells were analyzed for each condition. Initial gating was on the lymphocytes based on forward and side scatter. CD4 or CD8 T cell populations were then determined. T cells were then evaluated for the percentage of cells expressing HLA-DR and CD38 (Figure 1).

Non-parametric rank tests were performed to compare median changes in immune activation from baseline to months 3, 6, 9, and 12; and to compare levels at 6 months with 9 and 12 months (the period after TB therapy was completed). Data were stratified by cotrimoxazole use to determine its effect on immune activation at each time point. All analyses were performed using Statistical Analysis Software (SAS) Version 9.1 (SAS Institute, Cary, NC).

## Results

Of the 38 HIV infected persons with sputum smear and culture confirmed pulmonary TB the mean age was 32 years (range 19–54) and 24 were males (63%) and 14 female. The median baseline HIV load was 4.4 log_10_ copies/ml (range 2.0–5.9 log_10_ copies/mL) and the median CD4 count was 610 cells/mm^3^ (range 374–1368). 36 of 38 persons were sputum culture negative at 2 months, and all 38 were culture negative by 4 months while on TB treatment. Among these 38 persons there was only one death which occurred 10 months after completion of TB therapy.

**Figure 2:**
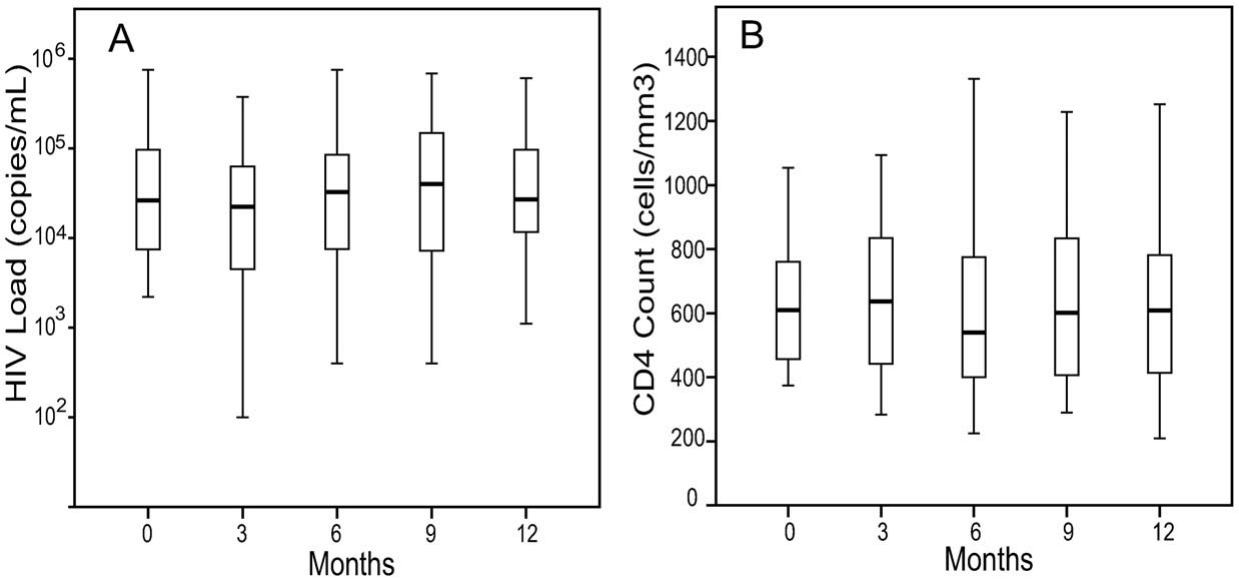
Change in HIV Load and CD4 Counts in response to TB therapy. A. HIV log_10_ RNA viral loads on standard TB therapy at baseline, 3, 6, 9, and 12 months. B. CD4 counts at baseline, 3, 6, 9, and 12 months (n = 38 for all time points). The boxes indicate the interquartile ranges, the horizontal lines transecting the boxes indicate the medians, and the whiskers indicate the highest and lowest values. All values with P>.05.

There was no significant change in median HIV log_10_ load as compared to baseline at 3 (4.3 log_10_ copies/mL), 6 (4.5 log_10_ copies/mL), 9 months (4.6 log_10_ copies/mL), or 12 months (4.4 log_10_ copies/mL) (Figure 2A). Additionally there was no significant change in median CD4 count from a baseline value of 610 cells/mm^3^, to 637 cells/mm^3^ at 3 months, 540 cells/mm^3^ at 6 months, 602 cells/mm^3^ at 9 months, and 608 cells/mm^3^ at 12 months. (Figure 2B).

**Figure 3:**
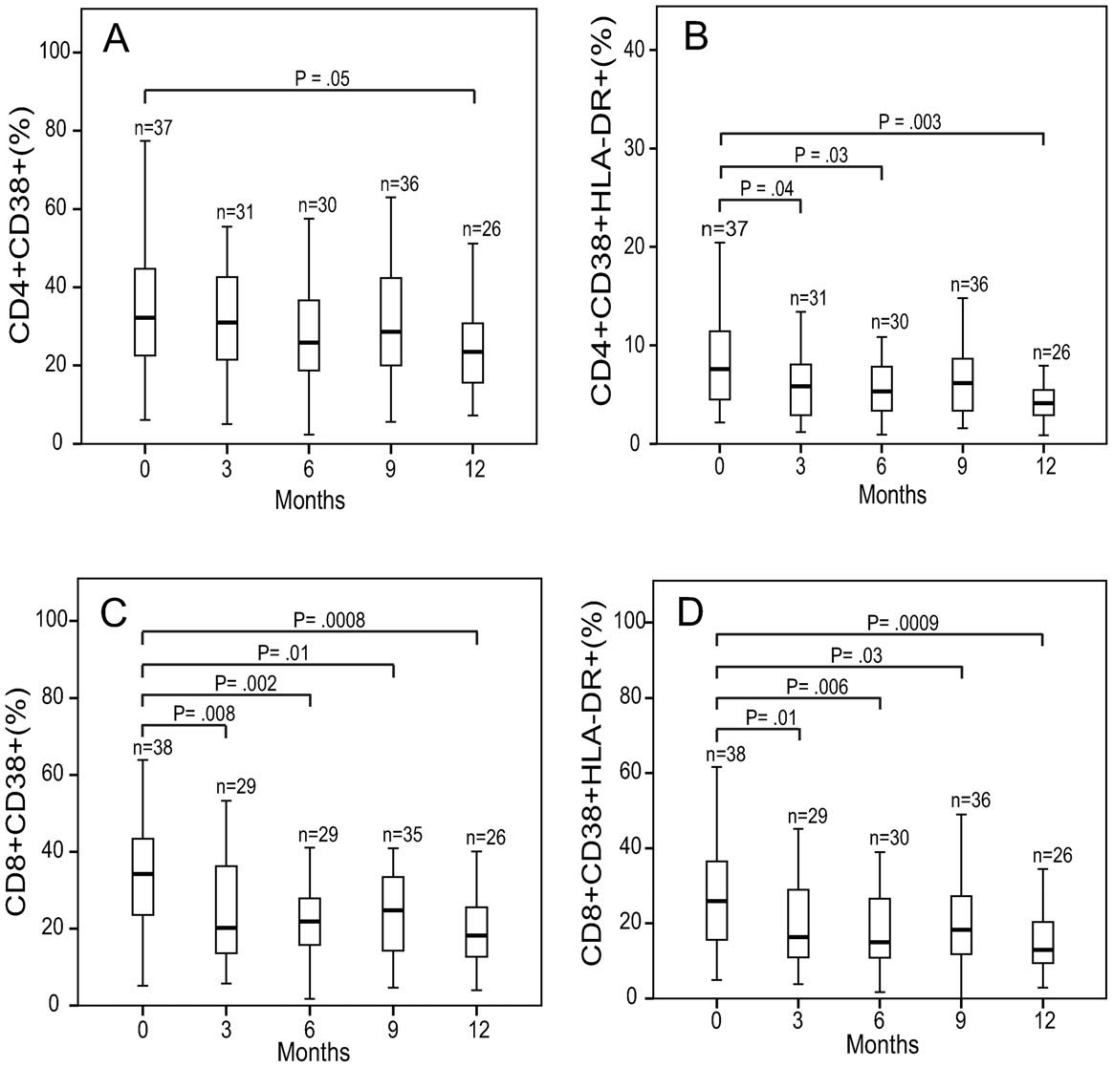
Change in immune activation in response to TB therapy. Percentage expression of CD38 (A,C), and CD38/HLA-DR (B,D) on CD4 and CD8 T cells at baseline, 3, 6, 9, and 12 months. The boxes indicate the interquartile ranges, the horizontal lines transecting the boxes indicate the medians, and the whiskers indicate the highest and lowest values. Significant P values (<.05) are indicated.

The change in median percentage of CD4 cells expressing CD38 was unchanged from baseline (32%), to 3 (31%), 6 (26%), and 9 months (29%), but measured a significant decline at 12 months (24%) (Figure 3A). The percentage of CD4 cells that were double positive, expressing both HLA-DR and CD38, declined from a baseline value of 8% to 6% (P<.05) at 3 months, 5% (P<.05) at 6 months, at 9 months the change from baseline was no longer significant (6%) (P>.05), but regained significance at 12 months (4%) (P<.01) (Figure 3B).

The median percentage of CD8 cells expressing CD38 declined from 34% at baseline to 20% (P<.01) at 3 months, 22% (P<.01) at 6 months, to 25% (P<.05) at 9 months, to 18% at 12 months (P<.001) (Figure 3C). The percentage of CD8 cells that were double positive, expressing both HLA-DR and CD38, declined from 26% at baseline to 16% (P<.01) at 3 months, 15% (P<.01) at 6 months, to 18% (P<.05) at 9 months, and to 13% at 12 months (P<.001) (Figure 3D). HLA-DR expression alone on CD4 and CD8 cells was not significantly changed from baseline. Thus, the expression of CD38 and the co-expression of CD38/HLA-DR on CD8 cells were strongly affected by TB therapy while decreased expression of these markers on CD4 cells was less pronounced.

The primary analysis used for figures 2 and 3 was Wilcoxon rank test for non-parametric values. Data sets were also analyzed by Sign test which confirmed all values of statistical significance. Additionally, stratified analysis showed no significant impact of cotrimoxazole use on immune activation of CD8 T cells. When accounting for cotrimoxazole use the combined expression of CD38/HLA-DR on CD4 T cells was no longer significant at 6 months but remained so at 12 months.

## Discussion

In this prospective cohort study of persons with HIV (CD4 counts>350 cells/mm^3^) and pulmonary TB we measured a significant decline in CD8 T cell immune activation that persisted for at least 6 months after completion of TB therapy. We did not observe decreased HIV load or increased CD4 counts in response to TB therapy.

Both HIV and MTB infection cause immune activation. How HIV infection leads to chronic immune activation is poorly understood. Depletion of gut mucosal lymphocytes and transudation of LPS into the systemic circulation has been postulated by some. Alternatively, HIV and its viral gene products can directly activate the immune system [11]. In active TB, macrophages release pro-inflammatory cytokines (IL-1, IL-6, TNF-α) in response to mycobacterial proteins and glycolipids, resulting in CD4 and CD8 T lymphocyte recruitment and activation at the site of infection and systemically [12]. CD38 and HLA-DR expression on CD8 cells, markers of immune activation, are higher in HIV/TB co-infection than in persons with either HIV infection or TB alone [13].

In HIV/TB co-infection, TB leads to increased HIV viral replication both in the lungs and systemically. TNF-α produced in response to mycobacterial proteins induces HIV viral replication through nuclear factor kappa β (NFK-β) binding to the 5′ end of the long terminal repeat of HIV [12]. Immune activation and inflammatory cytokines induced by opportunistic infections such as MTB can drive HIV viral replication. Thus one would predict that TB treatment should lead to a decrease in immune activation and a corresponding decline in HIV load.

Our finding that reduced immune activation did not result in a reduction in HIV load suggests that there may not be a linear relationship between immune activation and viral replication in HIV/TB. The anti-inflammatory/-infective properties of cotrimoxazole and rifampin could contribute to decreasing immune activation. However, when data was stratified for cotrimoxazole use there was no impact on CD38 expression. The lack of a rebound six months after completing TB therapy argues against a role for rifampin. The possible disconnect between immune activation and HIV load is further supported by our observation that up to 24% of HIV/TB co-infected Ugandans have low baseline HIV viral loads that are independent of CD4 counts and severity of TB [14]. While studies performed in the United States and Great Britain show that TB treatment decreases HIV load [3], [4], our study is consistent with similar work from sub-Saharan Africa that found no change [6], [7].

Our most striking finding was a clear decline in immune activation seen as early as 3 months into treatment. Percentage expression of both CD38 alone and co-expression of CD38/HLA-DR on CD8 T cells was significantly decreased after 3 and 6 months of standard TB therapy and remained decreased for at least 6 months after completion of therapy. Additionally, there was a trend towards decreased expression of CD38 and HLA-DR on CD4 T cells. These findings are at odds with Morris et al. who failed to see a decline in immune activation in their sub-Saharan HIV/TB co-infected cohort [6]. Although both cohorts were in sub-Saharan Africa, our cohort had much higher CD4 counts at baseline (mean of 642 cells/mm^3^) compared to the Morris cohort (mean CD4 count of 184 cells/mm^3^). It is quite possible that normal regulatory patterns that induce macrophages and T cells to produce pro-inflammatory cytokines are disrupted in advanced HIV disease and are less likely to normalize in response to TB therapy [15]. In contrast, in less advanced HIV infection TB therapy may be more likely to re-establish normal cytokine responses leading to a corresponding decline in immune activation. Epidemiologic studies have shown that treatment of TB has a greater impact on survival in HIV infected persons with higher CD4 counts (>200 cells/mm^3^), and little impact on those with more advanced HIV disease [10].

The failure of CD4 counts to increase in response to TB therapy is also intriguing. TB in non-HIV infected persons induces lymphopenia that returns to normal within one month of treatment [8]. This response may be offset by the increased T cell apoptosis in HIV/TB [12]. Our findings are in-line with others who have shown no increase in CD4 counts with TB therapy in HIV infected individuals [5], [6]. Conversely one might argue that the relatively stable CD4 counts over 1 year, as opposed to a steady decline, are indirect evidence for further immunologic benefit.

Limitations of our study include the lack of control group and limited number of patients. The ideal control group would be similar persons with HIV/TB co-infection that are followed and do not receive TB treatment. For obvious reasons this study will not be performed. Alternatively, one might compare our group to those with HIV infection alone, but these persons even if having similar CD4 and HIV loads at baseline are unlikely to have similar baseline levels of immune activation [16]. Despite following only 38 persons longitudinally we were able to find a measurable decline in immune activation. One cannot exclude the possibility that our study lacked the power to detect more subtle, but still significant changes in HIV load and CD4 counts.

In conclusion, we found that TB therapy in HIV/TB co-infected persons with CD4 counts >350 cells/mm^3^ leads to significant declines in immune activation of CD8 T cells without a measurable impact on HIV load or CD4 counts. Our findings suggest that declines in immune activation rather than changes in HIV load or CD4 count may explain prior epidemiologic observations that TB treatment leads to survival benefits in HIV/TB co-infected patients with less advanced HIV disease.

## Supporting Information

Protocol S1 Trial Protocol for the parent study.
(2.10 MB DOC)

Checklist S1 CONSORT Checklist
(0.19 MB DOC)

Figure S1 IRB approval from the Ugandan Council of Science and Technology.
(0.03 MB PDF)

Figure S2 IRB Approval from the Joint Clinical Research Center in Kampala, Uganda.
(0.04 MB PDF)

Figure S3 IRB approval from University Hospitals-Case Medical Center.
(0.22 MB PDF)

Figure S4 Enrollment Flow diagram.
(0.03 MB DOC)
