# Supplementary material for: Tuberculosis Treatment in HIV Infected Ugandans with CD4 Counts >350 Cells/mm3 Reduces Immune Activation with No Effect on HIV Load or CD4 Count
Source: PLoS One. 2010 Feb 22;5(2):e9138. doi: 10.1371/journal.pone.0009138 (PMC2825253; doi:10.1371/journal.pone.0009138)
Supplement: Protocol S1 — Trial Protocol for the parent study. (2.10 MB DOC) [file pone.0009138.s001.doc]

Delaying HIV disease Progression with Punctuated Antiretroviral Therapy in Patients with Tuberculosis in Uganda

# Principal Investigators

Christopher Whalen, M.D., M.S.

Professor of Epidemiology and Biostatistics

Case Western Reserve University

School of Medicine

Tuberculosis Research Unit

10900 Euclid Avenue

Cleveland, Ohio 44106-4984 U.S.A.

PH: (216) 368-4192

Fax: (216) 368-3970

Email: ccw@po.cwru.edu

Diane Havlir, M.D.

Professor of Medicine

University of California, San Francisco

School of Medicine

San Francisco General Hospital

995 Portrero Avenue

Building 80, Ward 84

San Francisco, CA 94110

PH: (415) 476-4082 ext. 400

Fax: (415) 502-2992

Email: [dhavlir@php.ucsf.edu](mailto:dhavlir@php.ucsf.edu)

Peter Mugyenyi, M.D.

Director

Joint Clinical Research Center

Butikiro House, Plot No. 893 Ring Road

Kampala, Uganda

Ph: +256 41 270283

Fax: +256-41-342632

Email: pmugyenyi@jcrc.co.ug

Roy D. Mugerwa, M.B.Ch.B.,M.Med

Professor of Medicine

Makerere Medical School - Mulago

Hospital and Complex

Uganda-CWRU Research Collaboration

P.O. Box 663

Kampala, Uganda

Ph/Fax: +256 (41) 540-718

E-mail: profrdm@imul.com

**Co-Investigators**

W. Henry Boom, M.D.

Director, Tuberculosis Research Unit

Professor of Medicine

Case Western Reserve University

School of Medicine

Tuberculosis Research Unit

10900 Euclid Avenue

Cleveland, Ohio 44106-4984 U.S.A.

Ph: +1 (216) 368-1949

Fax: +1 (216) 368-0105

E-mail: [whb3@po.cwru.edu](mailto:whb3@po.cwru.edu)

Harriet Mayanja-Kizza, M.B.Ch.B,M.Med., M.S.

Department of Medicine

Makerere University

Mulago Hospital and Complex

Uganda-CWRU Research Collaboration

P.O. Box 663

Kampala, Uganda

Ph: +256 (41) 534-262

Fax: +256 (41) 345-643

E-mail: [cwru@mucwru.or.ug](mailto:cwru@mucwru.or.ug)

Edwin Charlebois, Ph.D.

Assistant Professor of Medicine,

Division of Infectious Diseases

EPI-Center

University of California, San Francisco

San Francisco General Hospital

1001 Potrero Ave. UCSF 1372

Building 100, Room 303

San Francisco, CA. 94110

Ph: (415) 206-4977

Fax: (415) 206-4978

Email: Edwin.charlebois@ucsf.edu

Alphonse Okwera, M.B.Ch.B., M.Sc.

Director, National TB Treatment Center

National TB and Leprosy Programme

Mulago Hospital and Complex

Uganda-CWRU Research Collaboration

P.O. Box 663

Kampala, Uganda

Ph: +256 (41) 534-262

Fax: +256 (41) 345-643

Email: cwru@imul.com

Cissy Mutuluuza Kityo, M.B.Ch.B., M.Sc.,

Joint Clinical Research Center

Butikiro House, Plot No. 893 Ring Road

Kampala, Uganda

Ph: +256 41 270283

Fax: +256-41-342632

Email: ckityo@jcrc.co.ug

Table of Contents

[**Principal Investigators 2**](#__RefHeading___Toc96506743)

[APPENDICES 8](#__RefHeading___Toc96506744)

[Participating Sites 9](#__RefHeading___Toc96506745)

[Protocol Synopsis 10](#__RefHeading___Toc96506746)

[1.0 INTRODUCTION 12](#__RefHeading___Toc96506747)

[1.1 RATIONALE 12](#__RefHeading___Toc96506748)

[1.1.1 Evidence for Model of HIV/TB Co-pathogenesis 12](#__RefHeading___Toc96506749)

[1.1.2 Targeting HIV Replication with Antiretroviral Treatment 14](#__RefHeading___Toc96506750)

[1.1.3 Treatment of HIV-Associated Tuberculosis 17](#__RefHeading___Toc96506751)

[1.2 BACKGROUND 17](#__RefHeading___Toc96506752)

[2.0 OBJECTIVES 18](#__RefHeading___Toc96506753)

[2.1 Primary Objective 18](#__RefHeading___Toc96506754)

[2.2 Secondary Objectives 18](#__RefHeading___Toc96506755)

[3.0 STUDY DESIGN 18](#__RefHeading___Toc96506756)

[3.1 Overview 18](#__RefHeading___Toc96506757)

[3.2 Study Phases 19](#__RefHeading___Toc96506758)

[4.0 PATIENT ELIGIBILITY AND Enrollment 20](#__RefHeading___Toc96506759)

[4.1 Patient Population 20](#__RefHeading___Toc96506760)

[4.2 Inclusion Criteria for Study Entry 20](#__RefHeading___Toc96506761)

[4.3 Exclusion Criteria for Study Entry 21](#__RefHeading___Toc96506762)

[4.4 Exclusion Criteria for Randomization 21](#__RefHeading___Toc96506763)

[4.5 Recruitment and Screening Procedures 22](#__RefHeading___Toc96506764)

[5.0 study phases: STUDY ENTRY, INTERVENTION and post-outcome 23](#__RefHeading___Toc96506765)

[5.1 STUDY ENTRY - STEP 1 23](#__RefHeading___Toc96506766)

[5.1.1 Standard First Line Antituberculosis Therapy 24](#__RefHeading___Toc96506767)

[**5.1.1.1 Drug Regimens, Administration and Duration** 24](#__RefHeading___Toc96506768)

[5.2 STUDY INTERVENTION - STEP 2 25](#__RefHeading___Toc96506769)

[5.2.1 Punctuated Antiretroviral Therapy 26](#__RefHeading___Toc96506770)

[**5.2.1.1 Drug Regimens, Administration and Duration** 26](#__RefHeading___Toc96506771)

[**5.2.1.2 Drug Formulations and Doses** 27](#__RefHeading___Toc96506772)

[**5.2.1.3 Antiretroviral Drug Supply, Distribution, and Pharmacy** 28](#__RefHeading___Toc96506773)

[5.2.1.3.1 Pharmacy 28](#__RefHeading___Toc96506774)

[**5.2.1.4 Anti-retroviral Drug Substitutions and Replacements** 29](#__RefHeading___Toc96506775)

[5.2.2 Randomization 29](#__RefHeading___Toc96506776)

[5.2.3 Blinding 30](#__RefHeading___Toc96506777)

[5.2.4 Modified Directly Observed Therapy 30](#__RefHeading___Toc96506778)

[5.3 POST OUTCOME - STEP 3 31](#__RefHeading___Toc96506779)

[6.0 STUDY OUTCOMES 33](#__RefHeading___Toc96506780)

[6.1 HIV Disease Progression 34](#__RefHeading___Toc96506781)

[6.1.1 Decline in CD4+ Lymphocyte Counts 34](#__RefHeading___Toc96506782)

[6.1.2 AIDS-defining Conditions 34](#__RefHeading___Toc96506783)

[6.1.3 Death 35](#__RefHeading___Toc96506784)

[6.2 Response to Antituberculosis Therapy 35](#__RefHeading___Toc96506785)

[6.2.1 Sputum Culture Conversion 35](#__RefHeading___Toc96506786)

[6.2.3 Microbiologic Cure of TB 35](#__RefHeading___Toc96506787)

[6.2.4 Clinical Cure of TB 35](#__RefHeading___Toc96506788)

[6.2.5 Treatment Failure 36](#__RefHeading___Toc96506789)

[6.2.6 Relapse of Tuberculosis 36](#__RefHeading___Toc96506790)

[6.3 Safety 37](#__RefHeading___Toc96506791)

[6.3.1 Toxicity and Serious Adverse Experiences 37](#__RefHeading___Toc96506792)

[**6.3.1.1 Procedures for Reporting Serious Adverse Experiences** 37](#__RefHeading___Toc96506793)

[**6.3.1.2 Specific Adverse Reactions** 38](#__RefHeading___Toc96506794)

[6.3.1.2.1 Hypersensitivity Reaction (HSR) associated with Abacavir (ABC) 38](#__RefHeading___Toc96506795)

[6.3.1.2.2 Severe Anemia 40](#__RefHeading___Toc96506796)

[6.3.1.2.3 Elevated Liver enzymes 40](#__RefHeading___Toc96506797)

[6.3.2 Development of ARV drug resistance 41](#__RefHeading___Toc96506798)

[6.3.3 Immune Reconstitution Inflammatory Syndrome (IRIS) to TB 41](#__RefHeading___Toc96506799)

[(Paradoxical Reactions) 41](#__RefHeading___Toc96506800)

[6.4 Drug Discontinuation Due to Adverse Drug Reactions 41](#__RefHeading___Toc96506801)

[7.0 CLINICAL AND LABORATORY MEASUREMENTS 41](#__RefHeading___Toc96506802)

[7.1 Screening Measurements 42](#__RefHeading___Toc96506803)

[7.2 Step 1 – Baseline (Study Entry Measurements), Initiation of Antituberculosis therapy 42](#__RefHeading___Toc96506804)

[7.2.1 Baseline Evaluation 42](#__RefHeading___Toc96506805)

[7.2.2 Scheduled Visits During Step 1 43](#__RefHeading___Toc96506806)

[7.3 Step 2 – Measurements during Intervention Period 43](#__RefHeading___Toc96506807)

[7.3.1 Randomization Baseline-Step 2 43](#__RefHeading___Toc96506808)

[7.3.2 Intervention Phase Study Visit 44](#__RefHeading___Toc96506809)

[7.3.3 Post-Study Intervention Follow-up (prior to study outcome) 45](#__RefHeading___Toc96506810)

[7.4 Measurements at Time of and Post- Primary Study Outcome 45](#__RefHeading___Toc96506811)

[7.4.1 Time of Event (Primary Study Outcome) 45](#__RefHeading___Toc96506812)

[7.4.2 Post- Primary Outcome - Antiretroviral Therapy Follow-up Visits 46](#__RefHeading___Toc96506813)

[7.5 Sick Visit Evaluation 47](#__RefHeading___Toc96506814)

[8.0 CLINICAL MANAGEMENT 48](#__RefHeading___Toc96506815)

[8.1 HIV 48](#__RefHeading___Toc96506816)

[8.1.1 Disease Management 48](#__RefHeading___Toc96506817)

[8.1.2 Toxicity Management 49](#__RefHeading___Toc96506818)

[**8.1.2.1 General Guidelines for Adverse Experiences Associated with HIV and ARV** 49](#__RefHeading___Toc96506819)

[**therapy**](#__RefHeading___Toc96506820)

[8.1.2.1.1 Grades 1 and 2 49](#__RefHeading___Toc96506821)

[8.1.2.1.2 Grades 3 and 4 49](#__RefHeading___Toc96506822)

[**8.1.2.2 Antiretroviral Therapy Toxicity and Management** 50](#__RefHeading___Toc96506823)

[8.1.2.2.1 Nausea (with or without vomiting) 50](#__RefHeading___Toc96506824)

[8.1.2.2.2 Anemia/Neutropenia 50](#__RefHeading___Toc96506825)

[8.1.2.2.3 Adverse Experiences to Abacavir (ABC) 51](#__RefHeading___Toc96506826)

[8.1.2.2.4 CNS and Psychiatric Symptoms with Efavirenz (EFV) 53](#__RefHeading___Toc96506827)

[8.1.2.2.5 Pregnancy with Efavirenz (EFV) or Abacavir (ABC) 53](#__RefHeading___Toc96506828)

[8.1.2.2.6 Rash with Efavirenz or Nevirapine 54](#__RefHeading___Toc96506829)

[8.1.2.2.7 Liver Toxicity and Nevirapine 55](#__RefHeading___Toc96506830)

[8.1.2.2.8 Immune Reconstitution Inflammatory Syndrome to TB (Paradoxical Reactions) 55](#__RefHeading___Toc96506831)

[8.1.3 Management of Virologic Failure 56](#__RefHeading___Toc96506832)

[8.1.4 Discontinuation and Re-initiation of Antiretroviral Therapy 56](#__RefHeading___Toc96506833)

[**8.1.4.1 Definitions** 56](#__RefHeading___Toc96506834)

[**8.1.4.2 General Guidelines for Holding, Discontinuing, and Re-starting Study Antiretroviral Drug** 56](#__RefHeading___Toc96506835)

[8.1.4.2.1 Criteria for Holding Antiretroviral Therapy 57](#__RefHeading___Toc96506836)

[8.1.4.2.2 Criteria for Discontinuing Antiretroviral therapy 57](#__RefHeading___Toc96506837)

[8.1.4.2.3 Criteria for Re-starting Antiretroviral Therapy 57](#__RefHeading___Toc96506838)

[8.2 Tuberculosis 58](#__RefHeading___Toc96506839)

[8.2.1 Tuberculosis Disease Management 58](#__RefHeading___Toc96506840)

[8.2.2 Tuberculosis-related Toxicity Management 59](#__RefHeading___Toc96506841)

[**8.2.2.1 General Guidelines - Grading and Management of TB-associated Adverse Events** 59](#__RefHeading___Toc96506842)

[**8.2.2.2 Toxicity due to Standard First Line Anti-tuberculosis Drugs Used in the Study** 59](#__RefHeading___Toc96506843)

[8.2.3 General Guidelines for Holding, Discontinuing, and Re-starting Antituberculosis Treatment 62](#__RefHeading___Toc96506844)

[**8.2.3.1 Criteria for Holding Antituberculosis Therapy** 62](#__RefHeading___Toc96506845)

[**8.2.3.2 Criteria for Discontinuing Antituberculosis Treatment** 62](#__RefHeading___Toc96506846)

[**8.2.3.3 Criteria for Re-starting Antituberculosis Therapy** 63](#__RefHeading___Toc96506847)

[8.3 Management of Pregnancy 63](#__RefHeading___Toc96506848)

[8.4 Concomitant Medications 64](#__RefHeading___Toc96506849)

[8.4.1 Permitted Medications 64](#__RefHeading___Toc96506850)

[8.4.2 Prohibited Medications 65](#__RefHeading___Toc96506851)

[8.4.3 Precautionary Drugs 66](#__RefHeading___Toc96506852)

[8.5 Abnormal Laboratory Values 67](#__RefHeading___Toc96506853)

[8.6 Supervision of Clinical Management 67](#__RefHeading___Toc96506854)

[9.0 DATA INTEGRITY 68](#__RefHeading___Toc96506855)

[9.1 Organizational Structure 68](#__RefHeading___Toc96506856)

[9.2 Data Collection 68](#__RefHeading___Toc96506857)

[9.3 Data Management 68](#__RefHeading___Toc96506858)

[9.4 Data Quality Assurance/ Quality Control 69](#__RefHeading___Toc96506859)

[10.0 STATISTICAL CONSIDERATIONS 69](#__RefHeading___Toc96506860)

[10.1 Sample Size 69](#__RefHeading___Toc96506861)

[10.2 Analytic Strategy 72](#__RefHeading___Toc96506862)

[10.2.1 Descriptive Analysis 72](#__RefHeading___Toc96506863)

[10.2.2 Analytic Strategy by Specific Aim 72](#__RefHeading___Toc96506864)

[**10.2.2.1 Specific Aim 1** 72](#__RefHeading___Toc96506865)

[**10.2.2.2 Specific Aim 2** 75](#__RefHeading___Toc96506866)

[**10.2.2.3 Specific Aim 3** 75](#__RefHeading___Toc96506867)

[**10.2.2.4** **Additional Analyses** 76](#__RefHeading___Toc96506868)

[10.3 Interim Analysis and Stopping Rules 77](#__RefHeading___Toc96506869)

[10.3.1 Interim Analysis 77](#__RefHeading___Toc96506870)

[10.3.2 Stopping Rules 77](#__RefHeading___Toc96506871)

[11.0 REGULATORY CONSIDERATIONS 78](#__RefHeading___Toc96506872)

[11.1 Case Report Forms 78](#__RefHeading___Toc96506873)

[11.2 Monitoring 78](#__RefHeading___Toc96506874)

[11.3 Institutional Review Board (IRB) 78](#__RefHeading___Toc96506875)

[11.4 Data and Safety Monitoring Board 78](#__RefHeading___Toc96506876)

[11.5 Informed Consent 79](#__RefHeading___Toc96506877)

[11.6 Amendments 79](#__RefHeading___Toc96506878)

[11.7 Protocol Deviations 79](#__RefHeading___Toc96506879)

[11.8 Confidentiality 79](#__RefHeading___Toc96506880)

[11.9 Drug Accountability and Record Keeping 80](#__RefHeading___Toc96506881)

[12.0 References 80](#__RefHeading___Toc96506882)

# APPENDICES

[**APPENDIX I. Study Schedule, SCREENING and STEP 1** 86](#__RefHeading___Toc106081675)

[**APPENDIX I. Study Schedule, STEP 2** 87](#__RefHeading___Toc106081676)

[**APPENDIX I. Study Schedule, STEP 3** 88](#__RefHeading___Toc106081677)

[**APPENDIX II. Modified WHO Staging System for HIV Infection and Disease in Adults and Adolescents, including definitive diagnostic methods and presumptive diagnostic criteria for diseases indicative of AIDS (clinical stage IV)** 89](#__RefHeading___Toc106081678)

[**APPENDIX III. MU-JHU CORELAB Testing Algorithm for HIV Screening** 93](#__RefHeading___Toc106081679)

[**APPENDIX IV. Table For Grading Severity Of Adult Adverse Experiences** 93](#__RefHeading___Toc106081680)

[**APPENDIX IV. Table For Grading Severity Of Adult Adverse Experiences** 94](#__RefHeading___Toc106081681)

[**APPENDIX V. Guidelines For Estimating Grade Of Severity For Adverse Experiences Not Otherwise Specified** 101](#__RefHeading___Toc106081682)

[**APPENDIX VI. Paradoxical Clinical Reactions in TB Subjects Treated with** 102](#__RefHeading___Toc106081683)

[**Highly Active Antiretroviral Therapy** 102](#__RefHeading___Toc106081684)

[**APPENDIX VII. Internal Quality Management Plan** 103](#__RefHeading___Toc106081685)

**APPENDIX VIII.** [**Informed Consent for Screening** 117](#__RefHeading___Toc106081686)

**APPENDIX IX.**  [**Study Consent** 126](#__RefHeading___Toc106081687)

[**APPENDIX. X. Sub Analys**es 145](#__RefHeading___Toc106081694)

TABLES AND FIGURES

[Table 1. Summary of evidence for HIV/TB co-pathogenesis. 13](#__RefHeading___Toc54077598)

[Table 2. Steps in Study 19](#__RefHeading___Toc54077601)

[Table 3. Daily dosages for standard first line anti-TB drugs 25](#__RefHeading___Toc54077602)

[Table 4. Study Intervention 26](#__RefHeading___Toc54077603)

[Table 5. Standard Starting Doses and Dose Reductions for Antiretroviral Therapy 28](#__RefHeading___Toc54077604)

[Table 6. Summary of Study Outcomes 34](#__RefHeading___Toc54077605)

[Table 7. Adverse reactions to first line anti-TB drugs 60](#__RefHeading___Toc54077609)

[Table 8. Prohibited Concomitant Medications 65](#__RefHeading___Toc54077610)

[Table 9. CD4+ cell decline before and after TB in Ugandan adults 70](#__RefHeading___Toc54077611)

[Table 10. Schedule of 5 annual interim analyses to monitor disease progression 77](#__RefHeading___Toc54077613)

[Figure 1. Model of the interactions between HIV and MTB. 14](#__RefHeading___Toc53391278)

[Figure 2. Study Design 19](#__RefHeading___Toc53391279)

[Figure 3. Adverse Experiences that Must Be Reported 38](#__RefHeading___Toc53391285)

[Figure 4. Diagnosis of Abacavir-related HSR 39](#__RefHeading___Toc53391286)

[Figure 5. Sample Size Calculation 71](#__RefHeading___Toc53391290)

# Participating Sites

**CLINICAL SITES**

Uganda-Case Western Reserve University Research Collaboration

P.O. Box 663

Kampala, Uganda

Ph: +256 (41) 534-262

Fax: +256 (41) 345-643

Joint Clinical Research Center

Butikiro House, Plot No. 893 Ring Road

Kampala, Uganda

Ph: +256 41 270283

Fax: +256-41-3426

**STUDY IMPLEMENTATION AND COORDINATION**

The Tuberculosis Research Unit

Case Western Reserve University School of Medicine

10900 Euclid Avenue

Cleveland OH 44106

**STUDY DATA AND STATISTICAL CENTER**

University of California, San Francisco

School of Medicine

San Francisco General Hospital

995 Portrero Avenue

Building 80, Ward 84

San Francisco, CA 94110

**STUDY SPONSOR**

Division of AIDS

National Institute of Allergy and Infectious Diseases

National Institutes of Health

Bethesda, MD

**STUDY DONORS**

GlaxoSmithKline

5 Moore Drive
P.O. Box 13398
Research Triangle Park
NC 27709

# Protocol Synopsis

| Hypothesis | *Punctuated* ARV therapy given during treatment of active TB will slow progression of HIV disease in TB patients with CD4+ cells > 350 cells/ μL. |
| --- | --- |
| Objectives | Primary objective:  To determine whether a punctuated six-month course of ARV therapy during treatment of active TB will delay HIV disease progression, as measured by time until CD4+ count falls below 250 cells/μL, the rate of decline in CD4+ cell counts, and incidence of opportunistic infections, among HIV-infected persons with active TB and CD4+ cell count above 350 cells/μL.  Secondary objectives:  a. To determine whether a punctuated six-month course of ARV therapy during treatment of active TB will improve the response to antituberculosis therapy, as measured by one and two month culture conversion, treatment failure, and relapse TB, among patients with active TB and CD4+ cell count above 350 cells/μL  b. To determine whether a punctuated six-month course of highly active ARV therapy is safe when given with antituberculosis therapy as measured by development of ARV drug resistance, toxicity and side effects, and paradoxical reactions. |
| Design | Phase III open-label, randomized, clinical trial. The study intervention will randomize, in a 1:1 manner, 350 eligible patients with active TB and a CD4+ count greater than 350 cells/μL to receive either immediate six-month punctuated course of ARV therapy (N = 175 patients) or delayed ARV therapy (N = 175 patients). |
| Population  & Sample Size | A total of 350 HIV-infected patients, male or female, 13 – 60 years of age, with active pulmonary TB and a CD4+ count greater than 350 cells/μL. |
| Experimental  Regimen | A total of six-months punctuated course of twice daily abacavir 300 mg/ lamivudine 150 mg/zidovudine 300 mg (as Trizivir®) concurrent with first line antituberculosis treatment |
| Comparative  Regimen | Antituberculosis treatment without six-months punctuated ARV therapy. Delayed ARV treatment will be started when the CD4+ count drops below 250 cells/μL. |
| Follow-up | Each patient will be followed for a minimum of 2 years from study enrollment. Total study duration is 5 years |
| Safety Evaluation | Serial medical history and physical examination, laboratory monitoring |
| Virologic Evaluation | Serial samples of plasma with quantitative measurements of viral load |
| Microbiological  Evaluation | Sputum semi-quantitative AFB smear and culture on solid media |
| Primary  Endpoints | HIV disease progression, defined as time to:  1) CD4+ cell count decline to 250/μL or below;  2) development of an AIDS-defining condition (WHO Staging criteria); or,  3) death. |
| Secondary  Endpoints | Response to antituberculosis therapy:  sputum AFB clearance by one and two months  rates of TB treatment failure  rates of TB relapse  microbiologic cure of TB  Safety:  Development of antiretroviral drug resistance  Toxicity and side effects  Paradoxical reactions |
| Statistical &  Analytic Plan | The sample size for the trial is 350 HIV-infected TB patients - 175 randomized to punctuated therapy and 175 randomized to anti-TB treatment only and delayed ARV therapy. The sample size provides a power of 80 – 90% to detect an effect size of 35% or greater for HIV disease progression. This effect size translates into an average delay of about one year before the need to start ARV therapy.  The intent-to-treat approach will be used to compare the rate of HIV disease progression between TB patients randomized to receive either punctuated or delayed ARV therapy.  Analysis – Specific Aim 1  The primary outcome for the study is HIV disease progression. The primary analysis of the study will compare  The failure-time distribution of the treated to the reference group.  The change in CD4+ count over time in each group.  Life-table, failure-time, and repeated measures methods will be used to analyze the data.  Analysis – Specific Aim 2  The effect of ARV treatment on the response to antituberculosis therapy will be evaluated by comparing  The proportion of subjects with negative smears or cultures at one and two months (chi square test).  Treatment failure rates between groups  Relapse tuberculosis rates  Life-table, failure-time, and person-year methods will be used in this analysis.  Analysis – Specific Aim 3  The safety of the study intervention will be examined by summarizing the incidence rate and cumulative proportion of severity grade III/IV adverse events according to each study arm.  Life table and descriptive methods will be used.  Additional analyses  In addition to these analyses, the study will examine viral dynamics, emergence of antiretroviral drug resistance, and reconstitution of the host immune response. |

#

# 1.0 INTRODUCTION

## 1.1 Rational

### **1.1.1 Evidence for Model of HIV/TB Co-pathogenesis**

Conceptual Model of HIV/TB Interactions

The proposed study is founded on a conceptual model for the interactions of HIV and MTB. It is well established that HIV increases the risk for TB^1,2^, alters its clinical presentation^3^, and reduces survival^4^. This study focuses on the interaction in the reverse direction, that is, the effect of TB on HIV infection. In co-infected persons, active TB can initiate a cascade of events leading to accelerated HIV disease progression. During TB, macrophages and CD4+ lymphocytes are activated directly by viable, replicating MTB bacilli and by pro-inflammatory cytokines, such as TNFα, which are over-expressed in HIV-associated TB. In this microenvironment of cellular activation, HIV replication and spread of HIV to uninvolved cells is enhanced. Increased viral replication results in a decline in CD4+ cells and an increased risk for opportunistic infections and death. Other HIV-related conditions (e.g., salmonellosis, herpes simplex virus infections, etc.) may promote viral replication and affect the natural history of HIV, however, the greatest body of evidence in support of the co-pathogenesis model exists for HIV/TB.

The scientific basis for the HIV/TB co-pathogenesis model is strong and supported by a large number of studies that cut across many disciplines (Table 1). Epidemiologic studies conducted in diverse populations and clinical studies from parts of the world where HIV and TB are endemic, including Uganda, demonstrate reduced survival, sustained increases in viral RNA levels, and increased opportunistic infections in HIV-infected persons with TB. These effects are particularly pronounced in persons with preserved immune function as measured by CD4+ T cell counts > 200cells/ml. An experimental simian model of HIV/TB co-infection, demonstrated accelerated SIV disease in animals experimentally infected with mycobacteria and clearly established a temporal relationship between mycobacterial infection and SIV disease progression. *In vitro* studies demonstrate that constituents of MTB can activate latent HIV, promote infection of uninfected T cells and macrophages, and increase HIV heterogeneity. Furthermore, mathematical models of viral dynamics of HIV show that co-infection with MTB increases viral RNA levels and reduces CD4+ lymphocyte counts. Thus, the argument that TB promotes HIV disease progression has biologic plausibility.

**Table 1. Summary of evidence for HIV/TB co-pathogenesis.**

| Discipline | Summary Point | References | |
| --- | --- | --- | --- |
| Epidemiologic and clinical studies | Survival is reduced in HIV+ persons with TB vs without TB | | 5 - 10 |
|  | Incidence of opportunistic infection is greater following TB | | 5, 11 |
|  | Increased HIV RNA level is sustained in TB | | 5, 12 |
|  | Treatment of latent TB is associated with improved survival | | 13 – 15 |
| Experimental SIV macaque model | Mycobacterial infection accelerates the course of SIV/ AIDS and time until death | 16 - 19 | |
| Studies of Immune and Viral mechanisms | TB generates a microenvironment that enhances viral replication and promotes viral diversity | | 20 - 23, 31 - 34 |
|  | This microenvironment is characterized by cellular activation mediated by pro-inflammatory cytokines (e.g., TNFα) and mycobacterial products. | | 21, 25, 29, 30, 34 |
|  | Mycobacterial products and cytokines upregulate HIV replication through NFκB and HIV LTR | | 24, 26 - 28 |
| Mathematical model | Shorter survival and higher viral loads occur after tuberculosis | 35 | |

Although the evidence for the co-pathogenesis of HIV and MTB is scientifically cogent, some aspects remain controversial.^36,37^ The main argument against the model is that TB is merely a marker of progressive immunodeficiency that is not captured by the absolute CD4+ cell count. We acknowledge the underlying logic of this argument, but, to date, the nature of this non-CD4+ dependent immune defect has not been specified. Nevertheless, active TB in HIV-infected persons with high CD4+ counts is associated with an increased mortality compared to HIV-infected persons without TB. Thus, this dually-infected patient population represents a logical target for ARV intervention when CD4+ cell are higher than 250/ml. The synthesis of this data leads to a conceptual model for the study which is outlined in Figure 1.

Interventions can be designed to interrupt the cascade of events triggered by MTB in at least three ways: treat the responsible microbial stimulus – MTB; modulate MTB-mediated cytokine immune activation to reduce the stimulus for viral replication; or inhibit viral replication through ARV therapy. To date, short-term modulation of host immune response with inhibitors of TNF α expression, such as pentoxifylline^38^, thalidomide^39,40^, and prednisolone, have failed to produce lasting effects on CD4+ counts or HIV RNA levels. In addition, treatment of TB alone has had variable effects on viral RNA levels,^12,31^ suggesting that additional modes of intervention are needed to curb the impact of TB on HIV. In the proposed study, we will test whether interventions directed at HIV replication will interrupt the effect of TB on HIV disease progression in patients with high CD4+ cell counts. In this proposal, we will target HIV replication with a six month course of punctuated ARV during a period of maximal immune activation.

**Figure 1. Model of the interactions between HIV and MTB.**

**
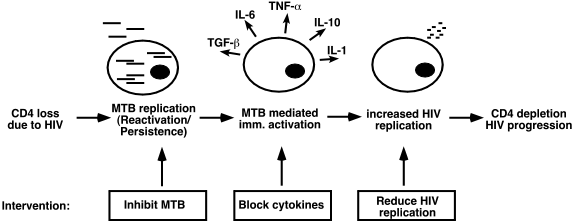
**

###

### **1.1.2 Targeting HIV Replication with Antiretroviral Treatment**

Strategies for ARV Therapy in Developing Countries

The widespread use of potent ARV therapy in the United States and Europe has resulted in a dramatic reduction in AIDS cases, hospitalizations, and AIDS-related mortality.^41^ Several years ago, the prospect of HIV eradication led to very early and aggressive treatment of HIV infected patients. This strategy is now being re-examined in favor of initiating ARV therapy later in HIV disease, before advanced immunosuppression, and by exploring intermittent regimens. There are several reasons for this shift in thinking. First, viral eradication is unlikely with the current generation of drugs because a pool of long-lived, cells latently infected with HIV appear to have a lifespan of many decades.^42^ Second, long-term ARV therapy, although effective in blocking HIV replication, is associated with numerous and serious toxicities. ARV agents are associated with anemia, changes in liver function, peripheral neuropathy, gastrointestinal and central nervous system side effects. In addition, body habitus changes, altered lipid profiles, osteoporosis, lactic acidosis, glucose intolerance can also occur. There has not been enough experience with these drugs to know the long-term consequences of these later effects. They may, however, be harbingers for irreversible, end-organ disease. Some of the metabolic effects of ARV therapy occur very quickly such as glucose intolerance and lipid perturbations. These effects reverse with therapy change or discontinuation and appear to be most associated with the use of protease inhibitors.^43,44^ Other side effects such as body habitus changes evolve over a period of years and therapy switches or discontinuations are associated with subtle improvements, at best. Finally, HIV disease progression and viral suppression do not appear to be jeopardized as long as therapy is initiated before CD4+ cell counts decline to 250/ml.^45,46^

Based on these arguments, therapeutic strategies have therefore shifted from “as much as you can” to “as much as you need.” More emphasis is now placed on delaying therapy until a CD4+ threshold is reached.^47,48^ Higher HIV RNA levels are associated with more rapid declines in CD4+ cell counts, but the CD4+ level remains the best predictor of HIV disease progression as measured by opportunistic infections and death. Clinical trials are underway both in developed and developing countries to determine if intermittent therapy can maintain CD4+ cell counts above a “critical” threshold and reduce drug toxicity at the same time. Thus, the concept of “punctuated” therapy in our proposed study is consistent with this current line of research but is novel because it targets ARV therapy when HIV replication is enhanced from MTB-induced immune activation.

One of the important questions related to our proposed therapeutic strategy is the course of CD4+ cell counts in patients after therapy is interrupted. There are no data on naïve patients with high CD4+ counts who are treated for only six months, who then stop therapy. Three studies have been reported which look at CD4+ decline in heterogeneous cohorts of patients treated for prolonged periods who elect to stop therapy. In the first study, 72 subjects with a median nadir CD4+ count of 272 cells/ml discontinued ARV therapy for a median of 45 weeks. The mean CD4+ count decay was 16 cells/month. The slope of CD4+ decay inversely correlated with the increase of CD4+ count on therapy.^49^ In a second study, 33 patients with CD4+ nadirs above 200/ml discontinued therapy for a mean of 8 months and had a CD4+ decline of 22 cells/month.^50^ In another retrospective study from the EuroAIDS cohort, 16% of 5385 patients interrupted ARV therapy for at least 3 months at a mean CD4+ counts of 242 cells after a mean CD4+ nadir of 130 cells/ml.^51^ There was a loss of 30 cells over 3 months after ARV therapy interruption.

Currently, in Uganda, HIV-infected tuberculosis patients with CD4+ counts < 250/mL are eligible for antiretroviral therapy. Yet, at least one third of HIV-infected patients with active TB have CD4+ cells counts above 350 cells/ml. Starting lifelong ARV therapy immediately in all these patients would result in the treatment of a large proportion of HIV infected patients with high CD4+ cells. While one could argue that all of these patients *should* initiate lifelong ARV therapy (as suggested by the expert panel in the US-DHSS guidelines) to prevent accelerated CD4+ cell decline, it is also possible that a punctuated course of therapy is *sufficient* to delay CD4+ decline. The only way to know if this strategy works is to compare “immediate, punctuated” therapy to “delayed” therapy.

Safety of ARV Therapy during Active TB

Published clinical experience attests to the safety of administering ARV therapy in patients with active TB, although studies to confirm its safety in the developing world are needed.^52^ Transient worsening of TB “paradoxical” reactions (fever, lymphadenopathy) occurs more commonly in TB patients with HIV who receive ARV therapy than in TB patients without HIV. The pathogenesis of these reactions is poorly understood, but is thought to be due to restoration of immune responses to TB antigens producing exuberant inflammatory reactions detrimental to the host. These reactions probably do not require the presence of viable organisms because they can occur beyond the point of sterilization and successful TB treatment. The frequency of these reactions is unknown but may occur in up to a third of patients. The reactions are usually mild and self-limited. There have been no reported deaths due to paradoxical reactions including patients with tuberculous meningitis. Corticosteroids may be required for control of serious inflammatory reaction, although no controlled studies have been performed. In terms of the effect of TB on the success of ARV therapy in TB patients, viral suppression rates appear similar to those observed in patients without TB receiving ARV.

A prescribed drug interruption raises the concern that HIV resistant virus will be selected. In our study, the risk for drug resistance will be minimized because patients will be ARV therapy naïve, will receive a potent triple combination regimen (not a single drug like nevirapine), and will receive a form of directly observed therapy. Several studies have shown that effective viral suppression can be achieved when re-instituting ARV therapy after treatment interruption in naïve patients with initial viral suppression. Nevertheless, recognizing the importance of looking for drug resistance, a secondary objective of our study is to monitor for the presence of resistance as a part of the overall assessment of the punctuated therapeutic strategy.

Benefit of ARV Therapy on response to TB treatment

ARV therapy in HIV-infected patients with active TB may have beneficial effects on the response of patients to antituberculous therapy. Improvement and/or augmentation of host responses to mycobacterial antigens after treatment with ARV therapy is supported by two lines of evidence. First, in our studies of responses to MAC, and in similar studies of responses to PPD in BCG vaccinated patients, in vitro proliferative responses to mycobacterial antigens were restored 3-6 months after initiation of ARV therapy. Second, the frequency of "paradoxical reactions" in HIV-infected patients undergoing treatment of tuberculosis is much higher in patients who are treated with antiretroviral therapy (36%) versus those who are not (7%). The rapid onset of these reactions and response to prednisone suggest heightened immune activation and responsiveness to mycobacterial antigens.

Improved host immune responses may also hasten clearance of MTB from the sputum. Although not studied for MTB, for other opportunistic infections, ARV can speed clearance of the pathogen or resolution of the syndrome.^53^ Resolution of cryptosporidiosis, microsporidiosis, progressive multifocal leukoencephalopathy (PML), *M. avium* bacteremia and Kaposi Sarcoma after the initiation of ARV provide evidence that improving immune function can lead to improved outcome in an acute opportunistic infection.^54-56^ More rapid clearance of MTB has implications not only for the patient but also for transmission to susceptible contacts.

Relevance of Antiretroviral Interventions in Uganda

Uganda has led efforts in Sub-Saharan Africa to access ARV therapy through the Joint Clinical Research Center since 1992 and United Nations assistance programs. Recent reduction in prices have led to an increase of HIV-infected individuals in Uganda and neighboring countries accessing ARV. Currently 6000 patients receive ARV therapy at the JCRC alone, and it is estimated that these numbers will swell to 50000 in the next two years (C Kityo, personal communication). The majority of patients are receiving three-drug regimens including nucleoside reverse transcriptase inhibitors (NRTI) and non- nucleoside reverse transcriptase inhibitors (NNRTI). The Uganda government and health authorities anticipate that ARV therapy will become an increasing part of clinical management of HIV infected patients through new treatment initiatives. They are confident, that at the completion of this study, the results of the trial could be applied in Uganda.

Although the rationale for the proposed study is principally based on arguments not related to drug costs, the outcome has potentially very significant economic implications for Uganda and other resource-limited countries. If a punctuated course of ARV therapy can significantly delay immune decline in patients with TB, then more patients could ultimately be treated with ARV. In other words, if the threshold to initiate ARV therapy is 250/ml, then delaying the time to reach this threshold in patients with HIV/TB will have a major public health impact. We have argued that ARV use will increase in Uganda in the near future, but the resources to support these ARV treatment programs will not be unlimited. Results from this study will assist public health bodies in Uganda, and other areas with high rates of TB, to develop treatment policies that provide access to ARV and preserve quality of life for the maximum number of HIV-infected persons.

### **Treatment of HIV-Associated Tuberculosis**

The treatment of HIV-associated TB is complex. Drug interactions between rifampin and certain classes of antiretroviral agents limits the spectrum of treatment for HIV and increases the risk for drug toxicities. Rifamycins, including rifampin, are metabolized by the hepatic microsomal cytochrome P450 system. Drugs that inhibit CYP3A will result in increased levels of rifampin whereas drugs that induce CYP3A may reduce levels of rifampin. Although the clinical effect of these interactions is not fully known, there is enough information to derive some early guidelines for treatment of HIV-associated tuberculosis. In general, NRTIs are safe to use with TB medications without dose modifications. In this regard, ABC appears to be safe when used with rifampin, although data are limited. NNRTIs, such as efavirenz and nevirapine, may be used with rifampin but may require dose adjustments. In pharmacokinetic studies of nevirapine, rifampin reduces the serum concentration of nevirapine by 37 to 58%, but even with this reduction, the minimal concentration of nevirapine was sufficient to inhibit viral replication. (Oliva, AIDS 2003; 17(4): 637 – 8). In a pharmacokinetic study of efavirenz, a dose of 800 mg of efavirenz plus rifampicin was equivalent to a does of efavirenz 600 mg without rifampicin. Rifabutin is a viable alternative to rifampin as it mitigates, but does not eliminate, drug-drug interactions. In the developing world, however, access to rifabutin is limited, so regimens with rifampin are preferred. Protease inhibitors may be used with rifabutin, but not generally with rifampin. Recent information suggests that ritonovir, with or without saquinavir, may be used safely with rifampin.

## 1.2 Background

The World Health Organization estimates that 10 million HIV-infected individuals died of TB during the decade of the 1990’s. The effect of HIV has been especially devastating in sub-Saharan Africa where the annual TB incidence is nearly 200 cases per 100,000 population and between 50 and 75 % of new TB cases are HIV-infected. HIV-1 infection increases the mortality associated with active TB despite adequate response to antituberculous therapy.^4,57-58^ The one-year mortality from TB in HIV-infected individuals is over 4 times the rate in HIV seronegative cases.^57^ Deaths early in treatment are often due to TB, but later deaths are attributable to advancing HIV disease.^4^

Uganda was among the first countries in Sub-Saharan Africa to recognize the impact of HIV in the country.^59^ The overall seroprevalence of HIV in Uganda has decreased over the past decade and is now about 8.5%, but in sentinel populations, such as patients with TB, the seroprevalence remains over 50%.^60^ It is estimated that approximately 400,000 Ugandan adults are co-infected with HIV and MTB. In 1996, the TB case rates in Uganda were 143/100,000 with a total of 27,122 cases reported. Of these, 4218 cases of smear-positive pulmonary TB were from the Kampala district (incidence rate of 545 cases/100,000) and half were likely to be HIV-infected. Based on data collected from our screening and recruitment at the Uganda National Tuberculosis and Leprosy Programme, we have found the average CD4+ count at the time of presentation to be 375 cells/ml. Thus, the burden of HIV-associated TB is high in Kampala, and there is a large population that would benefit from the proposed intervention.

# 2.0 OBJECTIVES

## 2.1 Primary Objective

To determine whether a punctuated six-month course of ARV therapy during treatment of active TB will delay HIV disease progression, as measured by time until CD4+ count falls below 250/µL and the rate of decline in CD4+ cell counts, among HIV-infected persons with active TB and CD4+ cell count above 350 cells/µL.

## 2.2 Secondary Objectives

1. To determine whether a punctuated six-month course of ARV therapy during treatment of active TB will improve the response to antituberculous therapy, as measured by one and two month culture conversion, treatment failure, and relapse TB, among patients with active TB and CD4+ cell count above 350 cells/µL.
2. To determine whether a punctuated six-month course of highly active ARV therapy is safe when given with antituberculosis therapy as measured by development of ARV drug resistance, toxicity and side effects, and paradoxical reactions.

# 3.0 study design

## 3.1 Overview

This study will be an open-label, randomized, clinical trial. The study intervention will randomize, in a 1:1 manner, 350 eligible patients with active TB and a CD4+ count greater than 350 cells/µL to receive either immediate six-month punctuated course of ARV therapy (abacavir, zidovudine, lamivudine; N = 175 patients) or anti-TB treatment only with delayed ARV therapy (N = 175 patients). Immediate, punctuated ARV treatment will be given daily for six months, concurrent with antituberculous treatment, and then interrupted until the CD4+ count drops below **250/µ**L, or a WHO stage IV AIDS defining condition develops **or a definitive diagnosis of a severe bacterial infection that meets WHO Stage 3 classification.**. Patients assigned to punctuated ARV treatment will receive a full six months of ARV treatment, regardless of when they complete anti-TB treatment. Delayed ARV treatment will be started when and if the CD4+ count drops below **250/µ**L, or a WHO stage IV AIDS defining condition develops **or a definitive diagnosis of a severe bacterial infection that meets WHO Stage 3 classification.** (Appendix II), or death, whichever occurs first. This study will have an 80% power to detect an effect size in the primary outcome of 35% or greater. This translates into an average delay of about one year before the need to start ARV therapy. Secondary outcomes will include: 1) response to antituberculous therapy as measured by sputum clearance by one and two months and rates of TB relapse; 2) safety as measured by drug toxicities and development of drug-resistance to ARVs. All patients will be offered ARV therapy when CD4+ count falls below 250/µL during the study period. The study has been designed according to the CONSORT recommendations for parallel-group randomized trials.^61^

**Figure 2. Study Design**

Punctuated ARV

Therapy during

TB Treatment

R

HIV+ Active TB

CD4+ > 350 cells

No Punctuated

ARV Therapy

R = Random Treatment Assignment

## 3.2 Study Phases

Patients progress through the study in three steps, each representing distinct milestones in the study. Patients that meet all initial screening inclusion and exclusion criteria enter Step 1 of the study without randomization. In Step 1, patients are started on antituberculosis treatment, and followed according to Step 1 study schedule (Appendix I). Patients are followed on a weekly basis for the first 4 weeks and assessed for contraindications for starting a Trizivir-based punctuated ARV regimen. All patients are evaluated at week 1 for readiness to start punctuated antiretroviral therapy and have a pre-ARV baseline assessment. Patients who meet all eligibility criteria for randomization, based on contraindications to starting ARV therapy, progress to Step 2 (Appendix 1). In Step 2, patients are randomized into one of two study arms: 1) punctuated ARV therapy with continued anti-TB treatment; or, 2) standard anti-TB treatment only. Patients that have contraindications to starting the Trizivir-based punctuated ARV therapy at week 2 will not be randomized, and may be reassessed at week 3 and week 4. If contraindications have resolved, between 2 and 4 weeks, and patient meets criteria for randomization, the patient will be randomized and initiated on the appropriate study arm regimen. If by the end of week 4 the patient still exhibits contraindications for starting the Trizivir-based ARV regimen, they will not be randomized into study arm but will remain on study follow-up and eligible to enter Step 3 of the study. Patients remain in Step 2 until development of a primary study outcome. When the primary outcome is confirmed, the patient is evaluated for contraindications to ARV therapy (Appendix 1, Step 3) and then may enter Step 3 if eligible. Patients remain in Step 3 until the study ends, at which time each patient is closed out.

**Table 2. Steps in Study**

| Step 1 | Study entry (pre-randomization)  Start antituberculosis therapy  Assess contraindications to starting ARV therapy  Determine eligibility for randomization |
| --- | --- |
| Step 2 | Randomization  Pre-ARV assessment of viral/ immunologic measures  Intervention as assigned  Achieves primary outcome |
| Step 3 | Assess contraindications to starting ARV therapy  Pre-ARV assessment of viral/ immunologic measures  antiretroviral therapy |

## 4.1 Patient Population

# 4.0 PATIENT ELIGIBILITY AND Enrollment

HIV-infected adults, aged 13 to 60 years, with smear-positive active pulmonary TB and preserved immune function as measured by CD4+ lymphocyte count will be screened for the study and started on anti-TB treatment with standard short course chemotherapy (2EHRZ/4HR).

Study entry will be open to men and women patients of all ethnic and tribal backgrounds. Historically, approximately 55% of the TB patients treated at the clinical site are men. The ethnic make-up of the population is diverse and representative of the tribes in central Uganda with Baganda the most common (60%). The socioeconomic status of patients attending this clinic is generally in the middle to lower strata. Thus, the gender, ethnic and socioeconomic background of the subjects recruited for this study is expected to be representative of TB patients and of the population most affected by TB in Uganda. A screening database will be kept of all screened patients providing informed consent for screening excluded from the study to monitor the recruitment/enrollment process and assess generalizability of study findings.

## Inclusion

## Inclusion Criteria for Study Entry

Screening evaluations to determine eligibility must be completed within 14 days prior to study entry unless otherwise specified.

1. AFB Smear-positive or culture-positive, presumed pulmonary TB
2. Patients, male or female, aged 13-60 years
3. HIV seropositive (at any time)
4. CD4+ count > 350 cells/ml
5. Residence within 20 km of Kampala

6 Female study volunteers of reproductive potential (defined as girls who have reached menarche or women who have not been post-menopausal for at least 24 consecutive months, i.e. who have had menses within the preceding 24 months, or have not under-gone surgical sterilization (e.g. hysterectomy, bilateral oophorectomy, or salpingotomy) must have a negative serum or urine pregnancy test performed within 14 days prior to study entry.

1. If participating in activity that could lead to pregnancy, the female study volunteer must use a reliable method of contraception while receiving protocol-specified medication(s) and for 6 weeks after stopping the medications(s).
2. If the female volunteer is not of reproductive potential (girls who have not reached menarche, women who have been post-menopausal for at least 24 consecutive months, or women who have undergone surgical sterilization, salpingotomy) she is eligible without requiring the use of contraception.

7 Ability and willingness of subject or legal guardian to provide informed consent for study participation

## 4.3 Exclusion Criteria for Study Entry

Screening evaluations to determine eligibility must be completed within 14 days prior to study entry unless otherwise specified.

1. History of current TB treatment of greater than 7 days duration
2. History of prior tuberculosis or history of previous tuberculosis treatment
3. Extra-pulmonary tuberculosis without pulmonary involvement based on clinical and radiologic evaluation
4. Multi-drug resistant MTB, as assessed by resistance to both Rifampicin and Isoniazid
5. WHO Stage IV AIDS-defining condition
6. Hemoglobin < 9.5 gm/dl
7. Absolute neutrophil count < 1000 cells
8. Serum aspartate aminotransferase (AST) > 2 times upper limit of normal
9. Serum creatinine > 2.5 times upper limit of normal
10. Karnofsky performance status < 60%,
11. Weight < 40 Kg
12. Allergy or adverse reaction to study medications
13. Positive serum or urine pregnancy test for women of childbearing potential
14. Breastfeeding women
15. Prior ARV therapy, with the exception of single dose nevirapine mother-to-child transmission regimen
16. Receipt of investigational agents (NOTE: HIV vaccines are allowed).
17. Current treatment with Ribavirin or Doxorubicin

## 4.4 Exclusion Criteria for Randomization

Randomization into the study intervention will occur between weeks 2 and 4 following study entry and initiation of standard anti-TB therapy. The study population will be restricted to patients exhibiting none of the following contraindications to starting the punctuated antiretroviral intervention during this assessment period:

1. Fever > 38.0˚ C;
2. WHO stage IV AIDS-defining condition;
3. Weight < 40 Kg;
4. CD4+ count < 350 cells/ml at screening
5. AST > 2.0 times upper limit of normal;
6. Creatinine > 2 mg/dL;
7. Hemoglobin < 9.5 g/dL;
8. Absolute neutrophil count < 1000 cells;
9. Positive serum or urine pregnancy test for women of childbearing potential;
10. Current treatment with Ribavirin or Doxorubicin;

Laboratory values must be within the acceptable range within 14 days prior to randomization to Step 2.

Post randomization exclusion:

Final determination of eligibility will be made after review of drug susceptibility testing results on an initial sputum isolate and results of all sputum cultures. Subjects found to have multi-drug resistant strains will be excluded from randomization.  These subjects will be withdrawn from the study but treatment and follow-up will be provided for MDR TB.

## 4.5 Recruitment and Screening Procedures

350 HIV-infected patients (age 13-60 years) with their initial episode of acid-fast bacilli smear-positive or culture-confirmed pulmonary TB will be recruited for Screening through the National Tuberculosis Control Programme at Old Mulago Hospital and TB Clinics throughout Kampala, Uganda.

Patients presenting with suspected TB will be evaluated at the Tuberculosis Treatment Center and will be identified over a three year period. From this pool of suspected TB cases, a consecutive sample of patients will be screened through the following standard evaluation:

1. Informed consent for the screening evaluation
2. Establish diagnosis of active tuberculosis through medical history and physical examination including Karnofsky score assessment and weight
3. Chest radiography (posterior-anterior view)
4. Confirmatory HIV testing algorithm per Core Lab standard procedures (Appendix III)
5. Three sputum samples for acid-fast bacilli staining and mycobacterial culture
6. MTB drug susceptibility testing done on these positive screening samples
7. Pregnancy test (urine or serum *β*-HCG)
8. Complete blood count with differential
9. Liver function tests (AST and serum bilirubin)
10. Serum creatinine
11. Serum glucose
12. Measurement of CD4+ lymphocyte percent and absolute count
13. Store MTB isolates for DNA fingerprinting, if necessary

This screening evaluation is completed by Ugandan medical officers and may take 2 – 5 days to complete. Patients who meet all of the inclusion criteria and none of the exclusion criteria will be referred for Step 1 – study entry, and start antituberculosis therapy. Trained and experienced HIV counselors will provide pre- and post-test counseling.

We project that the study will recruit 3 new cases per week on average so that the full sample size of 350 cases will be recruited, enter the study and be randomized within three years. The Tuberculosis Clinic of the National Tuberculosis and Leprosy Control Programme at Old Mulago Hospital evaluates and treats over 1200 patients per year. Patients who are interested in participating in research projects are referred to the Tuberculosis Clinics of the Uganda-CWRU Research Collaboration located across the street from Mulago hospital. The Tuberculosis research clinic evaluates about 600 TB patients per year presenting for the first time with pulmonary TB. Of these patients, approximately 50% of them are co-infected with HIV-1. Since the average CD4+ count at presentation is 372 cells/ml, we expect that about 50% of the HIV-infected cases will be eligible for the study.

There are two levels of consent for the study. At screening each screened subject will give written informed consent for the screening process to determine study entry eligibility (Appendix VIII). Upon confirmation of study entry eligibility, each subject will give informed consent for participation in the study (Appendix IX).

Referral of Non-enrolled Cases

HIV seronegative cases and HIV seropositive subjects who are not eligible for or decline participation in the study will be referred to the National Tuberculosis and Leprosy Programme where they will receive anti-tuberculosis treatment free-of-charge. HIV-infected patients will also be referred to either HIV/AIDS clinics in the Kampala area where they will receive the standard of care for HIV/AIDS or to another study of HIV care and management, if available. A list of these clinics will be provided to the patient and referral made to the clinic of choice. Patients will also be informed of HIV/AIDS clinics in the city where antiretroviral therapy is available and may be purchased by the patients. At this time, access to inexpensive antiretroviral treatment is still limited in Kampala, though Ugandan health officials are optimistic that access will improve in the near future. Currently, a month’s course of treatment costs a minimum of $28 USD. Antiretroviral treatment is currently available at the Joint Clinical Research Center (JCRC), Mildmay Center for AIDS care, Nsambya Hospital, clinics supported by non-governmental agencies, and private practitioners’ clinics in Kampala.

A listing of current studies, in the greater-Kampala area, recruiting HIV-infected patients and providing antiretroviral treatment will be maintained by the study personnel. Patients ineligible for the study will be referred to available studies as appropriate.

**5.0 STUDY PHASES: STUDY ENTRY, INTERVENTION AND POST-OUTCOME**

##

## 5.1 Study Entry – Step 1

All patients in this study will receive routine treatment for tuberculosis (see Section 5.1.1). The experimental intervention in this study is the use of punctuated antiretroviral therapy for six months during treatment for tuberculosis. Because standard anti-TB drugs represent the background treatment, these drugs will not be considered study drugs. When patients have been found to be eligible for the study and have given informed consent, all patients will be started on antituberculosis treatment, given daily for a total of six months and will have the following baseline assessment:

1. Informed consent for study
2. Medical history and physical examination, including weight
3. HIV RNA Roche Amplicor
4. PBMC storage/ immune function (Flow cytometry to assess cell surface activation markers, EliSpot to assess CD4 and CD8 T cell specificity to HIV and microbial peptides, Whole blood assays to determine antigen specific response to HIV and microbial antigens)
5. Plasma & Buffy Coat storage

Patients will be evaluated weekly while in Step 1. At one week from study entry all patients will be assessed for readiness to start on the Trizivir-based punctuated ARV therapy with the following evaluation:

1. Medical history and physical examination, including weight
2. New Diagnosis and hospitalization survey
3. Adverse event survey
4. Pregnancy test (urine or serum *β*-HCG)
5. Complete blood count with differential
6. Liver function tests (AST and serum bilirubin)
7. Serum creatinine
8. Adherence to antituberculosis therapy
9. Quality of life survey
10. Disease specific diagnostic tests

The patient will be scheduled to return to the clinic 1 week after this evaluation (two weeks after the start of TB therapy) to be randomized or identified as having contraindications (see Section 4.4). If the patient has contraindications to starting the Trizivir-based ARV intervention they will be scheduled to return to the clinic in another week (Week 3) and be reassessed.

Thus, all patients evaluated at week 1 will be scheduled to return to the clinic at week 2, and may be randomized or reassessed for randomization at that time; those patients that have contraindications at week 2 may be scheduled to return to the clinic for randomized or reassessment at week 3; and those patients reassessed at week 3 will be scheduled to return to the clinic and may be randomized at week 4.

If, during week 4, contraindications to starting Trizivir® persist, patients will not be randomized to study intervention but will continue on anti-TB treatment and study follow-up as described in Step 2. These patients will continue to be eligible for Step 3 of the study, delayed antiretroviral therapy if they experience a primary study outcome.

### **5.1.1 Standard First Line Antituberculosis Therapy**

#### **5.1.1.1 Drug Regimens, Administration and Duration**

All patients will receive standard short-course antituberculous therapy recommended by the Centers for Disease Control and Prevention and the World Health Organization. This treatment includes isoniazid, rifampin, and pyrazinamide and ethambutol for two months followed by isoniazid and rifampin for four months (2RHZE/4RH). Rifabutin will not be substituted in this study because it is not currently available in Uganda. Furthermore, given the limited use of rifabutin in Africa, the generalizability of the study will be increased with the use of rifampin. A patient is considered to have completed therapy when the six month, standard short-course antituberculosis treatment is taken with demonstrated clinical or bacteriologic response to treatment.

**Dosage and Administration:**

Dosages of standard anti‑TB drugs will be modified by body weight as follows:

**Table 3. Daily dosages for standard first line anti-TB drugs**

| **Drug** | **Body weight**  **< 50 kg** | **Body weight**  **≥ 50 kg** |
| --- | --- | --- |
| Isoniazid | 300 mg/d | 300 mg/d |
| Rifampicin | 450 mg/d | 600 mg/d |
| Ethambutol^a^ | 800 mg/d | 1200 mg/d |
| Pyrazinamide | 1.5 gm/d | 2 gm/d |

^a^ ≈ 15 mg/kg/d

If a patient who initially weighs < 50 kg gains weight during treatment such that he or she weighs ≥ 50 kg, the patient’s doses will be increased according to the dosing chart. Pyridoxine 50 mg PO will be administered daily to all subjects during TB treatment to prevent INH-related neurotoxicity.

## 5.2 Study Intervention – Step 2

When eligibility for Step 2 is confirmed, patients will be randomized to receive either immediate six-month punctuated ARV treatment consisting of abacavir, zidovudine, and lamivudine (as Trizivir®) or continuation of anti-TB treatment only with delayed, open-label ARV treatment regimen. Randomization will occur at the time the patient is assessed and meets criteria to start the Trizivir-based punctuated ARV intervention.

Patients will be evaluated at entry into Step 2 with the following pre-ARV (baseline): assessment

1. HIV RNA Roche Amplicor;
2. Plasma storage;
3. PBMC storage/ immune function (Flow cytometry to assess cell surface activation markers, EliSpot to assess CD4 and CD8 T cell specificity to HIV and microbial peptides, Whole blood assays to determine antigen specific response to HIV and microbial antigens)

and, initiated either on the study intervention of six-month punctuated course of Trizivir® or continue with anti-TB treatment only, according to randomized assignment.

Antiretroviral therapy will be started regardless of random assignment in the intervention period when either 1) two CD4+ counts (within 3 months) drop to < 250/µL or 2) an AIDS-defining condition develops as defined by WHO stage IV AIDS-defining condition or a definitive diagnosis of a severe bacterial infection that meets WHO Stage 3 classification (Appendix II). Subjects who do not reach a study outcome during the study period will not enter Step 3 and therefore not be offered daily ARV therapy.

### **5.2.1 Punctuated Antiretroviral Therapy**

#### **5.2.1.1 Drug Regimens, Administration and Duration**

Table 4. Study Intervention

| Study Arm | N | Intervention Period | Post-Outcome |
| --- | --- | --- | --- |
| Punctuated Therapy | 175 | 2RHZE/4RH QD + 6 Months ABC/3TC/ZDV (as Trizivir®) BID | Delayed antiretroviral therapy* |
| Delayed Therapy | 175 | 2RHZE/4RH QD | Delayed antiretroviral therapy* |
| *ARVs will be offered during study period to any patient when CD4+ count falls below 250/µL or when an AIDS-defining condition occurs. Patients who do not develop a primary study outcome during the study period will not be offered delayed ARV therapy. | | | |

Punctuated Therapy Arm:

Patients enrolled in the punctuated therapy arm will receive oral abacavir 300 mg/ lamivudine 150 mg/zidovudine 300 mg (as Trizivir®) twice daily with or without food. As each subject is enrolled, the study site must ensure that (1) the subject receives the **Trizivir**® warning card and medication guide, (2) the designated health care provider (e.g., physician, study nurse coordinator, or pharmacist) reviews the signs and symptoms of a hypersensitivity reaction (HSR) with the subject, and (3) the subject verbalizes an understanding of the steps to take in the event of a suspected HSR, including when and how to contact the study physicians. Study physicians will be available 24 hours per day through office or cellular telephone. Individual medication components of Trizivir®, will be available for dose adjustment.

For subjects who start Trizivir® but are found to be intolerant of it, alternative regimens will be available as described in Section 5.2.1.4, Antiretroviral Drug Substitutions and Replacements.

Patients who completed the punctuated antiretroviral intervention without experiencing ABC HSR will be re-started on antiretroviral therapy when, and if, during the study period the CD4+ count drops below 250/ml or with the development of an AIDS-defining condition. At this time, patients will be started on self-administered ARV therapy in accordance with Ugandan National Treatment Guidelines, or as directed by the Principal Investigator or designee (see Section 5.2.1.4 Antiretroviral Drug Substitutions and Replacements). A safety assessment will be done before starting antiretroviral therapy, and will include a CBC, creatinine, serum AST and bilirubin, and urine pregnancy test, and will determine whether there are any contraindications (hypersensitivity reactions to chosen medication) or precautions to antiretroviral therapy.

Delayed Anti-retroviral Therapy Arm:

Patients allocated to the delayed therapy arm will receive antiretroviral therapy when, and if, during the study period the CD4+ count drops below 250/ml or with the development of a WHO Stage IV AIDS-defining condition or a definitive diagnosis of a severe bacterial infection that meets WHO Stage 3 classification (Appendix II). At this time, patients will be started on self-administered ARV therapy in accordance with Ugandan National Treatment Guidelines, or as directed by the Principal Investigator or designee. A safety assessment will be done before starting antiretroviral therapy, and will include a CBC, creatinine, serum AST and bilirubin, and urine pregnancy test, and will determine whether there are any contraindications (hypersensitivity reactions to chosen medication) or precautions to antiretroviral therapy.

**Patients Ineligible for Intervention after 4 weeks of TB Treatment**

Patients who have persistent contraindications to starting ARV therapy after 4 weeks in Step 1 will remain in the study. Patients in this group are followed because of our ethical obligation to them after enrolling them in the study. These patients will be treated for active tuberculosis and monitored for primary study outcomes according to the schedule for assessments outlined in Step 2. These patients will be registered to Step 2 but will not be randomized nor will they be included in the primary analysis of the study. They will be eligible for entry into Step 3 of the study if, during the study period, their CD4 count drops below 250/ml or an AIDS- defining condition develops as defined by WHO stage IV HIV infections or a definitive diagnosis of a severe bacterial infection that meets WHO Stage 3 classification (Appendix II)

#### **5.2.1.2 Drug Formulations and Doses**

Trizivir®

Abacavir 300 mg/lamivudine 150 mg/zidovudine 300 mg (ABC/3TC/ZDV, Trizivir™) will be administered orally BID with or without food. Store at 25° (77° F); excursions permitted to 15°-30°C (59°-86°F).

Combivir®

Lamivudine 150-mg/zidovudine 300-mg combination tablets (3TC/ZDV, Combivir®) will be administered orally BID, with or without food. Store at 25° (77°); excursions permitted to 15°-30°C (59°-86°F).

EPIVIR®

Lamivudine (3TC) 150-mg and 300-mg tablets (EPIVIR®) will be administered orally as 150 mg BID with or without food. 300 mg tablets are available for once a day, nighttime dosing. Store at 25°C (77°F), excursions permitted to 15°-30°C (59°-86°F).

RETROVIR®

Zidovudine (ZDV) 300-mg tablets (RETROVIR®) will be administered 300 mg BID with or without food. 100-mg capsules are available for dose reduction. Store at 15° to 25°C (59°-77°F) and protect from moisture.

ZIAGEN®

Abacavir sulfate (ABC) 300-mg tablets (ZIAGEN®) will be available as single agent and administered at a dose of 600 mg QD, with or without food. Store at controlled room temperature of 20° to 25°C (68°-77°F)

Sustiva®/ or Stocrin™

Efavirenz (EFV) 200-mg capsules (Sustiva® or Stocrin™): Store at 25°C (77°F); excursions permitted to15°-30°C (59°-86°F). EFV 600 mg will be administered orally QD, on an empty stomach (i.e., 1 hour before or 2 hour after a meal), unless the patient is taking rifampin concurrently when 800 mg will be given. Bedtime dosing may improve tolerability of nervous system symptoms. During punctuated therapy, the dose will be given during the day as modified directly observed therapy. For those on punctuated therapy who experience CNS side effects, EFV may be administered at bedtime. For patients taking rifampin, dose reduction from 800-mg to 600 mg QD EFV is allowed if CNS symptoms persist. Women of reproductive potential if participating in activity that could lead to pregnancy must use two reliable methods of contraception while receiving Efavirenz and for 6 weeks after Efavirenz is discontinued.

Viramune® Nevirapine (NVP) 200-mg tablets: Store at room temperature, 15°-30°C(59°-86°F). NVP 200 mg will be administered orally QD for the first 2 weeks (lead-in), then 200 mg orally BID thereafter. The designated health care provider should review the signs and symptoms of NVP-related hypersensitivity and hepatitis with the subject prior to dispensing NVP. Subjects should contact their medical officer if they develop rash, or signs and symptoms of hypersensitivity or hepatitis. If rash occurs during the lead-in, do not escalate the dose until the rash has resolved. After reaching full dose, if NVP dosing is interrupted for >7 days NVP should be started with the lead-in of 200 mg orally QD for 2 weeks, then 200 mg orally BID. NVP may be taken with or without food.

**Table 5. Standard Starting Doses and Dose Reductions for Antiretroviral Therapy**

| DRUG | INITIAL DOSE | DAILY DOSE | REDUCED DOSE | DAILY REDUCED DOSE |
| --- | --- | --- | --- | --- |
| EFV | 600 mg QD | 600 mg | No Reduction | No Reduction |
| EFV^1^ | 800 mg QD | 800 mg | 600 mg QD | 600 mg |
| ZDV | 300 mg BID | 600 mg | 200 mg BID | 400 mg |
| 3TC | 150 mg BID | 300 mg | No Reduction | No Reduction |
| ABC | 300 mg BID | 600 mg | No Reduction | No Reduction |
| NVP | 200 mg QD x 14 d, then 200 mg BID | 200 mg x 14 d, then 400 mg | No Reduction | No Reduction |

**^1^ While receiving rifampin**

#### **5.2.1.3 Antiretroviral Drug Supply, Distribution, and Pharmacy**

ABC/3TC/ZDV (as Trizivir®), 3TC/ZDV (as Combivir®), ABC (as Ziagen®), 3TC (as Epivir®), and ZDV (as Retrovir®) will be provided by GlaxoSmithKline. EFV (as Sustiva or Stochrin) will be purchased locally from the Joint Clinical research Center with institutional funds. Nevirapine (as Viramune®) will be purchased locally from the Joint Clinical research Center with institutional funds.

##### 5.2.1.3.1 Pharmacy

The antiretroviral study medications will be shipped to and stored at the pharmacy at the Joint Clinical Research Center (JCRC) in Kampala, Uganda. The JCRC has extensive experience in procuring, storing, and dispensing antiretroviral medications. The JCRC pharmacy is staffed by trained pharmacists with experience in dispensing antiretroviral medications.

This pharmacy will be responsible for maintaining an inventory of study medication, ensuring proper storage, and producing timely reports of medication use. The pharmacist will be responsible for packaging antiretroviral therapy in packets with labels for individual study patients. These packets will be given to study home visitors for directly observed therapy. Empty packets will be returned to the pharmacy.

#### **5.2.1.4 Anti-retroviral Drug Substitutions and Replacements**

If a patient develops intolerance to Trizivir® at any point in the study or fails to achieve viral suppression on therapy (Section 8.1.3), then an alternative regimen will be selected by study physicians based on the type of intolerance observed, the causative agent, and availability of medication. Single drug formulations of abacavir, lamivudine, and zidovudine will be available for dose adjustments of combination ABC/3TC/ZDV (as Trizivir®) as clinically indicated. Efavirenz and 3TC/ZDV (as Combivir®) will be available to substitute for ABC/3TC/ZDV (Trizivir) when abacavir-related HSR or intolerance occurs. Efavirenz will be substituted for ZDV or 3TC when dose limiting toxicity occurs to these medications. Nevirapine will be available for substitutions of efavirenz for treatment-limiting toxicity and substitution of efavirenz during pregnancy as clinically indicated. Nevirapine cannot be substituted for efavirenz or any component of Trizivir in the following circumstances: women with CD4>250 OR men with CD4>400 at the time of proposed NVP initiation. See section 5.2.1.2 for formulations and initial dosing.

If a patient develops intolerance to ABC during punctuated therapy, he/she will receive efavirenz (800 mg) + ZDV (300 mg) + 3TC (150 mg) BID. Efavirenz (EFV) will be administered at a dose of 800 mg when patient is receiving rifampin because of the drug interaction between EFV and rifampin. Efavirenz otherwise will be administered at a dose of 600 mg. EFV will be given with modified directly observed therapy, usually during the day.

Options for third-line study drugs will be at the study physician’s discretion. Alternate choices will be made based on drug availability, cost, participants prior ARV drug experience, and the study physician’s clinical judgment.

These drugs are currently available in Uganda through the Joint Clinical Research Center and will be purchased on a case-by-case basis.

### **5.2.2 Randomization**

Treatment intervention will be randomly allocated to patients with active TB when patients enter Step 2 of the study. Eligible TB patients will be randomized with an allocation ratio of 1:1 to receive either punctuated ARV therapy or anti-TB treatment only with delayed ARV therapy. Randomization will be done with a permuted-block size of 16 to reduce the likelihood of unbalanced study arms in terms of size and prognostic factors. The assignment to study arm will be made by the UCSF data center to reduce the likelihood of inappropriate enrollment or treatment allocation.

Before the trial begins a list of computer-based random numbers will be generated using a pseudo-random number generator. These randomly allocated numbers will be used to assign eligible study patients to study arm. This list will be retained at the data center at UCSF. Once eligibility for study entry into Step 1 has been determined through the screening process, a form that contains a summary of study entry inclusion and exclusion criteria will be sent to the statistical center. Patients enter Step 1 where they are started on anti-TB treatment and monitored for contraindications to start the Trizivir-based ARV intervention regimen. All patients will be assessed beginning at week 1 and up to week 3 for contraindications to starting intervention. Upon determination of eligibility criteria for randomization, a form with criteria for randomization will be sent to the UCSF data center. Study entry and randomization criteria will be verified and a treatment assignment made according to the list of random numbers. Upon randomization, patients enter Step 2 of the study where they are initiated into study arm. Patients with contraindications for starting Trizivir-based punctuated ARV therapy without resolution during the 2 to 4 week period will be registered to Step 2, will not be randomized, will not be included in the primary analysis of the study, but will remain on study follow-up. They will be eligible for entry into Step 3 of the study, if during the study period, their CD4+ count drops 250/ml or an AIDS- defining condition develops as defined by WHO stage IV HIV infections or a definitive diagnosis of a severe bacterial infection that meets WHO Stage 3 classification (Appendix II).

### **5.2.3 Blinding**

The ARV intervention will be open-label. In the proposed design, study subjects and physicians alike will be aware of the treatment assignment at the initiation of the intervention. The initiation of the intervention will occur between 2, and 4 weeks following initiation of standard anti-TB treatment. A placebo controlled design was not chosen because of ethical concerns and the sensitivities regarding placebo trials in the study population.

To address detection and reporting bias, we will use a reproducible, laboratory-based endpoint based on CD4+ measurements that is not subject to interpretation by investigators or subjects. Laboratory specimens will be identified by study subject ID numbers. Laboratory staff performing safety, microbiological and immunologic assays will be blinded to treatment arm assignment. Clinicians will be masked to treatment assignment during chest X-ray reading.

To minimize bias further, the frequency and duration of follow-up will be similar in both study arms. Outcome data will be collected and recorded in a standard manner for all study subjects. A Clinical Review Committee composed of senior level faculty at Case Western Reserve University, University California San Francisco, Makerere University review and classify all study outcomes (Table 6) in a blinded manner. Losses to follow-up will be recorded; reasons for the loss to follow-up will also be recorded. The potential bias from unbalanced treatments of intercurrent infections will be minimized by the use of a standard protocol for the treatment of these conditions.

### **5.2.4 Modified Directly Observed Therapy**

In this study, modified directly observed therapy (mDOT) will be used for both antituberculous and the punctuated ARV intervention. When medication is given by directly observed therapy, a health care provider, or designee, observes the patient swallow all pills and records that the dose was given. Directly observed therapy is an approach used to ensure adherence with study medication.

In this study, a team of home health visitors will deliver both the antituberculous and ARV treatment to study patients. The team of home visitors will be supervised by a single Ugandan medical officer. Home visitors will work with patients to determine a convenient time and place to deliver the medication Monday through Saturday. Home visitors will observe the patients swallow their pills six days a week. Thus, all antituberculous doses will be observed on these days and half of the punctuated ARV doses will be observed on these days because of the twice daily dosing. One day per week, patients will take their medication without medical supervision. Distribution of ARV medication will be strictly monitored to prevent misuse of study medication.

In the event that a patient defaults on a scheduled appointment with the home visitor, the home visitor will notify the study coordinator and begin to trace the patient.

The treatment supervisor will hold the patient’s anti-TB medications and treatment card. The patient and the treatment supervisor will both initial (or mark) the treatment card for each supervised dose^63^. The patient will initial (or mark) the treatment card for each unsupervised dose.

Before starting antiretroviral therapy, all patients will receive intensive counseling about antiretroviral therapy, especially ABC. They will be fully informed of the method of drug delivery through mDOT and will be counseled about the important of adherence with prescribed medication. They will be informed that unscheduled pill counts will be done to assess adherence to unsupervised doses.

As part of the mDOT, patients will be given verbal and written material about the toxicities associated with each medication including ABC. The educational material will give specific information about ABC HSR. This information will describe the early signs and symptoms of HSR and the risk of continuing with ABC in the face of these symptoms. Patients will be instructed to contact the study personnel using the 24 hour per day telephone service or present to the clinic immediately. The patients will be told that a study doctor will make the decision to stop the medication, but it is their responsibility to notify the study doctors of the signs and symptoms of ABC HSR. Patients will be instructed that they may not take ABC at any time in the future if ABC HSR occurs. This counseling will be done by study personnel who are fully knowledgeable about the risks of antiretroviral therapy, especially ABC. All patients will be given a card that indicates that they are receiving ABC as part of this study. The card contains instruction for how to reach a study doctor. It will also instruct health care providers to notify study personnel regarding the health of the patient.

## 5.3 Post Outcome – Step 3

During the study period, patients in Step 2 may develop a primary study outcome. If this event happens, the outcome will be confirmed and patients will be evaluated for safety of starting antiretroviral therapy. Patients who have experienced the main study outcome and who are eligible to start antiretroviral therapy will enter Step 3. For patients with a primary outcome but not eligible for antiretroviral therapy, because of contraindications to starting ARV treatment, they will remain in Step 2 and have the safety assessments repeated monthly or as clinically indicated until they are found eligible for antiretroviral treatment.

Patients will be assessed for the primary study outcome when CD4+ count falls below 250/ml or upon the development of a WHO stage IV AIDS-defining condition or a definitive diagnosis of a severe bacterial infection that meets WHO Stage 3 classification (Appendix II). To confirm the CD4 outcome, the CD4+ cell count will be repeated within 1 month after the initial measure below 250. If two measurements are <250 cells/μL within a 3month time period, then the primary outcome has been confirmed. The outcome of an AIDS-defining condition must be confirmed by definitive or presumptive criteria (Appendix II). To confirm the outcome the following will be performed (see Appendix I, Step 3):

1. Medical history and physical examination, including weight
2. Adverse Event Survey
3. New diagnosis and hospitalization survey
4. Pregnancy test (urine or serum)
5. CD4+ lymphocyte count
6. CBC with differential
7. Liver function tests (AST and serum bilirubin)
8. Serum creatinine
9. Disease specific diagnostic test

These results will be valid for a period of two weeks and may be used for the safety evaluation, if within the acceptable range and time frame (2 weeks).

While the primary outcome is being confirmed patients will also be undergoing a pre-ARV safety assessment. If the evaluations below were not previously within the acceptable range or outside the two week time frame the evaluation that was not acceptable will be repeated. This ARV safety assessment may include results from the following (see Appendix I, Step 3):

1. Medical history and physical examination, including weight
2. Adverse Event Survey
3. New diagnosis and hospitalization survey
4. Pregnancy test (urine or serum)*
5. Complete blood count with differential
6. Liver function tests (AST and serum bilirubin)
7. Serum creatinine

**for those subjects that produce a positive pregnancy test see section 8.3 for specific ARV therapy assignment*

These results will be valid for a period of two weeks and will be used to determine whether there are any contraindications to starting antiretroviral therapy The following are relative contraindications to starting antiretroviral therapy for patients participating in this study (see section 4.4):

1. Fever > 38.0˚ C;
2. Weight < 40 Kg;
3. AST > 2.0 times upper limit of normal;
4. Creatinine > 2 mg/dL;
5. Hemoglobin < 9.5 g/dL;
6. Absolute neutrophil count < 1000 cells;
7. Current treatment with Ribavirin or Doxorubicin

Patients who have one or more of these contraindications to starting antiretroviral therapy will be evaluated and treated for the conditions causing abnormal values. Patients will be started on antiretroviral therapy once the acute condition has resolved. For patients who have persistent abnormal values that are attributed to progressive HIV infection, they will be eligible to start antiretroviral therapy with an appropriate regimen. Patients who are eligible to start antiretroviral therapy formally enter Step 3 and will have a pre-ARV baseline assessment, which includes (see Appendix I, Step 3):

1. HIV RNA Roche Amplicor
2. Quality of life assessment
3. Plasma storage
4. PBMC storage/ immune function (Flow cytometry to assess cell surface activation markers, EliSpot to assess CD4 and CD8 T cell specificity to HIV and microbial peptides, Whole blood assays to determine antigen specific response to HIV and microbial antigens)

Patients who satisfy all indications for therapy and safety evaluations will be started on ARV therapy. Once starting ARV treatment, patients will be followed per schedule of evaluations, Appendix I, Step 3.

If a patient is taking punctuated therapy at the time of a primary outcome, they will be evaluated for virologic failure (Section 8.1.3). Patients will be evaluated to determine whether a new protocol indicated regimen should be initiated or whether current regimen should be held, discontinued, or re-started. See section 5.2.1.4 and 8.1.4.

Patients who are not eligible for starting antiretroviral therapy at the time of the primary outcome will be reassessed in a month. If the contraindications for antiretroviral treatment have resolved, the patient will enter Step 3 as described above. If contraindications to antiretroviral treatment persist, then the patient will be followed and assessed for ARV safety eligibility monthly or as clinically indicated. Patients will be reassessed for contraindications at these monthly visits, or sooner if clinically indicated. If a patient is found eligible for antiretroviral therapy at any of these follow-up visits, then the patient will enter Step 3 as described above.

Patients will be started on self-administered ARV therapy in accordance with Ugandan National Treatment Guidelines, or as directed by the Principal Investigator or designee.

# 6.0 STUDY OUTCOMES

The main outcome of the study will be progression of HIV disease (Table 6). The time of the main outcome will be defined as the interval from enrollment until an outcome event first occurs. In the case of CD4+ counts, when a subject has met the criteria for primary outcome, the date of the first of two CD4+ counts < 250/ml will be used. Secondary outcomes will be response to antituberculosis therapy and safety.

For analysis, a Clinical Review Committee, chaired by the PI or designee, will be formed at the start of the study to review and classify all outcomes of the trial in a blinded way. Outcomes will be recorded on standard data collection forms for entry into an outcomes database. For clinical care on-site, medical officers will operationalize outcome definitions so that patients may be treated in a timely manner.

Table 6. Summary of Study Outcomes

| Study Aim | | Measurement |
| --- | --- | --- |
| Primary Objective | Aim |
| HIV Disease Progression | 1 | Time interval from enrollment until 2 CD4+ count < 250/*µ*L within a 3 month time period , rate of decline in CD4+ T cell counts  - Development of AIDS-defining condition (WHO Staging System; not including recurrent TB)  - Definitive diagnosis of a severe bacterial infection that meets WHO Stage 3 criteria  - Death |
| Secondary Objectives |  |  |
| Response to Antituberculous therapy  Safety | 2  3 | MTB Sputum Conversion  Cure of active TB  TB Treatment failure  TB Relapse  Grade III/IV adverse reactions  Paradoxical reactions  HIV Drug resistance |

## 6.1 HIV Disease Progression

The main outcome of the study will be progression of HIV disease. Disease progression will be defined as the time interval from enrollment until one of the three following events, whichever occurs first: CD4+ count decline to two values less than 250/*µ*L within a three month time period, development of an AIDS-defining condition, or death.

### 6.1.1 Decline in CD4+ Lymphocyte Counts

The CD4+ T cell number will be monitored regularly during the study. When a CD4+ count is found to be below 250 cells/ml, the test will be repeated within one month because of inherent variability in CD4+ count. When any two values are below 250 cells/mL within a three month time period, then the patient will be considered to have reached a study outcome. The date from the first of these two values drawn within the 3 month window will be used in the analysis. CD4+ count is chosen as the primary outcome because it remains the best single immunologic marker for the risk of opportunistic infection.

### **6.1.2 AIDS-defining Conditions**

Patients will be evaluated regularly by medical officers for inter-current HIV-associated conditions**.** When a patient develops one or more WHO Stage 4 AIDS defining conditions, or a definitive diagnosis of a severe bacterial infection that meets WHO Stage 3 classification, that patient will be considered to have reached a study outcome. A modified version of the WHO Staging system for HIV Infection will be used to define presumed and confirmed inter-current conditions (Appendix II). The staging system in this study will exclude TB from the definitions given; that is, patients will be staged according to infections or conditions other than TB. Definitive diagnosis of a WHO Stage 3 severe bacterial infection requires isolation of bacteria from an appropriate clinical specimen from an ordinarily sterile site.

### **6.1.3 Death**

Death and date of death will be obtained from family members and caregivers through standard verbal autopsy and local hospitals (death certificate and autopsy reports). If the date of death is not known or cannot be ascertained through medical records, it will be assigned a value of the 15^th^ in the month death occurred. All deaths will be reported as serious adverse events.

##

## 6.2 Response to Antituberculosis Therapy

### **6.2.1 Sputum Culture Conversion**

The proportion of patients with MTB sputum conversion (from positive to negative) per treatment group will be determined at months 1 and 2. For an individual patient, sputum culture conversion will be defined as the absence of mycobacterial growth on solid media without a subsequent positive sputum culture during the first 16 weeks of therapy. Subjects with all sputum cultures negative at 2 consecutive time points who are clinically stable or improving and unable to produce sputum will be considered to have converted their sputum at the date of the first of the two specimens. Patients with one negative sputum culture after a period of treatment, who are clinically stable or improving and unable to produce sputum thereafter, will be considered to have converted their sputum culture at the time of the first specimen.

**6.2.2** **Completion of Antituberculosis Therapy**

Patients who take two months of isoniazid, rifampin, pyrazinamide, and ethambutol AND four months of isoniazid and rifampin will be classified as completing a course of antituberculosis therapy. Effective antituberculosis therapy is defined as a regimen in which the organism is susceptible to two or more antituberculosis agents.

### **6.2.3 Microbiologic Cure of TB**

Microbiologic cure of TB is defined as negative mycobacterial cultures on 2 or more consecutive occasions including one culture done after 5 months of therapy**.**

### **6.2.4 Clinical Cure of TB**

Clinical cure of TB is defined as a patient who (1) has completed anti-TB therapy (6.2.2); (2) has a negative smear or culture when last tested; (3) no new lesions on chest x-ray; and (4) no clinical signs or symptoms of active TB.

### **6.2.5 Treatment Failure**

A culture-positive treatment failure is defined as a patient who is receiving anti-TB treatment and has at least one sputum mycobacterial culture growing at least 5 colonies of *M. tuberculosis* (MTB) on solid medium after completing 4 or more months of effective anti-TB treatment. Patients who default before completing anti-TB treatment and return later with culture-positive tuberculosis will be termed failures after non-adherence.

Patients will be retreated in accordance with applicable national TB program guidelines and drug susceptibility testing results.

### **6.2.6 Relapse of Tuberculosis**

A relapse case of tuberculosis is defined as incident tuberculosis that develops in a patient who completed a course of antituberculosis treatment AND had clinical and/or microbiologic evidence for response to treatment in the earlier episode. Relapse cases may be defined as either clinical or culture-confirmed relapses.

- A clinical relapse is defined as a patient with clinical and/or radiographic evidence of recurrent tuberculosis not confirmed by a positive mycobacterial culture during follow-up after successful completion of initial anti-TB treatment (defined in 6.2.2).

- A culture-confirmed relapse is defined as a patient with clinical and/or radiographic evidence of recurrent tuberculosis that is confirmed with one or more positive cultures for mycobacterial growth. A mycobacterial culture is considered positive as follows: 1) a mycobacterial culture has at least 5 colonies of *M. tuberculosis* (MTB) on solid medium; 2) a mycobacterial culture

growing fewer that 5 colonies of MTB on solid medium with the same RFLP pattern as strain causing initial disease; or 3) mycobacterial growth detect using BACTEC.

Clinical evidence of active tuberculosis includes clinical signs (cough, consolidation, lymphadenopathy, wasting, fever, meningitis, ascites, etc.) and symptoms (cough, fever, weight loss, anorexia, headache, abdominal distention, etc.). Radiographic evidence includes an abnormal chest X-ray with findings consistent with TB.

In some unusual instances, positive mycobacterial cultures are found in patients without signs or symptoms of active TB in whom routine screening is performed. In these cases, patients with isolated single positive sputum cultures will not be considered to be relapses unless, as described above, they have signs or symptoms consistent with active tuberculosis and respond to antituberculosis treatment. For patients without signs or symptoms who have multiple positive cultures, these patients will be considered probable cases.

All recurrent cases of tuberculosis will be reviewed by the Clinical Review Committee who will be blinded to the study arm. This panel will classify all recurrent cases according to a standard set of criteria. Using this classification system, recurrent cases are classified as definite (culture-confirmed), probable (signs, symptoms, and laboratory findings consistent with active tuberculosis without a positive culture AND response to antituberculosis therapy), possible (signs or symptoms of active tuberculosis without confirmatory laboratory findings AND response to antituberculosis therapy), and unlikely (none of the above categories). Where reviewers differ in the classification of cases, a consensus decision is made after all reviewers meet to discuss the case. For the purposes of this study, only definite and probable cases will be categorized as relapses.

Restriction fragment length polymorphism (RFLP) analysis will be used to determine whether the relapse is the result of reactivation of previous disease (matched RFLP patterns) or of reinfection (non-matched RFLP patterns).

## 6.3 Safety

In this study, patient safety will take the highest priority in the routine assessment and management of study patients. Safety will be evaluated using adverse reactions as graded by standard NIAID/ACTG toxicity tables. All Grade IV adverse events will be reported to the Institutional Review Boards at respective institutions (Section 11.3), the study sponsor, and the Data Safety and Monitoring Board (Section 11.4).

### **6.3.1 Toxicity and Serious Adverse Experiences**

Serious adverse experiences will be identified through 1) active case finding using a standard protocol for clinical evaluation and laboratory investigation and 2) passive case finding when patients seek medical care in a sick visit. Serious adverse experiences (SAE) (21 CFR 314.80) are any adverse experience occurring at any dose that results in: death, a life-threatening adverse drug experience (as defined by Grade 4 Adverse Event), inpatient hospitalization or prolongation of an existing hospitalization, a persistent or significant disability or incapacity, or a congenital anomaly or birth defect. Other events not resulting in death, that are not life-threatening or that do not require hospitalization may be considered to be SAE when, based on appropriate medical judgment, they jeopardize the patient and may required medical or surgical intervention to prevent one of the above outcomes.

Suspected abacavir-related HSR will be reportable through a separate form designed to record information on all suspected cases. If the syndrome is confirmed or meets the criteria as a SAE, then a separate SAE report will be completed as described below. While not defined by U.S. regulations as a serious adverse experience, pregnancy will be reported to the study sponsor as an SAE using the SAE reporting form, thus allowing collection and tracking of this information in a systematic manner. Reporting of pregnancy in this way ensures proper referral to antenatal clinics and accordance of contraindicated medication.

#### **6.3.1.1 Procedures for Reporting Serious Adverse Experiences**

All serious adverse experiences as defined in Section 6.3, occurring in study subjects during the course of the study must be reported promptly to the Principal Investigator, the Data Coordinating Center at UCSF, the TB Research Unit Coordinating Center at CWRU and the Study Sponsor (DAIDS) at the following address:

The DAIDS Regulatory Compliance Center (RCC)/Safety Office,

e-mail address: RCCSafetyOffice@tech-res.com,

telephone number 301-897-1709,

Fax number: 301-897-1710.

The study site physician also is responsible for reporting any SAE arising during the conduct of the study to the local IRB.

Requirements for adverse experiences requiring reporting are listed and summarized in the following flow diagram:

**Figure 3. Adverse Experiences that Must Be Reported**

**Does this finding represent a change from baseline?**

🡫

**YES NO**🡪 *No report is required*

🡫

**Is the finding *SERIOUS*?** (Did one of the following events result?)

- Death
- Life-threatening adverse drug experience, i.e., Grade 4 adverse event
- Required hospitalization or prolonged and ongoing hospitalization
- Persistent or significant disability or incapacity
- Congenital anomaly or birth defect.
- Jeopardized the patient or subject and required medical or surgical intervention to prevent one of the outcomes listed above

🡫

**Yes No**🡪 *No report is required*

🡫

***Report on Serious Adverse Experience form within one business day of learning of event***

#### **6.3.1.2 Specific Adverse Reactions**

Both antituberculosis therapy and antiretroviral therapy are associated with well-described adverse reactions. These adverse reactions will represent study outcomes and are specifically defined below. Management of these conditions will be described in Clinical Management (Section 8.0).

##### 6.3.1.2.1 Hypersensitivity Reaction (HSR) associated with Abacavir (ABC)

In clinical studies, approximately 3%-5% of patients receiving ABC develop an HSR that in rare cases has proved fatal. HSR is characterized by the appearance of symptoms indicating multi-organ/body system involvement. Symptoms usually appear within the first 6 weeks of starting treatment with ABC (median time to onset is 11 days), but may occur at any time while on therapy.

This section describes the diagnostic criteria that will be operationalized in the study. The clinical management of the syndrome is described under Clinical Management (Section 8.1.2.2.3). In this protocol we will distinguish the clinical diagnosis made at the time of evaluation from the final classification of a suspected HSR that will be used in the analysis. The clinical diagnosis will be made by the study team on-site using information available at the time of the evaluation. For the analysis, the Clinical Review Committee will examine all available data including the clinical course after ABC is withdrawn. This later classification will be used in the analysis of the study.

Clinical Diagnosis of ABC HSR

The diagnosis of HSR will be suspected when a patient receiving ABC presents to a routine or sick visit with a combination of fever, rash, gastrointestinal symptoms, worsening respiratory symptoms, neurologic symptoms. Although there is no diagnostic test available to confirm the clinical diagnosis of HSR, a routine medical evaluation will be performed including vital signs and physical examination looking for clinical characteristics of HSR. Standard laboratory evaluation will include CBC with differential count, serum chemistries, creatinine, and liver function tests. If liver enzymes are abnormal, then viral serologies for hepatitis A and B will be drawn.

Because the signs and symptoms of HSR are non-specific, and may be confused with side effects of rifampin, other HIV conditions, or paradoxical reactions, the evaluations of these patients will be as extensive as possible to allow for accurate diagnosis at the time of the event and proper classification for analysis.

HSR is defined as a clinical syndrome with findings from two or more of the following groups of signs or symptoms occurring simultaneously in a patient receiving ABC:

- Fever > 38°C
- Rash
- One or more gastrointestinal symptoms: nausea, vomiting, abdominal pain, diarrhea
- One or more respiratory symptoms not directly referable to active TB: dyspnea, non-productive cough, sore throat
- One or more musculoskeletal symptoms: myalgia, arthralgia
- One or more neurologic symptoms: headache, paresthesias

All suspected cases of HSR will be reviewed with on-site investigator during the clinic visit in which the diagnosis is suspected. A course of diagnosis and management will be delineated at that time. A decision tree to evaluate the occurrence of an abacavir-related HSR is summarized in the following flow diagram:

**Figure 4. Diagnosis of Abacavir-related HSR**

**Does the patient present with two or more of the following symptoms?**

- Fever > 38°C
- Rash
- One or more gastrointestinal symptoms (nausea, vomiting, abdominal pain, diarrhea) occurring while treating with Trizivir
- One or more respiratory symptoms not directly referable to tuberculosis: dyspnea, non-productive cough, sore throat
- One or more systemic musculoskeletal symptoms: myalgia, arthralgia (involving multiple joints)
- One or more neurologic symptoms: severe headache, paresthesias not attributable to INH
- Side effects of other antiretroviral medications.

🡫

**YES NO**🡪 *HSR not suspected*

🡫

**Suspected *HSR***

🡫

- Present case of suspected HSR to on-site study clinician before patient leaves clinic
- Determine if ABC needs to be discontinued. (Note: A patient will only continue on ABC if syndrome is likely to be of another etiology and the patient is available for close follow-up)
- Place patient on “ABC HSR Evaluation and Follow-up Protocol” (Section 8.1.2.2.3)
- Report suspected HSR on ABC HSR Reporting form or Serious Adverse Event form as clinically indicated within one business day of event

Final Diagnosis of ABC HSR for Analysis

At the stage of classification, HSR is defined as a clinical syndrome with findings from two or more of the following groups of signs or symptoms occurring simultaneously in a patient receiving ABC:

- Fever > 38°C
- Rash
- One or more gastrointestinal symptoms (nausea, vomiting, abdominal pain, diarrhea) occurring while treating with Trizivir
- One or more respiratory symptoms not directly referable to active TB: dyspnea, non-productive cough, sore throat
- One or more systemic musculoskeletal symptoms: myalgia, arthralgia (involving multiple joints)
- One or more neurologic symptoms: severe headache, paresthesias not attributable to INH

**AND**

Exclusion of the following conditions as a cause of the symptom complex:

- Chemical (especially rifampin) or viral hepatitis
- Gout, arthritis or arthralgias caused by pyrazinamide
- Exacerbation of underlying tuberculosis
- Immune reconstitution syndrome (IRIS) to TB (paradoxical reaction)
- HIV-related condition such as cryptococcal meningitis, oral thrush, esophageal candidiasis, cryptosporidiosis/microsporidiosis, etc.

**AND**

Resolution of symptoms with the discontinuation of ABC. Resolution of symptoms within 48 to 72 hours will constitute the strongest evidence for HSR

##### 6.3.1.2.2 Severe Anemia

Grade 3 anemia is defined as a hemoglobin level between 6.5 and 6.9 g/dl. Grade 4 anemia is defined when the hemoglobin < 6.5 g/dl.

##### 6.3.1.2.3 Elevated Liver enzymes

Grade 3 elevation in liver enzymes is defined as a serum AST level > 5 to 10 times the upper limit of normal. Grade 4 elevations in liver enzymes is defined as a serum AST level > 10 times the upper limit of normal.

### **6.3.2 Development of ARV drug resistance**

Viral drug resistance will be examined in specimens from patients with confirmed failure to suppress HIV RNA < 200 copies/ml after the six month course of antiretroviral therapy on step 2 or after six months of antiretroviral therapy on Step 3, and, for a random subset of 20 patients 2 months after completing punctuated therapy. In these patients, pre-treatment plasma will also be assayed for drug resistance. Genotypic analysis performed at the Joint Clinical Research Center Virology laboratory will be used to evaluate for drug resistance. Resistance will be defined according to IAS/USA guidelines.

### **Immune Reconstitution Inflammatory Syndrome (IRIS) to TB**

### **(Paradoxical Reactions)**

Immune Reconstitution Inflammatory Syndrome (IRIS) to TB (paradoxical reactions) are defined as (1) new persistent fevers (temperature ≥ 38.6°C) which develop after the initiation of HAART and which last for more than 1 week without an identifiable source (e.g., urine and sputa testing, and other procedures when clinically indicated); OR (2) marked worsening or emergence of intrathoracic lymphadenopathy, pulmonary infiltrates; OR (3) worsening or emergence of cervical adenopathy on serial physical examinations, OR worsening of other tuberculosis lesions or manifestations, such as cutaneous peritoneal or central nervous system (CNS) inflammatory pathology. (Appendix VI)

All cases of suspected paradoxical reaction will be reviewed in a blinded way by the Clinical Review Committee (see Section 5.2.3) and classified for analysis. Since it will not be possible to differentiate HSR from paradoxical reaction, some cases will be classified as having both syndromes.

## 6.4 Drug Discontinuation Due to Adverse Drug Reactions

As a study outcome, drug discontinuation due to adverse drug reaction is defined when study drug(s) cannot be re-instituted safely.

Drug discontinuation for causes other than adverse drug reactions (e.g., withdrawal from study, loss to follow-up, etc), will be defined as the date the patient withdrew from the study.

# 7.0 CLINICAL AND LABORATORY MEASUREMENTS

As indicated in Section 3.2, patients progress through the study in a series of Steps. This section describes the evaluations made at each Step in the study. Measurements include serial medical history and physical examination, chest X-ray, sputum microscopy and culture, urine pregnancy test, and laboratory assessment of initial metabolic, hematologic, hepatic, and renal function (see Study Follow‑up and Laboratory Testing Schedule - Appendix I). Sputum specimens will be collected using standardized procedures. Three separate Schedules of Evaluations are provided corresponding to Screening and Steps 1 (Study Entry and Initiation of standard Anti-TB therapy), Step 2 (Randomization and Study Intervention) and Step 3 (Primary Study Outcome and Delayed Antiretroviral Therapy).

Serum AST and total bilirubin should also be drawn at any time a patient is on anti-TB or antiretroviral therapy if the subject develops symptoms of hepatitis (nausea, vomiting, malaise, jaundice, or fatigue). CBC and platelet count should be repeated if there are clinical signs of anemia (pallor, hematemesis, hematochezia, etc.) or thrombocytopenia (petechiae, bruises, etc.). If a patient develops hepatotoxicity requiring study drug discontinuation, the following evaluation is suggested: patient interview with review of ethanol intake and use of any other hepatotoxic drugs such as acetaminophen, and obtain hepatitis serologies as appropriate [hepatitis A Ig M antibody (HAV IgM), hepatitis B surface antigen (HbsAg), hepatitis C antibody (HCV) Ab].

## 7.1 Screening Measurements

The following screening evaluations (Appendix I - Study Schedule, SCREENING and STEP 1) will be performed for all subjects.

1. Screening informed consent
2. Medical history and physical examination, including weight
3. Posterior-anterior chest X-ray (if not performed within the previous 14 days)
4. Confirmatory HIV testing algorithm (Appendix III)
5. Sputum semi-quantitative AFB smear and semi-quantitative culture (colony forming unit assay on solid media) X 3
6. Sputum MTB drug susceptibility testing done on these positive screening samples
7. Urine or serum β-HCG pregnancy test (females of child bearing potential only)
8. CBC, differential white blood cell and platelet count
9. Serum aspartate aminotransferase (AST), serum total bilirubin
10. Serum creatinine
11. Serum glucose (for clinical management only)
12. CD4+ lymphocyte count and percent
13. Storage of two pre-treatment MTB isolates at –70 °C. for later DNA RFLP genotyping

## 7.2 Step 1 – Baseline (Study Entry Measurements), Initiation of Antituberculosis therapy

### **7.2.1 Baseline Evaluation**

Patients who meet all of the inclusion and none of the exclusion criteria for study entry enter Step 1 and undergo a baseline evaluation prior to starting antituberculosis treatment. The evaluation is summarized below (see Appendix I, Step 1):

1. Informed consent
2. Medical history and physical examination, including weight
3. Plasma HIV RNA by Roche Amplicor
4. Peripheral blood mononuclear cell (PBMC) storage, 2 aliquots/ Immune Function (Flow cytometry to assess cell surface activation markers, EliSpot to assess CD4 and CD8 T cell specificity to HIV and microbial peptides, Whole blood assays to determine antigen specific response to HIV and microbial antigens)
5. Plasma & Buffy Coat storage, 5 ml.

### **7.2.2 Scheduled Visits During Step 1**

Antituberculosis therapy will be initiated following the Baseline visit. Patients will be evaluated weekly during Step 1. At Week 1 patients will first be assessed for eligibility for randomization, with exclusions based on contraindications to starting a Trizivir-based punctuated ARV therapy. Between Weeks 2 and 4, patients that are found eligible for randomization will receive treatment assignment and will progress to Step 2 at time of randomization. Patients, who do not meet criteria for randomization at Week 2, may be reassessed for eligibility for randomization at Weeks 2 or 3. The weekly evaluations will consist of (see Appendix I, Step 1):

1. Interim history and physical exam including vital signs, intercurrent illnesses, concomitant medications, adverse experiences, and major TB and HIV-related signs and symptoms (Week 1; Week 2, 3 if previous weeks results not in acceptable range for randomization; sick visits as indicated)
2. New diagnoses and/or hospitalizations since the last study visit (Week 1; Week 2, 3 if previous weeks results not in acceptable range for randomization; sick visits as indicated)
3. Adverse experiences survey (Week 1; Week 2, 3 if previous weeks results not in acceptable range for randomization; sick visits as indicated)
4. Sputum semi-quantitative AFB smear and culture on solid media (as indicated)
5. Urine or serum β-HCG pregnancy test (females of child bearing potential only) (Week 1; Week 2, 3 if previous weeks results not in acceptable range for randomization; sick visits as indicated)
6. CBC with differential (Week 1; Week 2, 3 if previous weeks results not in acceptable range for randomization; sick visits as indicated)
7. Serum aspartate aminotransferase (AST), serum total bilirubin (Week 1; Week 2, 3 if previous weeks results not in acceptable range for randomization; sick visits as indicated)
8. Serum creatinine (Week 1; Week 2, 3 if previous weeks results not in acceptable range for randomization; sick visits as indicated)
9. CD4+ lymphocyte counts (as indicated)
10. Adherence questionnaire (anti-TB therapy, not done on randomization visit)
11. Disease specific diagnostic test (as indicated)
12. Quality of life assessment (Week 1)

**Laboratory values must be within acceptable range within 14 days prior to randomization.**

Patients who do not meet the criteria for randomization will remain in the study, but will not be randomized to intervention. Instead, they will be treated for active tuberculosis with antituberculosis therapy and will be followed according to evaluations described in Step 2 (see appendix I, Step 2).

## 7.3 Step 2 – Measurements during Intervention Period

### **7.3.1 Randomization Baseline-Step 2**

For patients screened and eligible for randomization, they will have a baseline randomization evaluation including:

1. Plasma HIV RNA by Roche Amplicor
2. Plasma storage, 5 ml.
3. Peripheral blood mononuclear cell (PBMC) storage, 2 aliquots/ immune function (Flow cytometry to assess cell surface activation markers, EliSpot to assess CD4 and CD8 T cell specificity to HIV and microbial peptides, Whole blood assays to determine antigen specific response to HIV and microbial antigens)

Assessments from Step 1 may be used for pre-antiretroviral baseline status if they were completed within two weeks before randomization.

### **7.3.2 Intervention Phase Study Visit**

Following treatment assignment and initiation of study intervention, patients will be evaluated one and two weeks after initiation of ARV treatment and then monthly during study intervention phase (see Appendix I, Step 2).

1. Medical history and physical examination including weight, major TB and HIV-related signs and symptoms and primary study outcomes (Week, 2, Months 1, 2, 3, 4, 5, 6, and as indicated)
2. New diagnoses and/or hospitalizations since the last study visit (Week 2, Months 1, 2, 3, 4, 5, 6, and as indicated)
3. Posterior-anterior chest X-ray (8 weeks, and between 22 and 24 weeks)
4. Adverse experiences survey (Week 2, months 1, 2, 3, 4, 5, 6, and as indicated)
5. Sputum semi-quantitative AFB smear and culture on solid media X 2 (between 4 & 6 weeks, 8 weeks, and between weeks 18 & 20 from the start of anti-TB treatment)
6. MTB drug susceptibility (as clinically indicated)
7. Urine or serum β-HCG pregnancy test (females of child bearing potential only) (Week 2, Months 1, 2, 3, 4, 5, 6, and as indicated)
8. CBC, differential white blood cell and platelet count (Week 2, Months 1, 2, 3, 6, and as indicated)
9. Serum aspartate aminotransferase (AST), serum total bilirubin (Week 2, Months 1, 2, 3, 6, and as indicated)
10. Serum creatinine (as clinically indicated)
11. CD4+ lymphocyte counts (Weeks 2, Months 1, 3, 6, and as clinically indicated)
12. Plasma HIV RNA by Roche Amplicor (Weeks 2, Months 3 and 6, and as clinically indicated)
13. Adherence Questionnaire for antituberculosis and antiretroviral therapy (Week 2, Months 1, 2, 3, 4, 5, 6, and as indicated)
14. Quality of life assessment (month 6)
15. Urine INH testing (Week 2, Months 1, 2, 3, 4, 5, 6)
16. Plasma storage (Weeks 1, 2, Months 1, 2, 3 and 6)
17. PBMC storage/ immune function (Week 2, Months 3, and 6)
18. Store MTB isolate for DNA fingerprinting (All isolates from positive cultures of suspected relapse cases will be stored at -70° C for DNA finger printing with the patient’s initial isolate, as clinically indicated)
19. Disease specific diagnostic test (as clinically indicated)

### **7.3.3 Post-Study Intervention Follow-up (prior to study outcome)**

Each subject will undergo the following examinations during follow‑up after completion of the six-month ARV Intervention Phase (see Appendix I, Step 2). After completing the intervention phase, patients will be seen monthly until month 12, and then patients will be evaluated every 3 months for a minimum of two years. A patient may be followed up to a maximum of five years depending on the date of enrollment; patients will be evaluated every 4 months after the first three years.

1. Medical history and physical examination including weight, major TB and HIV-related signs and symptoms and primary study outcomes (Months 7, 8, 9, 10, 11, 12, 15, 18, 21, 24, 27, 30, 33, 36 and *every 4 months thereafter or as clinically indicated*)
2. New diagnoses and/or hospitalizations since the last study visit (Months 7, 8, 9, 10, 11, 12, 15, 18, 21, 24, 27, 30, 33, 36 and *every 4 months thereafter or as clinically indicated*)
3. Posterior-anterior chest X-ray (Months 12, 18, and as clinically indicated)
4. Adverse experiences survey (Months 7, 8, 9, 10, 11, 12, 15, 18, 21, 24, 27, 30, 33, 36 and *every 4 months thereafter or as clinically indicated*)
5. Sputum semi-quantitative AFB smear and culture on solid media X 2 (Months 12 and 18, *or as clinically indicated*)
6. MTB drug susceptibility (as clinically indicated)
7. Urine or serum β-HCG pregnancy test (as clinically indicated)
8. CBC differential white blood cell and platelet count (Months 9, 12, 15, 18, 21, 24, 27, 30, 33, 36 and *every 4 months thereafter or as clinically indicated)*
9. Serum aspartate aminotransferase (AST), serum total bilirubin (as clinically indicated)
10. Serum Creatinine (as clinically indicated)
11. CD4+ lymphocyte counts (Months 9, 12, 15, 18, 21, 24, 27, 30, 33, 36 and *every 4 months thereafter or as clinically indicated*)
12. Plasma HIV RNA by Roche Amplicor (Months 9, 12, 18, 24, 30, 36, and then annually or as clinically indicated)
13. Quality of life assessment (months 12, 18, 24, 30 and 36)
14. Plasma storage (Months 9, 12, 18, 24, 30, 36 and *every 4 months thereafter, or as clinically indicated*)
15. PBMC storage/ immune function (Months 9 and 12, *or as clinically indicated*)
16. Store MTB isolate for DNA fingerprinting (All isolates from positive cultures of suspected relapse cases will be stored at -70° C for DNA finger printing with the patient’s initial isolate, as clinically indicated)
17. Disease specific diagnostic test (as clinically indicated)

## 7.4 Measurements at Time of and Post- Primary Study Outcome

### **7.4.1 Time of Event (Primary Study Outcome)**

When primary study outcome is achieved, this will be confirmed with the following (section 5.3, appendix I, Step 3):

1. Medical history and physical examination, including weight
2. Adverse Event Survey
3. New diagnosis and hospitalization survey
4. Pregnancy test (urine or serum)
5. CD4+ lymphocyte count
6. CBC with differential
7. Liver function tests (AST and serum bilirubin)
8. Serum creatinine
9. Disease specific diagnostic test

These results will be valid for a period of two weeks and may be used for the safety evaluation, if within the acceptable range and time frame (2 weeks).

While the primary outcome is being confirmed patients will also be undergoing a pre-ARV safety assessment. If the evaluations below were not previously within the acceptable range or outside the two week time frame the evaluation that was not acceptable will be repeated. This ARV safety assessment may include results from the following (see Appendix I, Step 3):

1. Medical history and physical examination, including weight
2. Adverse Event Survey
3. New diagnosis and hospitalization survey
4. Pregnancy test (urine or serum)*
5. Complete blood count with differential
6. Liver function tests (AST and serum bilirubin)
7. Serum creatinine

Patients with contraindications to antiretroviral therapy at the time of the primary outcome will be re-evaluated in one month or as clinically indicated to determine whether contraindication to therapy has resolved. Laboratory assessments and physical exams will be valid for 2 weeks from the time of collection: after 2 weeks, tests will be repeated to assess contraindications to therapy (see section 5.3).

### **7.4.2 Post- Primary Outcome - Antiretroviral Therapy Follow-up Visits**

Prior to being started on self-administered ARV therapy and entry to STEP 3, a pre-ARV baseline evaluation will be completed; this will include the following examination (appendix I, Step 3):

1. HIV RNA Roche Amplicor
2. Quality of life assessment
3. Plasma storage
4. PBMC storage/ immune function (Flow cytometry to assess cell surface activation markers, EliSpot to assess CD4 and CD8 T cell specificity to HIV and microbial peptides, Whole blood assays to determine antigen specific response to HIV and microbial antigens)

Assessments from confirmation of study outcome, or ARV safety evaluation may be used for pre-antiretroviral baseline if they were completed within two weeks before ARV baseline.

Patients will then be started on self-administered, ARV therapy and enter STEP 3 (Section 5.0 describing the initiation of therapy). Patients will receive detailed counseling about treatment with antiretroviral therapy. They will be informed that adherence measures will be made including unannounced home visits to record self-reported adherence and pill counts. Below is the schedule for STEP 3:

1. Medical history and physical examination including weight (Week 2, months 1, 2, 3, 6, 9, 12, 15, 18, 21, 24, 27, 30, 33, 36, and then every 4 months or as clinically indicated)
2. Adverse experiences survey (Week 2, months 1, 2, 3, 6, 9, 12, 15, 18, 21, 24, 27, 30, 33, 36, and then every 4 months or as clinically indicated)
3. New diagnoses and/or hospitalizations since the last study visit (Week 2, months 1, 2, 3, 6, 9, 12, 15, 18, 21, 24, 27, 30, 33, 36, and then every 4 months or as clinically indicated)
4. Urine or serum β-HCG pregnancy test (if suspect)
5. CD4+ lymphocyte count (months 3, 6, 12, 18, 24, 30, 36, then every 4 months thereafter or as clinically indicated)
6. CBC with differential and platelet count (months 3, 6, 12, 18, 24, 30, 36, then every 4 months thereafter or as clinically indicated)
7. Posterior-anterior chest X-ray (as clinically indicated)
8. Sputum semi-quantitative AFB smear and culture on solid media X 2 (as clinically indicated)
9. MTB drug susceptibility (as clinically indicated)
10. Serum aspartate aminotransferase (AST), serum bilirubin (months 1, 2, 6, 12, 18, 24, 30, 36, and every 4 months thereafter or as clinically indicated)
11. Serum creatinine (as clinically indicated)
12. Plasma HIV RNA level (months 1, 2, 3, 6, 12, 18, 24, 30, 36, and every 4 months thereafter or as clinically indicated)
13. Adherence Questionnaire (antiretroviral therapy) (months 1, 2, 3, 6, 9, 12, 15, 18, 21, 24, 27, 30, 33, 36, and then every 4 months or as clinically indicated)
14. Quality of life assessment (months 6, 12, 18, 24, 30, and 36)
15. Plasma storage (months 1, 2, 3, 6, 12, 18, 24, 30, 36, and every 4 months thereafter, or as clinically indicated)
16. PBMC storage/ immune function (month 3 and 6, or as clinically indicated)
17. HIV RNA genotyping (as clinically indicated)
18. Store MTB isolate for DNA fingerprinting (All isolates from positive cultures of suspected relapse cases will be stored at -70° C for DNA finger printing with the patient’s initial isolate, as clinically indicated)
19. Disease-specific evaluation per WHO staging system (as clinically indicated)

## 7.5 Sick Visit Evaluation

At any point during the study (Steps 1, 2, or 3), study patients will be encouraged to seek medical care for intercurrent illness or medical condition. At these sick visits, evaluation may as clinically indicated include the following:

1. Interim history and physical exam including vital signs, concomitant medications, adverse experiences, and major TB and HIV-related signs and symptoms
2. New diagnoses and/or hospitalizations since the last study visit
3. Sputum semi-quantitative AFB smear and culture on solid media X 2
4. Adverse experience survey
5. CBC, differential white blood cell and platelet count
6. Serum aspartate aminotransferase (AST), serum bilirubin
7. Urine or serum β-HCG pregnancy test (females of child bearing potential only)
8. Posterior-anterior chest X-ray
9. Plasma HIV RNA level
10. Serum creatinine
11. Adherence Questionnaire (TB or antiretroviral therapy)
12. Disease-specific diagnostic tests (Appendix II, WHO Staging Criteria
13. CD+4 lymphocyte count
14. Store MTB isolate for DNA fingerprinting (All isolates from positive cultures of suspected relapse cases will be stored at -70° C for DNA finger printing with the patient’s initial isolate)
15. Plasma storage
16. PBMC Storage

# 8.0 CLINICAL MANAGEMENT

## 8.1 HIV

### **8.1.1 Disease Management**

Management of HIV related complications and the prophylactic use of Septrin will be in line with a manual of Ugandan standard-of-care guidelines or the guidelines of the Ministry of Health, whichever is most recent.^64^ These guidelines provide recommended treatment and management of common fungal (oral and esophageal candidiasis, cryptococcal meningitis), viral (herpes simplex, HHV-8), and bacterial (pneumococcal pneumonia, sinusitis, etc.) complications of HIV. Because of high CD4+ cell count and the use of potent ARV, we will be prepared, but do not expect, to manage large numbers of opportunistic infections in this cohort during the Intervention Period. Laboratory facilities are available on-site for diagnosis of the most common infections including candida, cryptocococcal disease, bacterial infections, viral complications (HSV and VZV), and HIV-associated malignancies (Kaposi sarcoma, lymphoma, etc.). When women become pregnant during follow-up, they will be referred to the Uganda national Nevirapine implementation program for management of the pregnancy and delivery (Section 8.3)

### **8.1.2 Toxicity Management**

#### **8.1.2.1 General Guidelines for Adverse Experiences Associated with HIV and ARV**

#### **Therapy**

##### 8.1.2.1.1 Grades 1 and 2

Subjects who develop a Grade 1 or 2 adverse event or toxicity may continue study drugs without alteration of the dosage, except **as stated in the following sections**.

In general, for grade 1 toxicities, the patient should be followed carefully, and the study drug should be continued. The patient will receive specific counseling about the observed toxicity and will be instructed on what to do if symptoms worsen. For grade 2 toxicities, the patient should be followed carefully, with additional laboratory and/or clinic appointments as clinically indicated, and the study drug or combination held at the local clinician’s discretion until the event has resolved. The clinician should also evaluate concomitant medications to determine whether to withhold or discontinue the study drugs. Guidelines for the discontinuation of study drug is given in (Section 8.1.4.2).

Grade I and II events will be reported on standard case report forms.

##### 8.1.2.1.2 Grades 3 and 4

For any grade III or IV adverse event or toxicity, the offending agent(s) or all potential causative agents will be held pending evaluation. When clinically indicated, concomitant medications should be held first if the local clinician suspects they are contributing to the toxicity. In many instances, however, all study drugs will be discontinued at once to reduce risk to the patient. The on-site clinical coordinator will report the grade IV events to the study investigators and the Data and Statistical Center within 24 hours using standard procedures; the Data and Statistical Center will be responsible for reporting the grade IV event to the study sponsor. Grade III and IV adverse events and toxicities as defined by the Study Toxicity Tables (Appendices IV and V) and occurring during study therapy or within 60 days of the last study dose must be documented as Adverse Events. The standard reporting level for adverse events will be followed, reporting only grade IV events to the Division of AIDS.

Once a grade III or grade IV adverse event is suspected, the clinician(s) on-site should begin a diagnostic evaluation to determine the cause, or potential cause, of the observed event. If the grade III/IV adverse event is caused by hypersensitivity to the study drug, then the drug will be discontinued and appropriate substitutions made. If the adverse event (not a hypersensitivity reaction) has been caused by one or more of the study drugs, then offending agent(s) will be held until the adverse event has resolved to grade II or less. In general, for symptomatic grade III/IV events, the medication will be discontinued (see Section 8.1.4) and appropriate substitutions made (Section 5.2.1.4). For asymptomatic grade III/IV events, rechallenge with the study drug is allowed after resolution of laboratory values except for toxicities as outlined in the following section (8.1.2.2). If the investigator has compelling evidence that the adverse event has NOT been caused by the study drug, dosing may continue when the adverse event has resolved. If the event recurs within 4 weeks of rechallenging with the study drug, it must be discontinued.

#### **8.1.2.2 Antiretroviral Therapy Toxicity and Management**

Patients will be closely monitored for signs and symptoms of study drug toxicity. For all toxicities that require either temporary or permanent discontinuation of study therapy, relevant clinical and laboratory tests will be obtained as clinically indicated until final resolution or stabilization of the toxicity. Symptoms and laboratory findings will be graded by severity using the NIAIDS/ACTG Toxicity Tables (Appendices IV and V).

The study will use antiretroviral medications that have known and well-described toxicity profiles. The diagnosis and management of the major adverse events and toxicities are described below:

##### 8.1.2.2.1 Nausea (with or without vomiting)

Nausea following initiation of therapy with antiretroviral medications usually subsides or resolves during the first few weeks of treatment. With the onset of Grade >1 nausea, patients will be advised to take study drugs with food. With Grade >1 nausea, subjects may be treated as needed with antiemetics given orally or by suppository.

If the nausea is considered related to ZDV and is treatment limiting in the opinion of the site investigator, allowable drug substitution should be considered.

If nausea persists despite food and antiemetics or increases in severity, interrupt drug therapy. Once nausea resolves, therapy must be resumed using the full doses of study drugs, with the exception of the ZDV dose reduced to 200 mg BID. The dose will be increased as tolerated thereafter. If intolerable nausea recurs following resumption of therapy, protocol-allowable drug substitution(s) should be considered.

##### 8.1.2.2.2 Anemia/Neutropenia

Anemia and neutropenia are recognized toxicities of ZDV. In subjects with Grade 1 anemia, the study medications will be continued and the CBC monitored until resolution or until the anemia worsens. Subjects with Grade 2 anemia considered treatment limiting by the on-site investigators may have the ZDV dose reduced to 200 mg BID, to determine if the anemia or neutropenia resolves. If it does not resolve, protocol-allowable drug substitution(s) will be made. Modifications of ZDV will involve the use of a separate preparation instead of the fixed dose combination contained in Combivir.

Subjects with Grade >3 anemia or neutropenia attributed to ZDV may have treatment interrupted until the adverse experience has returned to Grade <2. Once the anemia or neutropenia has returned to Grade <2, ZDV may be restarted at a lower dose of 200 mg BID, to determine if the anemia or neutropenia recur. At the discretion of the site, the ZDV dose may be re-escalated as tolerated. If the toxicity does not resolve, then discontinue ZDV and substitute with suitable ARV.

Severe anemia will be managed with blood transfusions when the hemoglobin drops below 7 mg/dl.

Should a subject experience toxicity resulting from the fixed dose combinations of 3TC/ZDV, then the individual formulations of ZDV 300 mg tablet, or 3TC 150 mg tablet will be given separately with an appropriate dose reduction. Where necessary, protocol-allowed substitution(s) will be performed after discussions with the study investigators.

##### 8.1.2.2.3 Adverse Experiences to Abacavir (ABC)

ABC Hypersensitivity Reactions (HSRs) - *Background*

In clinical studies, approximately 3%-5% of patients receiving ABC develop an HSR that in rare cases has proved fatal. HSR is characterized by the appearance of symptoms indicating multiorgan/body system involvement. Symptoms usually appear within the first 6 weeks of starting treatment with ABC (median time to onset is 11 days), but may occur at any time while on therapy, and most often include fever, rash, gastrointestinal symptoms (nausea, vomiting, diarrhea, or abdominal pain), respiratory symptoms (dyspnea, sore throat, cough), and lethargy or malaise. Other signs and symptoms may include musculoskeletal symptoms (myalgia, rarely myolysis, arthralgia), headache, paresthesia, and edema. Respiratory tract symptoms (dyspnea, sore throat, cough) have been observed in approximately 20% of subjects who experience HSR.

The diagnosis of hypersensitivity to ABC remains a clinical diagnosis. There is no pathognomonic clinical sign or laboratory finding that renders the diagnosis. The key feature is that HSR is a multi-organ system reaction. Some patients with HSRs were initially thought to have respiratory tract disease (pneumonia, bronchitis, pharyngitis) or a flu-like illness. The multisystem nature of the HSR has led to misdiagnosis of the HSR as an intercurrent medical illness or as being related to another medication. HSR also has been unrecognized when it presents with less common symptoms or as a single symptom.

This misattribution of the symptoms of HSR to another medical condition or delay in diagnosis of hypersensitivity has resulted in ABC being continued or reintroduced, leading to more severe or rapid (within hours) onset of HSR or death. Therefore, the diagnosis of HSR should be carefully considered for patients presenting with symptoms of these diseases, even if another medical diagnosis seems likely. Renal failure and anaphylaxis have also been reported in association with HSRs. Reintroduction of ABC in subjects after treatment interruption, with no preceding symptoms of HSR, has, rarely, resulted in HSR. Given the risk profile of ABC, ABC therapy SHOULD NOT be restarted following an HSR, because more severe symptoms will occur within hours and may include life-threatening hypotension and death. Subjects who develop signs or symptoms of hypersensitivity should discontinue treatment as soon as an HSR is first suspected and should seek medical evaluation immediately, preferably on the same day that symptoms are reported.

Physical findings may include lymphadenopathy and, occasionally, mucous membrane lesions (conjunctivitis and/or mouth ulceration) and hypotension. The rash is variable and may be absent, but often appears maculopapular or urticarial. Laboratory abnormalities that may accompany ABC hypersensitivity include elevated liver function tests (LFTs), creatine kinase, or creatinine or lymphopenia.

Symptoms related to this HSR worsen with continued therapy and usually resolve upon discontinuation of ABC. Restarting ABC following an HSR results in a prompt return of symptoms within hours. This recurrence of the HSR may be more severe than on initial presentation and may include life-threatening hypotension and death.

ABC HSR - *Diagnosis of HSR*

The clinical diagnosis of suspected HSR is described in Section 6.3.1.2.1. Hypersensitivity Reaction associated with Abacavir.

*ABC HSR - Management of HSR*

If the clinical team makes the diagnosis of suspected HSR according to the standard definitions, then ABC will be held and discontinued when the diagnosis is confirmed by the resolution of symptoms. Patients with suspected HSR will be placed on the ‘ABC HSR Evaluation and Follow-up Protocol’. This protocol will involve complete initial diagnostic evaluation as described above, close follow-up with detailed evaluations by study physicians until all symptoms resolve or until an alternative diagnosis is established. Each patient placed on this protocol will be reviewed by experienced study clinicians and discussed with the senior study investigator at the point of patient contact. The information collected during this follow-up period will be recorded on a standard clinical report form for later review and classification.

In some cases of suspected HSR, the signs and symptoms consistent with ABC HSR may be mild or attributed to an intercurrent medical illness. In these cases, on-site physicians may decide to continue the ABC while monitoring signs and symptoms closely for any further development of ABC HSR. This decision will only be made after consulting senior investigators on the study at the point of patient contact. If signs or symptoms evolve, or HSR cannot be ruled out, ABC will be discontinued, even when other diagnoses are possible or likely (e.g., respiratory tract infections, gastroenteritis, or adverse events related to another drug).

In cases where the diagnosis of ABC HSR is strongly suspected, the patient will be instructed to hold all antiretroviral medications including ABC pending further evaluation. If signs and symptoms of ABC HSR resolve after discontinuing the medication, then the clinical diagnosis will be established, and ABC will be discontinued. The patient will be started on a protocol-allowable substitution regimen. The patient will be instructed to return unused ABC medication or combinations containing ABC to the clinic. The patient will be advised that they should never take ABC again, as life-threatening HSR may occur.

The clinical presentation and severity of the HSR will dictate the supportive care offered to patients with HSR. In the most severe cases, patients will be hospitalized for 24 hours on the Tuberculosis Ward 5/6 of Mulago hospital for observation. Supportive care will be given and will include intravenous fluids for hypotension, treatment of alternative diagnoses, antipyretics, and symptomatic and/or supportive treatment as indicated. Although there are no clinical data demonstrating the benefit of antihistamines or corticosteroids in the management of hypersensitivity, they will be used in severe cases. In mild or moderate cases, without unstable vital signs, the medication will be stopped and the patient observed in the clinic for several hours. These patients will be taken home by home visitors and seen in follow-up the following day.

All clinical data obtained will be reported on the relevant case report forms (CRF), and the event “ABC hypersensitivity” is to be reported as a serious adverse event on the Division of AIDS, Regulatory Control Center Safety Office, SAE form. In addition, the **GlaxoSmithKline’s** “Abacavir Hypersensitivity Reaction Adverse Event Record” should be completed and returned to **GlaxoSmithKline** per the instructions on the form.

*ABC HSR - Fever*

The onset of fever may herald hypersensitivity. If HSR is suspected, then patients will be placed on the HSR Evaluation and Follow-up Protocol. In the event of a clinical presentation consistent with hypersensitivity, ABC should be held until evaluated by a study doctor, as described above.

*Toxicity Management for ABC/3TC/ZDV Tablets*

Should a subject experience toxicity resulting from the fixed dose combinations of ABC/3TC/ZDV or 3TC/ZDV, administer the individual formulations of ZDV 300 mg tablet, 3TC 150 mg tablet and/or ABC 300 mg tablet simultaneously and dose reduce or make protocol-allowed substitution(s), if necessary with permission from the protocol chairs. If a subject experiences an ABC HSR, discontinue ABC permanently.

##### 8.1.2.2.4 CNS and Psychiatric Symptoms with Efavirenz (EFV)

Subjects will be informed that CNS symptoms may occur with EFV. These symptoms include serious psychiatric symptoms (e.g., depression) and CNS symptoms (e.g., dizziness, impaired concentration, insomnia, bad dreams, etc.). Patients will be informed that these symptoms are likely to improve with continued therapy. Dosing at bedtime improves the tolerability of these symptoms and is recommended during the first weeks of therapy and in subjects who continue to experience these symptoms. Subjects receiving EFV should be alerted to the potential for additive CNS effects when EFV is used concomitantly with alcohol or psychoactive drugs. For patients taking rifampin, dose reduction from 800-mg to 600-mg QD EFV is allowed if CNS symptoms persist.

Subjects also should be informed that EFV may cause dizziness, impaired concentration, and/or drowsiness and instructed that if they experience these symptoms they should avoid potentially hazardous tasks such as driving or operating machinery.

There have been reports of delusions and inappropriate behavior, predominantly in subjects with a history of mental illness or substance abuse. Severe acute depression (including suicidal ideation/attempts) has also been infrequently reported in both EFV-treated and control-treated subjects. Subjects who experience these symptoms should contact their doctor immediately because discontinuation of EFV may be required.

##### 8.1.2.2.5 Pregnancy with Efavirenz (EFV) or Abacavir (ABC)

If a subject becomes pregnant while on study, she must inform the study personnel and will be referred to appropriate antenatal clinic for the pregnancy. If she is on Trizivir, she may continue the medication for the duration of her pregnancy. If the patient is on EFV, she must discontinue it immediately and may replace it with nevirapine, if she chooses. Nevirapine cannot be used if the patient has CD4>250. For pregnant women with CD4>250, the antiretroviral regimen will be considered on a case-by-case basis by the study investigators.

##### 8.1.2.2.6 Rash with Efavirenz or Nevirapine

In the event that a subject experiences rash (with or without systemic symptoms), he/she should be evaluated for the possibility of an ABC or NVP HSR, for cutaneous adverse events associated with EFV or NVP. Subjects who experience rash during the first 2 weeks of NVP treatment must not increase the dose of NVP until the rash resolves.

For Grade 2 rash, antihistamines or topical corticosteroids may be prescribed. Subjects may continue all study drugs. The subject should be advised to contact the physician immediately if there is any worsening of the rash, or if systemic signs or symptoms develop that could be compatible with an HSR. It is extremely important to closely monitor the subject, especially if EFV or NVP has been interrupted because of rash, to ensure that symptoms do not worsen or that signs or symptoms consistent with clinical hepatitis or with hypersensitivity to ABC or NVP do not develop. Women appear to be at higher risk of NVP rash than men. All subjects developing a rash, at any time during NVP treatment, but particularly during the first 20 weeks, should have LFTs performed at that time. After the initial 20-week period, frequent clinical and laboratory monitoring should continue throughout NVP treatment.

Subjects developing signs or symptoms of severe skin reactions or HSR must be advised to discontinue NVP and seek medical attention immediately. Assess rash and evaluate ALT/AST. If severe rash OR rash with constitutional symptoms of organ dysfunction OR rash and increased ALT or AST occurs, then permanently discontinue NVP. If urticarial rash (mild or moderate) occurs, the subject may continue NVP, but DO NOT reintroduce NVP if it is stopped. For severe or generalized urticarial rash, NVP must be permanently discontinued.

EFV or NVP may be held at the investigator’s discretion until the cutaneous toxicity returns to Grade <1, at which time EFV therapy should be reinstituted at full dose. If NVP is held > 7 days, NVP should be reinstituted with the lead-in dose of 200 mg QD for 2 weeks, then 200 mg BID. If a Grade 2 cutaneous/allergic reaction recurs, the medical officer should discuss with the study investigators whether the subject should be further re-challenged. The subject should be closely monitored for recurrence of the rash, HSR, clinical hepatitis, or isolated changes in LFTs. If any of these conditions develop on NVP, the subject should immediately and permanently discontinue NVP. For subjects on NVP, if resolution takes more than 2 weeks, contact the study investigators.

If the rash is considered to be most likely due to concomitant illness or medication, standard management, including discontinuation of the likely causative agent, should be undertaken. If no other causative factor is found after clinical evaluation, the subject should be treated symptomatically until the rash resolves.

For Grade 3 rash, subjects should hold all study drugs. Upon resolution to Grade <1, the investigator should discuss at a study conference call with senior investigators whether re-challenge is appropriate. If the Grade 3 rash is considered to be most likely due to concomitant illness or medication, standard management, including discontinuation of the likely causative agent, should be undertaken.

For Grade 4 rash, (e.g., exfoliation, mucosal involvement, or target lesions [erythema multiforme]) or any evidence of Stevens-Johnson syndrome, subjects should discontinue all study drugs.

For grade ≥ 3 rash, do not substitute EFV for NVP or NVP for EFV

In the event that Grade 2 or 3 rash fails to resolve after 14 days, increases in severity, is associated with systemic (fever, malaise, nausea) or allergic (e.g., urticaria) symptoms, or is associated with mucosal or target lesions, all study drugs should be held until symptom resolution. NVP has been rarely associated with the development of severe rash, including SJS and toxic epidermal necrolysis (TEN). A new onset prodromal syndrome consisting of clinical symptoms manifested as fever (>39°C), malaise, cough, lymphadenopathy, edema, myalgia, and/or arthralgia may occur prior to the development of cutaneous manifestations of SJS, TEN, or the SJS/TEN overlap syndrome.

##### 8.1.2.2.7 Liver Toxicity and Nevirapine

Women with CD4+ cell counts >250 cells/µL, including pregnant women receiving chronic treatment for HIV infection, are at considerably higher risk (12-fold) of hepatotoxicity. Some of these events have been fatal. This subset of patients was identified by analyses of CD4 count at the time of initiation of NVP therapy. The greatest risk of severe and potentially fatal hepatitis events (often associated with rash) occurs in the first 6 weeks of NVP treatment. Men with CD4+ cell counts >400 cell µL at initiation of NVP therapy have a 3-fold higher risk of hepatoxicity compared to men with CD4+ cell counts ≤ 400 cells/µL. However, the risk continues after this time and patients should be monitored closely for the first 20 weeks of treatment with NVP. In some cases, hepatic injury progresses despite discontinuation of treatment. For grade ≥3 rash or hepatitis, do not substitute EFV for NVP.

Female subjects with CD4>250 and male subjects with CD4>400 should not be started on nevirapine. Subjects taking NVP should be monitored for the development of signs and symptoms of hepatitis, which include fatigue, malaise, anorexia, nausea, acholic stools, bilirubinuria, jaundice, liver tenderness, or hepatomegaly, with or without initially abnormal serum transaminase levels at weeks 2, 4 and monthly for the first 20 weeks from initiation. After the initial 20-week period, clinical and laboratory monitoring should continue every 2 to 3 months throughout NVP treatment. Subjects with these signs and symptoms must be advised to hold NVP and seek medical attention immediately and have LFTs performed. If the physician determines that the subject has clinical hepatitis with or without LFT abnormality, and there is any possibility NVP is the cause, NVP should be discontinued and not restarted after recovery.

##### 8.1.2.2.8 Immune Reconstitution Inflammatory Syndrome to TB (Paradoxical Reactions)

Any potent ARV regimen can produce transient worsening of clinical TB symptoms after initial response to antituberculous therapy. Up to one third of TB patients treated with potent ARV may develop immune reconstitution inflammatory syndrome (IRIS) to TB (paradoxical reactions).^52^ The most common manifestations include fever and worsening lymphadenopathy, but, occasionally, worsening pulmonary infiltrates is observed. Typically, the syndrome occurs 1-3 weeks after initiation of ARV and is self-limited. A brief course of corticosteroids is sometimes used to control exuberant reactions and will be permitted in the study. For further details see section 6.3.3 and Appendix VI.

### **8.1.3 Management of Virologic Failure**

Virologic failure is defined as confirmed HIV RNA > 200 copies/ml after six months of antiretroviral therapy and no evidence of non-adherence. Since the punctuated therapy lasts only six months, virologic failure will not necessitate a change in antiretroviral therapy in most instances. If, however, a subject reaches a primary study outcome during punctuated therapy, then an alternative regimen with minimal cross-drug resistance will be selected from available antiretroviral medications. For all patients on antiretroviral therapy, virologic failure will prompt a review of the clinical status of the patient using CD4+ count, physical examination, and laboratory test as indicated. If clinically indicated an alternative regimen with minimal cross-drug resistance will be selected from available antiretroviral medications. When virologic failure is diagnosed, patterns of drug resistance will be performed to guide future choice of treatment. If an effective antiretroviral regimen is not available, then antiretroviral medication will be discontinued.

For further details see Sections 5.2.1.4, 8.1.4.2.2, 10.2.2.3.

### **8.1.4 Discontinuation and Re-initiation of Antiretroviral Therapy**

#### **8.1.4.1 Definitions**

To hold study drug means to temporarily withhold the use of one or all medications. When a study drug is held, it may be re-started as clinically indicated.

To discontinue study drug means to stop one, or more, study medications without the intent of restarting the medication. It is a synonym for permanent discontinuation of treatment. A study medication may first be held during the evaluation of a patient before a decision is made to discontinue the medication.

To restart study drug means to provide again a study medication that was previously held in a patient.

#### **8.1.4.2 General Guidelines for Holding, Discontinuing, and Re-starting Study Antiretroviral Drug**

Certain events or conditions, described above, may require that study physicians either hold or discontinue study drugs. The decision to hold a patient’s antiretroviral medication during the study will be the responsibility of medical officers, their physician supervisors and study investigators. Decisions to discontinue or re-start study drug must be presented and discussed with the U.S. or on-site Principal Investigator, or their designee.

##### 8.1.4.2.1 Criteria for Holding Antiretroviral Therapy

At any time during treatment with antiretroviral therapy, antiretroviral medication may be held for conditions described in Section 8.1.2, or for any other adverse event that may be related to the use of medication. The medical officer may hold study medications at the time of an adverse event pending a complete evaluation of the event. The medical officer will notify local supervisors that the medication has been held and describe the reasons for the decision.

##### 8.1.4.2.2 Criteria for Discontinuing Antiretroviral therapy

At any time during treatment with antiretroviral therapy, antiretroviral medication may be discontinued for conditions described in Section 8.1.2, or for any other serious adverse event that may be related to the use of medication. In general, grade III and IV serious adverse events will result in discontinuation of one or more antiretroviral medications. For adverse events of grade I or II, antiretroviral therapy will be discontinued if the adverse event recurs when drug is re-started or when the adverse event worsens after dose adjustment.

Drug may be discontinued under the following circumstances:

- Allergic hypersensitivity reaction to study drug.
- Development of a toxicity that warrants permanent discontinuation of any study drug
- Recurrence of treatment-limiting toxicity on re-challenge will result in permanent discontinuation of the putative responsible drug.
- Confirmed virologic failure (HIV RNA > 200 copies/ml after six months of treatment) and no remaining alternatives for antiretroviral therapy (Section 8.1.3).
- Request by the subject to withdraw.
- The patient refuses further therapy.
- Failure or inability to comply with antiretroviral therapy
- Clinical reasons believed life threatening by study investigators, even if not addressed in the toxicity management of the protocol.
- Subject judged by the investigator to be at significant risk of failing to comply with the provisions of the protocol as to cause harm to self or seriously interfere with the validity of the study results.
- Termination of the study.
- Withdrawal of the study drug(s) at the request of the sponsor.
- Requirement for prohibited concomitant medications (see Table 8)

##### 8.1.4.2.3 Criteria for Re-starting Antiretroviral Therapy

The decision to restart antiretroviral therapy will depend on the type of adverse event and the study drug. Three general situations will arise:

1. If a patient develops intolerance to a medication as evidenced by an adverse event, then the medication(s) may be re-started once the adverse event has resolved or subsided to a grade II reaction or less. In this setting, the offending medication may be restarted under close supervision that involves direct observation of the initial dose and frequent clinical and laboratory monitoring thereafter.
2. If a patient develops an allergic or hypersensitivity reaction to one or more of the antiretroviral medications, the drug(s) must be discontinued and NOT be re-started. See comments below regarding ABC.
3. If a patient develops an adverse event to a medication other than an antiretroviral medication, then the antiretroviral medications may be re-started under careful clinical supervision.

Study medical officers and physicians have the option of substituting a protocol-approved antiretroviral medication when clinically indicated instead of re-starting medication.

*ABC – Re-Starting Therapy*

Although patients may develop allergic reactions to any medication, special rules about stopping and re-starting ABC are needed because of ABC HSR.

If the diagnosis of ABC HSR is made, then the ABC must be discontinued. If ABC was held for evaluation of an adverse event, and that adverse event is clearly ascribed to another cause, then medical officers may re-start ABC under direct observation of medical personnel and close follow-up. If ABC is to be re-started under these circumstances, the drug must be restarted by the site principal investigator or his physician designee after discussion of the risks and benefits of restarting the drug with the subject. Reintroduction of the drug must occur in Mulago Hospital where the subject can readily access medical care.

The on-site physician may deem that reintroduction of ABC is medically necessary in a subject who has a documented inter-current medical illness such as symptomatic hepatitis due to anti-tuberculosis therapy. The decision to restart therapy will be made by study investigators and co-investigators after a complete review of clinical information during a weekly conference call. The decision to restart ABC will be made in writing by the one of the study investigators (Drs. Diane Havlir, Christopher Whalen, Henry Boom, or Roy Mugerwa).

Before re-starting ABC, the study patient will be informed of the potential risk of HSR and instructed to hold the medication immediately if the symptoms recur and to report to the study clinic for evaluation and supportive treatment.

Subjects who have had an HSR must be advised that they should never take ABC (Ziagen®) again as a life-threatening second HSR can occur. Subjects should be advised to return all unused ABC at the time of discontinuation to the clinic.

## Tuberculosis

### **8.2.1 Tuberculosis Disease Management**

All patients will receive standard short-course antituberculous therapy recommended by the Centers for Disease Control and Prevention and the World Health Organization. This treatment includes isoniazid, rifampin, and pyrazinamide and ethambutol for two months followed by isoniazid and rifampin for four months (2RHZE/4RH, hereafter RHZE). Isoniazid will be given at a dosage of 300 mg per day. The daily doses of rifampin, ethambutol, and pyrazinamide will be adjusted according to body weight. For subjects weighing less than 50 kg, the daily doses will be rifampin 450 mg, pyrazinamide 1.5 gm, ethambutol 800 mg; for subjects weighing 50 kg or more, the daily doses will be rifampin 600 mg, pyrazinamide 2 gm, and ethambutol 1200 mg.

In Uganda, it has been estimated that 8% of MTB isolates from selected patients are resistant to isoniazid and 11% resistant to streptomycin. Multi-drug resistance, that is drug resistance to isoniazid and rifampin, is uncommon in Uganda at this time; in the course of evaluating over 500 TB cases in the past 5 years, we have identified only 3 cases of multi-drug resistant TB (MDR TB). All study patients will have drug susceptibility testing done at baseline so that results are available within two months. Participants with MDR TB will discontinue study participation and will be referred to the Tuberculosis Clinic where they will receive appropriate treatment for MDR TB and HIV infection. Appropriate treatment for MDR TB will be selected using drug susceptibility testing of isolates**.**

### **Tuberculosis-related Toxicity Management**

#### **8.2.2.1 General Guidelines - Grading and Management of TB-associated Adverse Events**

Adverse experiences will be graded on and ordinal scale from 1 to 4 according to standard toxicity tables (Appendix IV) after routine clinical evaluation. In general, the toxicities of anti-tuberculosis medications are well-documented (Section 8.2.2.2) and will be managed in a standard way. Grade 1 and 2 reactions are managed clinically without discontinuing treatment. If a grade 3 or 4 event is reported, the antituberculous medication most likely to produce that condition will be held until the Grade 3 or 4 adverse event resolves. The agent that was withheld may be restarted at a lower dose and the patient monitored closely for recurrence. If the symptoms recur, then the drug will be permanently discontinued and a second line antituberculous agent will be added.

#### **8.2.2.2 Toxicity due to Standard First Line** **Anti-tuberculosis Drugs Used in the Study**

The anti-tuberculosis drugs used in this study are standard first line drugs for the treatment of tuberculosis as recommended by the World Health Organization, the Centers for Disease Control in the U.S., and the American Thoracic Society. This section briefly reviews the major toxicities of each drug. Additional information is available in the Ugandan, and US national tuberculosis treatment guidelines, and Table 7 – Listing of Product Label Information for Adverse Experiences for Standard First Line Anti-TB Drugs.

The most common adverse effects of standard short course chemotherapy with INH, rifampicin, ethambutol and pyrazinamide are nausea, vomiting, fever, rash, and arthralgia, each occurring in less than 5% of TB patients receiving therapy. Hepatotoxicity, usually due to INH or rifampin, is a reversible, age-related event that is infrequent in patients younger than 35 years of age who do not consume excessive alcohol. Specific adverse events and toxicities are described in the sections that follow.

**Table 7**. Adverse reactions to first line anti-TB drugs

| **Drug** | **Common side effects** | **Uncommon Side Effects** | **Rare Side Effects** |
| --- | --- | --- | --- |
| Isoniazid (INH)  Rifampicin  Pyrazinamide  Ethambutol | anorexia  nausea  flushing | hepatitis  drug rash  peripheral neuropathy  hepatitis  drug rash  nausea/vomiting  thrombocytopenic purpura  febrile reactions  “flu-like” syndrome  hepatitis  vomiting  arthralgias  hyperuricemia  drug rash  retrobulbar optic neuritis  arthralgias | confusion  seizures  optic neuritis  hemolytic anemia  aplastic anemia  sideroblastic anemia  agranulocytosis  lupus-like reactions  arthralgias  gynecomastia  shortness of breath^a^  shock  hemolytic anemia^a^  acute renal failure^a^  gout  photosensitivity reactions  hepatitis  drug rash  hyperuricemia |

^a^More frequent with intermittent rifampicin administration than daily dosing.

*Isoniazid*

Isoniazid (INH), the hydrazide of isonicotininc acid, was introduced into TB treatment in 1952. Its antimicrobial activity is limited to mycobacteria where it inhibits the synthesis of mycolic acids, key components of the mycobacterial cell wall. INH is acetylated in the liver. Seventy-five to 95% is excreted as inactive metabolites in the urine. The incidence of adverse reactions to INH is approximately 5%. Rash (2%), fever (1.2%), jaundice (0.6%), and peripheral neuropathy (0.2-2%) are the most frequent adverse reactions^66^. Rare adverse events include neutropenia, thrombocytopenia, seizures, encephalopathy, anti-nuclear antigen (ANA) positive small vessel vasculitis, dyspepsia, red cell aplasia, optic neuritis, methemoglobinemia, urinary retention and pellagra^66^. Severe and rarely fatal hepatitis may occur during INH treatment. The risk of hepatitis increases with age. In an earlier USPHS study, 1.3% of 12, 828 persons receiving INH preventive therapy were reported to have drug-related hepatitis^67^. No cases of INH-hepatitis occurred in persons less than 20 years old compared to 0.3% of those aged 20-34 years, 0.12% of those aged 35-49 years and 0.23% of those between 50 and 54 years. Eight (0.06%) fatalities occurred in this study. More recently, significant hepatotoxic reactions occurred in only 0.1% of over 11,00 consecutive individuals receiving clinically monitored INH preventive therapy at a U.S. public health clinic^68^.

Elevations of serum aminotransferases occur in 10-20% of all patients receiving INH. These increases developing during the initial weeks of therapy are generally asymptomatic and usually resolve despite continuation of the drug. Persons who consume ethanol (especially on a daily basis), those with pre-existing liver disease, and possibly pregnant and post‑partum women are at increased risk for hepatotoxicity. INH hepatotoxicity usually is reversible if the medication is stopped when initial symptoms occur.

In the event of INH hepatotoxicity (Grade III or greater), INH will be withdrawn until the hepatitis resolves when it will be reintroduced at a lower dose and escalated as tolerated. If hepatotoxicity recurs, substitution with a fluoroquinolone (Levofloxacin) will be made if the toxicity occurs during the first two months of treatment; thereafter ethambutol will be substituted.

INH therapy also is associated with the development of a dose-dependent peripheral sensory-motor neuropathy via interactions with pyridoxine and pyridoxal-5-phosphate metabolism. Peripheral neuropathy is more frequent in patients who are malnourished, have diabetes mellitus, chronic renal failure, or alcoholism, and pregnant women. INH peripheral neuropathy can be prevented or reversed in nearly all cases by supplemental oral pyridoxine 25-50 mg per day.

*Rifampicin*

Rifampicin is a first line bactericidal agent which inhibits DNA-dependent RNA polymerase. Rifampicin is well‑absorbed with good penetration of inflamed meninges. Usual doses are 10 mg/kg/d (up to 600 mg/d). Rifampicin is a potent hepatic enzyme inducer and has many clinically significant drug interactions affecting the dosing of other medications such as coumadin, theophylline, and HIV protease inhibitors. Rifampicin increases the metabolism of estrogens and can result in decreased efficacy of hormonal contraceptive and breakthrough bleeding. The principal side effects are GI intolerance (1.5%), skin rash (0.8%), fever (0.5%), cholestatic jaundice and hepatitis (1.4%)^66,69^. A flu-like syndrome characterized by malaise, fever, chills, headache and myalgias can occur and is more frequent with intermittent dosing. Hemolytic anemia, thrombocytopenia (0.08%) and purpura, and acute renal failure are rare complications requiring permanent discontinuation of the drug^66,69^. These adverse events also are more frequent with intermittent dosing. Body secretions including urine, sweat and tears are discolored orange or orange-red during rifampicin administration.

*Ethambutol*

Ethambutol is primarily a mycobacteriostatic agent given to prevent emergence of drug resistance to other first line drugs. It has low CSF penetration. MICs against *M.* tuberculosis usually range from 1-5 µg/ml. Usual dosages are 15 to 25 mg/kg/d. The drug is renally excreted and the dosage should be decreased in patients with renal insufficiency. Adverse events occur in less than 2% of patients receiving ethambutol at the 15 mg/kg dose and include decreased visual acuity (0.8%), rash (0.5%) and asymptomatic hyperuricemia^66^. The most important side effect of ethambutol is retrobulbar neuritis that presents as blurred vision or decreased peripheral and color vision. The incidence of ocular toxicity is low at the 15 mg/kg/d dose^69^. Patients receiving ethambutol should be instructed about symptoms of ocular toxicity. Visual examination with Snellen charts will be performed whenever a patient receives ethambutol. If ocular toxicity is suspected, ethambutol will be discontinued. Ocular toxicity is reversible in most cases after the discontinuation of the drug.

*Pyrazinamide*

Pyrazinamide is an important first line bactericidal agent first used in TB treatment in the 1950s. Pyrazinamide is highly active in the acidic intracellular environment against tubercle bacilli in macrophages. It has good tissue penetration including the CSF. Usual doses are 15-30 mg/kg/d, up to 2 gm/d. The most frequent side effects are GI intolerance, hepatotoxicity (1.3%), arthralgias (1-7%), hyperuricemia due to blockade of urate excretion up to 66%), and rarely acute gouty arthritis^66,69^. Asymptomatic elevations in serum uric acid are frequent, usually occur during the first or second month of treatment, and are self-limited and require no specific treatment^70^. Minor arthralgias also may occur during pyrazinamide treatment and can usually be treated with salicylates or non‑steroidal inflammatory agents such as indomethacin while continuing the drug. Pyrazinamide does not further increase the risk of hepatotoxicity when used in combination with INH and rifampicin^70-72^.

### **8.2.3 General Guidelines for Holding, Discontinuing, and Re-starting Antituberculosis Treatment**

Certain events or conditions, described above, may require that study physicians either hold or discontinue anti-TB drugs. The decision to hold a patient’s antituberculosis treatment during the study will be the responsibility of medical officers, their physician supervisors and study investigators. Decisions to modify the treatment regimen must be presented and discussed with the U.S. or on-site Principal Investigator, or their designee.

#### **8.2.3.1 Criteria for Holding Antituberculosis Therapy**

At any time during treatment of TB, medication may be held for conditions described in Section 8.2, or for any other adverse event that may be related to the use of medication. The medical officer may decide to hold one or more of the prescribed medications at the time of an adverse event pending a complete evaluation of that event. The medical officer will notify local supervisors that the medication has been held and describe the reasons for the decision.

#### **8.2.3.2 Criteria for Discontinuing Antituberculosis Treatment**

In general, grade III and IV serious adverse events attributable to antituberculosis medication will result in discontinuation of one or more antituberculosis medications. For adverse events of grade I or II, antituberculosis therapy will be discontinued if the adverse event recurs when drug is re-started or when the adverse event worsens after dose adjustment.

Drug may be discontinued under the following circumstances:

- Allergic hypersensitivity reaction to study drug.
- Development of a toxicity that warrants permanent discontinuation of any study drug
- Recurrence of treatment-limiting toxicity on rechallenge will result in permanent discontinuation of the putative responsible drug.
- Request by the subject to withdraw.
- The patient refuses further therapy.
- Clinical reasons believed life threatening by study investigators, even if not addressed in the toxicity management of the protocol.
- Subject judged by the investigator to be at significant risk of failing to comply with the provisions of the protocol as to cause harm to self or seriously interfere with the validity of the study results.
- Termination of the study.

#### **8.2.3.3 Criteria for Re-starting Antituberculosis Therapy**

The decision to restart antituberculosis treatment will depend on the type of adverse event and the anti-TB drug. Three general situations will arise:

1. If a patient develops intolerance to a medication as evidenced by an adverse event, then the medication(s) may be re-started at a modified dose, once the adverse event has resolved or subsided to a grade II reaction or less. In this setting, the medication may be restarted under close supervision that involves direct observation of the initial dose and frequent clinical and laboratory monitoring thereafter.
2. If a patient develops an allergic or hypersensitivity reaction to one or more of the antituberculosis medications, the drug must NOT be re-started.
3. If a patient develops an adverse event to a medication other than an antituberculosis medication, then the antituberculosis medications may be re-started under careful clinical supervision.

Study medical officers and physicians have the option of substituting a second line antituberculosis medication when clinically indicated instead of re-starting medication.

## 8.3 Management of Pregnancy

At the time of recruitment, pregnant women will be excluded from the study. During screening, women will be advised in the informed consent regarding possible teratogenic side effects (observed in primates and humans) of efavirenz.

Women who will be receiving ARV therapy must use two effective contraceptive methods, including one barrier method, while receiving protocol-specified medications and for 4-weeks after stopping the medications. The choice of methods may include: condoms (male or female) with or without a spermicidal agent; diaphragms or cervical cap with spermicide; IUD; and hormonal-based contraception. If the female patient is intolerant to ABC and will be receiving efavirenz, she will first have a pregnancy test to rule out pregnancy within 48 hours of starting EFV and counseled that she must use two effective contraceptive methods, including a barrier method, while receiving an efavirenz-containing regimen and for 6 weeks after stopping the medications to be eligible to participate in the study. If the female study patient is not of reproductive potential, the use of contraception is not required for eligibility. Written documentation confirming the use of two effective contraceptive methods or laboratory measurement of FSH in women without reproductive potential will be provided in those female subjects receiving efavirenz.

Once enrolled into the study, the women participants on antiretroviral therapy will be screened monthly for pregnancy during the intervention period; after the intervention period, pregnancy screening will be performed as indicated thereafter. For women who become pregnant while on antiretroviral therapy, Trizivir may be continued for the duration of the pregnancy, labor, and delivery. If the woman is taking EFV during the intervention or post-outcome period, then NVP will be substituted for EFV for women with CD4≤250. Nevirapine cannot be substituted if the female subject has CD4>250. For pregnant women with CD4>250, the antiretroviral regimen will be considered on a case-by-case basis by the study investigators. Women who choose to breastfeed postpartum, will continue on the NVP regimen while they breastfeed.

All women initiated on NVP will be monitored for hepatitis (see Section 8.1.2.2.5). For women who choose not to breastfeed or who have finished breastfeeding, NVP will be discontinued and either ABC or EFV will be substituted according to protocol guidelines.

For women who become pregnant and are not taking antiretroviral therapy at the time of pregnancy, NVP will be given at the time of labor and delivery, as is currently the standard of care in Uganda, through specialized antenatal clinics at Mulago Hospital. If the standard of care in Uganda to prevent mother-to child transmission of HIV changes during the course of the study, women will be offered this treatment.

All women will be referred to the ante-natal clinics of Mulago Hospital for management of their pregnancy, delivery, and peri-partum period. These clinics will counsel women about the risks and benefits of breastfeeding their children. These clinics will also refer children to clinics that handle pediatric HIV infection.

Although not an adverse event, pregnancy will be a reportable event. The study will keep complete records on each pregnancy and its outcome.

Because the study protocol excludes pregnant women at enrollment, and because the duration of punctuated therapy is only 6 months, it is unlikely that women will deliver while receiving punctuated therapy. If in the unlikely event this does occur, punctuated therapy would be continued as described above, unless there was immediate risk to mother or child.

## 8.4 Concomitant Medications

### **8.4.1 Permitted Medications**

The following medications or classes of medications will be permitted during the study when a patient is or is not receiving ARV therapy. The indication, dose, frequency, and duration of use will be recorded on a case report form.

Topical and oral antifungal agents are permitted, except for oral ketoconazole and itraconazole.

Treatment, maintenance, or chemoprophylaxis with approved agents for opportunistic infections as clinically indicated.

All antibiotics as clinically indicated (unless excluded by Section 8.4.2). Macrolide antibiotics other than clarithromycin should be used when possible. Systemic corticosteroid use for <21 days is permitted as medically indicated; chronic systemic corticosteroid use is not permitted, unless it is within physiologic replacement levels.

Regularly prescribed medications such as antipyretics, analgesics, allergy medications, antidepressants, sleep medications, oral contraceptives, megestrol acetate, pyridoxine, or any other medications are permitted as medically indicated (unless excluded in Section 8.4.2). Due to potential interactions between study medications and oral contraceptives or depoprogesterone, oral contraceptives or depoprogesterone cannot be used as the sole form of birth control.

Alternative therapies such as vitamins, acupuncture, and visualization techniques will be permitted. Herbal medications should be avoided. Subjects should report the use of these therapies; alternative therapies will be recorded but not keyed.

Although not prohibited, alcohol should be consumed with caution as it may exacerbate CNS symptoms of EFV.

Vaccinations, except any HIV vaccine, are permitted but should not be administered within 14 days before an HIV-1 RNA viral load evaluation.

### **8.4.2 Prohibited Medications**

The drugs listed in Table 8, may not be used concomitantly with the antiretroviral medication. Many of these medications are not routinely available in Uganda. During the course of the study, however, patients may gain access to some or all of these medications. With the exception of HIV vaccines, investigational agents are not allowed at any time during the study.

**Table 8**. Prohibited Concomitant Medications

| Agent by Class | Prohibited Concomitant Agents |
| --- | --- |
|  |  |
| Alternative/Complementary | St. John’s wort (*Hypericum perforatum*) |
| Analgesics | Propoxyphene |
| Antiarrhythmics | Amiodarone  Bepridil |
|  | Quinidine |
| Antihistaminics | (  Astemizole  Terfenadine |
| GI Motility | Cisapride |
| Sedative/hypnotics | Midazolam  Triazolam |
| Anti-infectives | Ketoconazole  Itraconazole |
| Other | Ergots including Dihydroergotamine, Ergonovine, Ergotamine, and Methylergonovine (Methergine™) |
|  | Thalidomide  Doxorubicin  ribavirin |

### **8.4.3 Precautionary Drugs**

Package inserts of concomitant agents should be referred to whenever a concomitant medication is initiated or a dose is changed to avoid drug interaction adverse events. Use of the agents below while on study drugs may require additional monitoring for adverse events. These precautionary medications include, but may not be limited to:

| Agent by Class | Precautionary Concomitant Medications |
| --- | --- |
|  |  |
| Analgesics | Opioids, including: |
|  | Codeine |
|  | Methadone |
|  | Morphine |
|  | Pentazocine |
| Anesthetics | Propofol |
| Antiarrhythmics | Disopyramide |
|  | Encainide |
|  | Lidocaine |
|  | Mexiletine |

| Anticonvulsants | Carbamazepine |
| --- | --- |
|  | Lamotrigine |
|  | Phenobarbital |
|  | Phenytoin |
|  | Valproic acid |
| Antihistaminics | Chlorpheniramine |
|  | Diphenhydramine |
| Anti-infectives | Clarithromycin |
|  | Dapsone |
|  | Erythromycin |
|  | Fluconazole |
|  |  |
|  |
| Beta blockers (selected agents listed, caution should be exercised for the entire class) | Atenolol  Metoprolol  Propranolol |
| Calcium channel blockers (selected agents listed, caution should be exercised for the entire class) | Diltiazem  Nefidipine  Verapamil |
| Hormonal agents | Estrogens and Progesterones |
|  | Glucocorticoids |
| Hypoglycemics | Pioglitazone |
|  | Clofibrate |
|  | Fenofibrate |
|  | Fluvastatin |
| Psychiatric medications | Bupropion |
|  | Chlorpromazine |
| Psychiatric medications | Clozapine |
|  | Fluoxetine |
|  | Nefazadone |
|  | Paroxetine |
|  | Risperidone |
|  | Venlafexine |
| Tricyclic Antidepressants (including but not limited to) | Desipramine  Imipramine  Nortriptyline |
| Sedative/Hypnotics | All benzodiazepines |
|  | Alprazolam |
|  | Clorazepate |
|  | Diazepam |
|  | Estazolam |
|  | Flurazepam |
|  | Oxazepam |
|  | Temazepam |

|  | Busprione |
| --- | --- |
|  | Zaleplon |
|  | Zolpidem |
| Other agents | Chlorzoxazone |
|  | Cimetidine |
|  | Naloxone |
|  | Promethazine |
|  | Sildenafil |
|  | T3-thyroid hormone |
|  | Warfarin |

## 8.5 Abnormal Laboratory Values

The investigator or designee will review all abnormal laboratory values (baseline or occurring during the course of a subject’s participation in the study) with regard to clinical significance and etiology (related or not related to study medication) with 24-hours of receipt of results. Laboratory abnormalities will be reported as Serious Adverse Experiences if they meet the criteria for SAE (sections 6.3.1.1).

## 8.6 Supervision of Clinical Management

Administration and monitoring for ARV efficacy and toxicity will be supervised by a U.S. trained internist with knowledge and experience in the care of HIV-infected patients and in the use of antiretroviral therapy. Before the study begins, Ugandan medical officers will have an intense initial training period on ARV therapy, directed by the study principal investigators. An on-site physician will be present for the protocol initiation to supervise the medical officers. There will be continued education for the medical officers in ARV therapy and a weekly case conference with the medical officers to review progress, medication toxicities and all protocol issues. A U.S. physician will be available for extended time periods of subsequent years of the protocol to work on site in Uganda. The study investigators will make regular site visits to assess knowledge and practice among study personnel. Dr. Havlir and colleagues will oversee the ARV therapy portion of the study and will be available by email and telephone to answer all protocol related questions.

# 9.0 DATA INTEGRITY

## 9.1 Organizational Structure

The study team will be organized in three centers: a Clinical Center, a Study Coordinating Center, and a Data and Statistical Center. The Clinical Center will be located in Kampala, Uganda, at the Tuberculosis Research Clinic of Old Mulago Hospital. The main responsibilities of this Center will be screening and recruitment of study patients, provision of medical care, data collection and storage, on-site, quality assurance and control and on-site monitoring. The Study Coordinating Center will be located at CWRU in the Tuberculosis Research Unit. The main responsibilities of this center will be to coordinate study sites, protocol implementation, review of clinical events (e.g., severe adverse events, study outcomes, etc.), weekly conference calls, shipping and clearing of materials and supplies. The Data Center will be located at the San Francisco General Hospital Campus at University at California, San Francisco. We include this center because this trial will be the first antiretroviral study performed by the Uganda-CWRU Research Collaboration and represents a major new direction of research in Uganda. To ensure optimal performance of the study and safety to the participants, this Data Center, with its expertise in ARV treatment and experience in performing clinical trials of new ARV therapies, will monitor for safety and toxicity, provide quality control/assurance for data collection, and perform interim and final analyses. Prior to study implementation a manual of operations containing standard operating procedures, clinical data collection forms, protocol amendments, communications will be developed by study investigators and staff.

## 9.2 Data Collection

Clinical data will be recorded in the patient’s medical record. Copies of laboratory results will also be placed in the medical record. The medical record will represent the source document for the study. Subjects will be identified on the study forms by their study identification number and initials; full names will not be used on study forms. A dedicated data manager will provide surveillance and reporting of collected data to investigators on a routine basis.

## 9.3 Data Management

Health care providers are given in-service training by investigators about forms and study procedures before the start of the study and periodically throughout the study.

The data center will use Teleform Elite software (Cardiff Software, San Marcos California) which provides automated data entry from remote sites. Briefly, data collection forms are developed and formatted to interface with the Teleform software by study data managers. After a patient encounter, one copy of the data form is placed in the clinical chart and the other form is scanned into multi-page image files and sent to the UCSF data center. These files are compressed and stored. The data files are then read into the Teleform program, which aligns the form and records the data according to a predefined electronic template and then transferred into in a database management system.

Once in the electronic database, the data are edited and cleaned using standard programs to flag missing data and out-of-range values. Formal queries will be generated from the data center and sent to Uganda where the data managers will be responsible for responding to each query. The electronic data is backed-up on a regular basis. Complete copies of datasets will be maintained both in Uganda and at UCSF Data Center. In the U.S. Data Management Center, the data will be archived on a mainframe computer and stored in a fireproof box. This system has built-in quality assurance that begins on-site at the time of data collection and continues until the final information is validated in the final database. (Appendix VII) The hard copy data are stored in the patients’ medical records.

The specimens collected for storage are accessioned and stored either in the Joint Clinical Research Center laboratory or in the Makerere University-JHU-CWRU Research Collaboration Laboratory. The samples are entered into a database and assigned a position in one of the –70°C freezers. This system allows for the ready retrieval of stored samples for later analysis. These freezers are connected to voltage stabilizers and are backed up by generators in the event of power outage.

## 9.4 Data Quality Assurance/ Quality Control

There is currently a data quality management plan in place in Uganda that has been used as part of past and ongoing IND clinical trials. This plan has been acceptable to the Division of Microbiology and Infectious Diseases and the Food and Drug Administration in the U.S. The plan assesses quality control through multiple levels of checks on data collection, entry, and storage (Appendix VII). The plan also contains regular and systematic quality assurance through the review of clinical records.

Data will be checked to insure appropriate documentation of entry criteria, accurate transfer of information from progress notes to case report form and accurate entry into the database. The goals of our quality control program will be to ensure: a) documentation of informed consent, b) completion of the case report forms, c) accurate data in the case report forms, and d) resolution of inconsistencies in the database. Results of the quality control program will be recorded on a comprehensive quality assurance form (Appendix VII).

We expect to audit all case report forms initially, and then at least 50% of forms after staff experience and accuracy is evident. Forms will be electronically transmitted within 24 hours of being seen in the clinic. Feedback will be provided to appropriate staff on a quarterly or more frequent basis, as needed.

# 10.0 STATISTICAL CONSIDERATIONS

## 10.1 Sample Size

In this randomized clinical trial we will enroll a total sample size of 350 HIV-infected TB patients into Step 2 with 175 randomized to punctuated therapy and 175 randomized to anti-TB treatment only and delayed ARV therapy. The sample size was estimated to provide a power of 80 – 90% to detect an effect size of 35% or greater for HIV disease progression. This effect size translates into an average delay of about one year before the need to start ARV therapy. The sample size will not include patients who enter Step 1 but are not eligible to progress to Step 2.

**Table 9. CD4+ cell decline before and after TB in Ugandan adults**

| Study Group | N | Rate of change in CD4+ |
| --- | --- | --- |
| Observational Cohort | 230 | One year: -83 cells/μL  Two Years: -162 cells/ μL |
| Incident TB cases | 15 | Before TB: -11.9 cells/month  After TB: -8 cells/month |
| TB Cases after treatment | 95 | Mean decline: -8.7 cells/month |

The rate of decline in CD4+ cell count has been estimated in two different groups of TB patients in Uganda (Table 9). Based on these findings, we estimate a decline of between 7 to 10 cells/month in the reference group. We therefore expect that the CD4+ count will decline at a similar rate in the TB patients receiving anti-TB treatment only and possibly delayed ARV therapy.

Effect size (ES) was used to determine power along a range of sample sizes. ES is defined as: $ES=\left(1-\frac{\lambda_{e}}{\lambda_{r}}\right)\times100$, where λ_e_ and λ_r_ are the hazard rates for disease progression for the exposed and reference groups, respectively.^73^ In this trial, the exposed group will receive punctuated therapy and the reference group will receive anti-TB treatment only with possible delayed ARV therapy. The ratio of hazard rates, λ_e_/λ_r_, is the relative hazard and is an estimate of the relative risk for disease progression in the exposed compared with the reference group. In this analysis, we used the Cox-Mantel test to simulate power for samples sizes from 0 to 300 subjects per arm (Figure 5A and 5B) under two scenarios (see figure legend for assumptions). In one scenario, the rate of disease progression in the reference group was 50% at two years (Figure 5A), and in the other, the rate was 50% at 3 years (Figure 5B).

**Figure 5. Sample Size Calculation**

Power of designed study according to sample size per arm and effect size. Assumptions in calculations: alpha=0.05 (2 tailed), 1600 simulations, 95% confidence intervals for estimated proportions, a 3-year accrual period, and 5% dropout per year, constant hazard throughout period, and Cox Mantel-Haenszel test statistic for the comparison of two survival distributions.^74^ (A.) 50% disease progression by 2 years in reference group; (B.) 50% disease progression by 3 years in reference group, i.e., slower disease progression than expected. The simulations were implemented using N QueryAdvisor.^75^

Under the assumption that 50% of patients in the reference group will progress to CD4+ count < 250/µL within 2 years and an effect size of 35%, a power of 80% would be achieved with 125 TB patients per arm, and 90% with 175 patients per arm. Under the assumption that 50% of patients in the reference group will progress to CD4+ count < 250 within 3 years and an effect size of 35%, a power of 80% would be achieved with 175 subject per arm, and 90% with 250 subjects per arm. Based on this analysis, we chose a sample size of 175 TB patients per arm for a total of 350 TB patients. The sample size of 350 TB patients also ensures a large enough sample size to examine secondary outcomes with power of 80% or greater in each comparison.

Figure 5.

## 10.2 Analytic Strategy

### **10.2.1 Descriptive Analysis**

A descriptive analysis will examine the distribution of continuous variables and the frequency histograms for categorical variables. In non-normal distributions, such as viral load, transformations will be used to normalize the data for statistical analysis. To assess the adequacy of randomization, baseline characteristics of the study groups will be compared using Wilcoxon rank sum test (students t-test for normal distributions) for continuous data or chi-square test for categorical data.^76^ Where the expected frequencies are small, exact methods may be used. Cumulative incidence of events (e.g., CD4 < 250, relapse TB) will be estimated as the number of events per population at risk at baseline for each treatment group; 95% confidence intervals will be determined using standard methods.^77^ Incidence density will be estimated as the number of new, or incident, events per 100 person-years of observation; appropriate 95% confidence intervals can be calculated.^77^ The clinical course of HIV infection will be characterized as the incidence density of opportunistic infections following the diagnosis of active TB. The relative risk of opportunistic infections in the treatment versus the reference groups will be estimated with 95% confidence intervals.^77^ Statistical analysis will be performed using SAS and S-Plus statistical packages.

### **10.2.2 Analytic Strategy by Specific Aim**

#### **10.2.2.1 Specific Aim 1**

To determine whether a punctuated six-month course of ARV therapy during treatment of active TB will delay HIV disease progression, as measured by time until CD4+ count falls below 250/ml, the rate of decline in CD4+ cell counts and the incidence of an AIDS-defining condition (Appendix II, WHO stage IV HIV infection or a definitive diagnosis of a severe bacterial infection that meets WHO Stage 3 classification ), among HIV-infected persons with active TB and CD4+ cell count above 350 cells/ml.

The analysis of this aim will be done in two stages. First, in an analysis of the randomized clinical trial, we will compare the rate of HIV disease progression, as measured by time until CD4+ count falls below 250/ml and the rate of decline in CD4+ cell counts, between TB patients randomized to receive either punctuated or anti-TB medications only with possible delayed ARV therapy using an intention-to-treat approach. All subjects randomized in the study will be analyzed according to the group to which they were assigned regardless of whether they remained adherent to the ARV treatment. This as-assigned analysis will fully preserve the benefits of randomized treatment allocation and will provide a conservative estimate of the benefit of the intervention. It represents the main analysis of the trial.

HIV Disease Progression when CD4+ count < 250/ml

The primary outcome for the study is HIV disease progression. This is defined as the time interval between enrollment and the development of one or more of the following endpoints, whichever occurs first: decline of CD4+ count < 250/µL, new AIDS-defining condition, or death. When a subject has met the criteria for primary outcome for CD4+ counts, the date for the first of 2 consecutive CD4+ counts will be used to mark failure time. The primary analysis of the study for each outcome will be to compare the failure-time distribution of the treated to the reference group. Separate failure-time distributions will be estimated for each treatment group using the Kaplan-Meier method^78^ and compared using the log-rank test.^79^ Stratified analysis will be performed for the primary outcome according to categories of baseline viral load.^80^ A Cox proportional hazards regression model^81,82^ will be developed to evaluate the effect of treatment on disease progression. In these analyses, the failure event will be HIV disease progression; subjects will be censored because of withdrawal during the study or at end of study. We will assess whether censoring is independent of study outcome as this is a fundamental assumption of survival analysis. In these regression models, observed event-free survival will be the dependent variable.

The basic Cox proportional hazards model for this analysis will be formulated as follows:

where h (t,X) is the observed hazard, h_0_ (t) is the baseline, unmeasured hazard for disease progression, β is the regression coefficient for treatment, ARV is the variable coding for immediate versus delayed ARV treatment (1 and 0, respectively), γ_i_ the vector of regression coefficients for the covariates, X_i_ is the vector of covariates that may include time since treatment. This vector will contain known prognostic factors for HIV infection in Africa such as age, tuberculin skin test, baseline CD4+ count, hemoglobin, and performance status. This vector of covariates will also include the time period between initiating antituberculosis therapy and date of randomization to account for varying times in initiating antiretroviral therapy. The assumption of proportional hazards will be tested using graphical methods and formal statistical tests. If this assumption does not hold, then stratified Cox regression models will be developed, stratifying on the variable with non-proportional hazards.

The hazard ratio, or relative risk, for the comparison of immediate treatment versus delayed treatment will be estimated from the Cox regression model as:

where ARV (1) codes for immediate treatment and ARV (0) for delayed treatment. The likelihood ratio test using the –2 log-likelihood for nested models will be used to assess statistical significance. Confidence intervals (95%) for the hazard ratio will be calculated using the standard error of the regression coefficient.

In any clinical trial, non-adherence with the assigned treatment poses challenges not only in the conduct of the trial but in the analysis. Adherence with study medication will be optimized through health education, frequent monitoring and modified directly observed therapy during punctuated therapy. Despite these efforts, it is possible that non-adherence will occur. Non-adherent participants will remain in the analysis to preserve the strict intention-to-treat approach. To evaluate the potential impact of non-adherence, we will perform a stratified analysis for main outcomes according to level of non-adherence, as we have done in previous clinical trials.^83,84^

Although random allocation of treatment would balance known and unknown confounding factors at baseline between the punctuated ARV versus the reference arm, it is possible that losses during the first 6 months of observation will alter the nature or the study groups at the beginning of this observation period. To address this potential problem, the prognostic characteristics of the intervention and reference groups will be compared at the start of the post-intervention observation period. Failure-time methods that utilize the area under the survival curve or marginal structural models will be developed to adjust for any differences between these groups after the intervention period.

Rate of change in CD4+ count according to treatment periods

In addition to the main outcome, we will determine whether the study interventions alter the rate of decline in CD4+ counts by using a linear spline model with 2 time periods. The follow-up period will be divided into two periods: period 1 - from baseline until 6 months; period 2 - from 6 months until CD4+ count falls below 250/µL. Separate slope parameters will be fit for period 1 and period 2 using the linear spline model with a knot, t*, at 6 months. This approach will allow us 1) to analyze the magnitude of effect achieved during the punctuated treatment during period 1 and 2) to compare the rates of decline for the different treatment groups in period 2. The general model is given by:

$$\begin{matrix} E(y_{ij})=\gamma_{0G}+\gamma_{1G}t, t_{j}<t^{*} \\ E(y_{ij})=\gamma_{0G}+\gamma_{1G}t^{*}+\gamma_{2G}(t_{j}-t^{*}), t_{j}\geq t^{*} \end{matrix}$$

where y_ij_ is the CD4+ count for subject i (i = 1,2, … n) and measurement j (j = 1, 2, … m), G represents the two mutually exclusive treatment groups (punctuated ARV vs. reference group), and t* = 6 months on study. Using appropriate contrasts, we can test for differences in *γ*_11G_ between the groups assigned to punctuated therapy or delayed therapy for period 1. In this analysis, the main interest is not whether there will be a difference in CD4+ slope between the treated and delayed group, but instead what is the magnitude of the difference. This information will be useful in analyzing the response during period 2. Again using appropriate contrasts, we will compare the slopes of the two treatment groups during period 2. The null hypothesis for this analysis is: *γ*_2Punct ARV_ = *γ*_2Delay ARV_;; the alternative hypothesis is that the two groups will have different slopes in CD4+ measurement. To perform this analysis, we will use the linear mixed-effects model.^85^

$$CD4_{i}=\beta_{0}+\beta_{1}G+\beta_{2}t^{'}+\beta_{3}Gt^{'}+\beta_{4}(t_{j}-t^{*})_{+}+\beta_{5}G(t_{j}-t^{*})_{+}+Z_{i}b_{i}+\varepsilon_{ij}$$

where G represents the groups, as above, Z_i_ is a design matrix containing time information, b_i_ is a coefficient that captures random effects, ε_ij_ are independent Gaussian random variables with mean = 0 and variance σ^2^, t' = t_j_ – (t_j_ – t*), and (t_j_ – t*)_+_ = 0 when t_j_ ≤ t*. This is an attractive model for this analysis because it can incorporate missing or mistimed observations, accommodate additional covariate terms, allow for trends in variance over time, and allow for separate analysis of time trends and separate slopes.^86^ In this analysis, we will evaluate a series of models to estimate and compare mean slopes in period 2 for each treatment combination. In this formulation of the model, the global test of the null hypothesis *β*_5_ = 0 will assess whether slopes differ among groups in period 2. As with all longitudinal studies, missing data represents a challenge in the analysis and may bias the estimates. During the conduct of the study, every effort will be made to minimize the missing information. At the time of analysis, the data will be evaluated to determine whether CD4+ counts are missing at random by comparing the distributions of missing information between groups over time. If results of this analysis show similar distributions, then we will assume that the CD4+ values are missing at random and analyze with likelihood-based methods to avoid bias.^87^ If this assumption is not tenable, then we will need to make additional assumptions regarding the pattern of missing information before proceeding with the analysis.

#### **10.2.2.2 Specific Aim 2**

To determine whether a punctuated six-month course of ARV therapy during treatment of active TB will improve the response to antituberculous therapy, as measured by one month culture conversion, treatment failure, and relapse TB, among patients with active TB and CD4+ cell count above 350 cells/µL.

The analysis of this Specific Aim will include only patients with active TB that have been randomized in the clinical trial. The effect of ARV treatment on the response to antituberculous therapy will be evaluated by comparing the proportion of subjects with negative smears or cultures at one and two months (chi square test). A similar analysis will be performed for antituberculosis treatment failure in the ARV treatment arms compared to the reference arm.

Previous published studies of TB in HIV infection estimate relapse rates between 3 and 10%; we thus expect to identify between 10 to 35 relapse cases of TB during the study. The cumulative incidence of TB relapse will be estimated by group using Kaplan-Meier methods; incidence density methods will also be used to measure the number of relapses per 100 person-years of observation after completing antituberculous treatment according to ARV treatment group. The incidence density ratio (95% confidence interval) will be estimated to compare incidence in treated and reference groups. Cox regression analysis will be used to determine whether punctuated ARV therapy is associated with relapse after adjusting for covariates. The analysis will account for the different treatment histories by using time-dependent Cox proportional hazards analysis. The analysis will be further stratified according to relapses due to reactivation and reinfection based on the RFLP patterns.

#### **10.2.2.3 Specific Aim 3**

To determine whether a punctuated six-month course of highly active ARV therapy is safe when given with anti-tuberculosis therapy as measured by development of ARV drug resistance, toxicity and side effects, and paradoxical reactions.

Safety

The safety of the study interventions, including adverse events and paradoxical reactions will be examined by summarizing the proportion, or rate, of these events according to each study arm. We will compare the cumulative proportion of grade III/IV adverse reactions and paradoxical reactions between study arms with the chi-square or Fisher’s exact test. Time until first event will also be examined using Kaplan-Meier analysis with log-rank test to compare arms. Because an individual may have more than one adverse event, we will also estimate the incidence density of adverse events in each study arm and compare these rates by determining the relative risk of adverse events (with 95% confidence intervals) in the group receiving intervention compared with the reference group.

Viral drug resistance

Descriptive analyses of drug resistance mutations in reverse transcriptase will be performed. Based on HIV RNA patterns and values, samples will be assayed from patients who exhibit viral rebound during the punctuated therapy phase and, for a subset of 20 patients 2 months after completing punctuated therapy. If resistance is identified in samples off therapy, we will assay the plasma for persistence of the mutations. We will also assess drug resistance in all specimens from patients who exhibit virologic failure (confirmed HIV RNA >200 copies/ml after 6 months of therapy, Section 8.1.3) during the continuation phase (Step 3). Proportions and rates of drug resistance will be compared between the 2 strategies.

Analysis of Additional Outcomes

Analysis when CD4+ < 250/µL and Continuous ARV Therapy Begun

The main objective of this analysis is to determine whether punctuated ARV given during TB jeopardizes the response to continuous ARV therapy when given to participants who develop CD4+ count < 250/µL. In this analysis, there are 3 major outcomes of interest: CD4+ response, viral dynamics, and viral resistance to ARVs. Safety in terms of toxicity will also be evaluated. In terms of the CD4+ response, we will extend the linear spline model to include another knot, t**, at the time when an individual reaches CD4+ count < 250/µL. This modification of the model will allow us to compare the slope among patients who received punctuated therapy and those who did not. Viral dynamics, viral resistance and safety will be analyzed.

Ancillary Analyses

*Immunologic and Viral Dynamics (see appendix X.)*

#### In addition to the clinical endpoints, the investigators will evaluate surrogate makers for HIV disease progression and risk for tuberculosis. A series of analyses will be performed to describe viral kinetics in the first 3 months after starting therapy and to evaluate predictors of virologic and immunologic response to punctuated therapy. This analysis will also evaluate predictors of viral diversity and drug-resistance during and after the study intervention. A general approach to the analysis of censored biomarker measurements will be used to compare group responses.^88^ Investigators will also evaluate immune reconstitution during treatment for TB both with and without antiretroviral therapy. In this analyses, we will measure and compare serial responses to both HIV and TB specific antigens in whole blood cytokine assays, ElISpot assays, and activation markers (e.g., CD38, HLADR, CD4 and CD8). Archived samples collected as a routing part of the study will be used for these analyses

####

#### **10.2.2.4** **Additional Analyses**

The primary analysis as described will be performed according to the principles of intention to treat. In the event of withdrawal secondary to ineligibility, non-adherence, refusal, or toxicity, we will perform subgroup analyses of patients as treated, stratified by the degree of adherence. Propensity scores will be generated to capture the likelihood of non-adherence. Using the propensity scores, we will stratify patients according to the scores and examine the effect within and across strata. If the results of this ancillary analysis concur with the intention-to-treat analysis, then the interpretation of the results will be straightforward. If the results of this analysis differ from the intention-to-treat analysis, then the intention-to-treat analysis will be reported as the primary results because it is least likely to be biased.

## 10.3 Interim Analysis and Stopping Rules

### **10.3.1 Interim Analysis**

An interim analysis will be performed a few weeks after the first 20 subjects have been randomized into the study and have entered Step 2. A report of these cases will be submitted to the DSMB and the sponsor. For the remainder of the study, interim analyses will be performed annually. For each interim analysis, a standardized test statistic will be calculated for the main outcome (progression of HIV disease) based on accrued data. If this statistic exceeds the nominal critical value calculated using the error spending function (Table 10), then a statistically significant result will have been achieved at the time of that analysis. The sponsor will be notified and a report submitted for review by the NIAID Data Safety Monitoring Board (DSMB). In each interim analysis, the study team will present information on recruitment and the results of interim analyses to the DSMB which will review the data and recommend a course of action.

**Table 10. Schedule of 5 annual interim analyses to monitor disease progression**

This analysis assumes α=0.05 (two-sided test), O’Brien-Fleming boundaries (DeMets error-spending function), a rate of progression of 50% per year in the reference group and 25% per year in the treated group, and 350 trial participants.

| Year | Time | Information | Lower Bound | Upper Bound | Alpha | Cumulative alpha |
| --- | --- | --- | --- | --- | --- | --- |
| 1 | 0.2 | 0.29 | -4.8769 | 4.8769 | 0.00000 | 0.00000 |
| 2 | 0.4 | 0.50 | -3.3569 | 3.3569 | 0.00079 | 0.00079 |
| 3 | 0.6 | 0.62 | -2.6727 | 2.6724 | 0.00683 | 0.00762 |
| 4 | 0.8 | 0.76 | -2.2759 | 2.2550 | 0.01681 | 0.24420 |
| 5 | 1.0 | 0.83 | -1.9851 | 1.9612 | 0.02558 | 0.05000 |

10.3.2 Stopping Rules

The DSMB will determine whether to stop the study after a thorough review of interim data. The DSMB’s decision to stop the study will be guided by the critical values shown in Table 10. If, during an interim analysis, the critical value crosses the O’Brien-Fleming boundary, then the study will be stopped on statistical grounds as a significant difference between arms will have been observed. To evaluate trends in adverse events and deaths between the annual reviews of the DSMB, the study team will provide interim safety reports to the DAIDS Medical Monitor, and possibly a DSMB representative, pending discussion of the overall safety monitoring plan with the DSMB committee. These reports will provide cumulative enrollment figures, cumulative deaths and adverse events (classified according to grade), sorted by study arm. Brief clinical descriptions of key events will also be provided. If concerns arise from these interim reports, they will be forwarded to the full DSMB for review.

In the event of study termination, formal reports will be submitted to the DAIDS, NIAID, and local IRBs.

# 11.0 REGULATORY CONSIDERATIONS

## 11.1 Case Report Forms

All data will be recorded legibly in black ink on study forms. Corrections will be made by drawing a single horizontal line through the error (so that the original notation remains clearly legible). The correct information will be written near the original entry and dated and initialed by the investigator or designee.

All study documents, including patient records, laboratory data, and correspondence must be retained by the investigator for at least 5 years or until notification by or transfer to the study sponsor. All study documents are subject to inspection by the study Sponsor and appropriate regulatory authorities.

## 11.2 Monitoring

The study will be monitored by a representative of the Sponsor (Division of AIDS, NIAID, National Institutes of Health - US) to assure accurate and adequate data collection. The investigators will assist in making all study documents requested by the monitor available for review. Arrangements for monitoring visits will be arranged in advance between the investigator and the sponsor, except in the case of an emergency.

## 11.3 Institutional Review Board (IRB)

The protocol and informed consent document and any subsequent amendments will be reviewed and approved by the Institutional Review Boards or Ethics Committees of the Joint Clinical Research Center, Case Western Reserve University – University Hospitals of Cleveland, University of California, San Francisco – San Francisco General Hospital Campus at UCSF. Written documentation of approval of both the protocol and the informed consent must be provided to the TBRU Coordinating Center and Data Center at UCSF before starting the study.

The site Principal Investigator will ensure that an Ethics Committee/IRB will be responsible for the initial and continuing review and approval of the proposed clinical study. The site Principal Investigator or designee will promptly report to the Ethics Committee/IRB all changes in the research activity and all unanticipated problems involving risks to human subjects or others, and will not make changes in the research protocol without Ethics Committee/IRB approval, except where necessary to eliminate apparent immediate hazards to human subjects.

## 11.4 Data and Safety Monitoring Board

An independent Data and Safety Monitoring Board will be appointed by the Sponsor to monitor the study. The DSMB will meet at least annually and will be provided with updated information on the status of the study by the Sponsor (as provided by the PI) prior to each meeting. The principal investigator and other study personnel will be kept blinded to interim study data or analysis that is sorted by study arm. The study’s statistician, however, will have access to this information as needed for the analysis.

## 11.5 Informed Consent

Each study subject will give written informed consent (Appendix VIII and IX) both for the screening process to determine trial eligibility and for participation in the study. The informed consent documents will be translated into the major local language(s) and back‑translated to check for accuracy.

Informed consent will be obtained by a member of the study team, including the investigators or by professional staff supervised by the investigators, after verbal and written explanation of the study as described in the consent form. A copy of the informed consent document will be given to the subject. Appropriate pre‑ and post‑HIV‑test counseling of all study subjects will be performed by trained staff. Interpreters will be used as necessary. Subjects will have an opportunity to ask questions and raise any concerns about the study with the investigator and other study personnel. The informed consent document will be signed and dated (including the time the form is signed) by the subject and the investigator.

The investigator will insure that the purpose of the study is explained to the patient and that written consent is obtained prior to participation in the study. The patient, investigator, and others as required by local regulatory guidelines will sign the consent prior to entry into the study. The patient will receive a copy of the consent form. The investigator will retain a copy of the signed consent forms, which may be inspected at the monitor or auditor’s request.

**11.6 Amendments**

The study sponsor will be notified of any amendments to the study protocol. All protocol amendments will be submitted to the appropriate IRB(s) and Ethics Committees (Section 11.3) for review and will be approved prior to implementation at the study site except if necessary to implement a safety issue. The Principal Investigator at each clinical trial site will provide copies of the Ethics Committee approvals of amendments and any resultant revisions to the informed consent to the TBRU Coordinating Center at Case Western Reserve University.

## 11.7 Protocol Deviations

The study will be conducted as described in this protocol except in emergency situations in which the safety and well-being of a study subject requires immediate intervention in the judgment of the study site physician. Protocol deviations will be reported to the Coordinating Center and NIAID using the specified Protocol Violation/Deviation Form. Deviations should be reported to the Coordinating Center within 2 working days of the site staff learning of the event, and the Coordinating Center will in turn report these to NIAID within 2 working days following receipt.

## 11.8 Confidentiality

The Principal Investigator at each study site will ensure that all study records are managed in a secure and confidential fashion. Study identification numbers and subject initials will be used as identifiers on CRFs, clinical specimens, and computerized databases containing study information. Personal and medical information regarding study subjects will not the released to anyone other than authorized study personnel, the National Institutes of Allergy and Infectious Diseases and its authorized monitoring contractors, the Ethics Committees and Institutional Review Boards of the participating institutions, the OHPR, without the written permission of the subject. Study records will be secured in locked file cabinets at the clinical site.

## 11.9 Drug Accountability and Record Keeping

Study staff at each site are responsible for assuring that study drugs are dispensed to patients in compliance with the protocol. Study drug administration, in the form of directly-observed or self-supervised therapy, will be documented on the patient’s treatment card. The site will maintain a record of the source and lot numbers of drugs dispensed to study subjects.

# 12.0 References

1. Daley CL, Small PM, Schecter GF, Schoolnik GK, McAdam RA, Jacobs WR et al. An outbreak of tuberculosis with accelerated progression among persons infected with the human immunodeficiency virus. N Engl J Med 1992; 326(4):231-235.

2. Selwyn PA, Hartel D, Lewis VA, Schoenbaum EE, Vermund SH, Klein RS et al. A prospective study of the risk of tuberculosis among intravenous drug users with human immunodeficiency virus infection. N Engl J Med 1989; 320:545-550.

3. Jones BE, Young MM, Antoniskis D, Davidson PT, Kramer F, Barnes PF. Relationship of the manifestation of tuberculosis to CD4 cell counts in patients with human immunodeficiency virus infection. Am Rev Respir Dis 1993; 48:1292-1297.

4. Small PM, Schecter GF, Goodman PC, Sande MA, Chaisson RE, Hopewell PC. Treatment of tuberculosis in patients with advanced human immunodeficiency virus infection. N Engl J Med 1991; 324(5):289-294.

5. Whalen C, Horsburgh CR, Hom D, Lahart C, Simberkoff M, Ellner J. Accelerated course of human immunodeficiency virus infection after tuberculosis. Am J Respir Crit Care Med 1995; 151:129-135.

6. Leroy V, Salmi LR, Dupon M, Sentilhes A, Texier-Maugein J, Dequae L et al. Progression of human immunodeficiency virus in patients with tuberculosis disease. Am J Epidemiol 145[4], 293-300. 1997.

7. Tacconelli E, Tumbarello M, Ardito F, Cauda R. The impact of tuberculosis on survival of AIDS patients. 5th Conf Retrovir Oppor Infect 1998;(abstract no. 731).

8. Perneger TV, Sudre P, Lundgren JD, Hirschel B. Does the onset of tuberculosis in AIDS predict shorter survival? Results of a cohort study in 17 European countries over 13 years . BMJ 1995; 311:1468-1471.

9. Whalen CC, Nsubuga P, Okwera A, Johnson JL, Hom DL, Michael N et al. Impact of pulmonary tuberculosis on survival of HIV-infected adults: a prospective epidemiologic study in Uganda. AIDS 2000; 14:1219-1228.

10. Badri M, Ehrlich R, Wood R, Pulerwitz T, Maartens G. Association between tuberculosis and HIV disease progression in a high tuberculosis prevalence area . Int J Tuberc Lung Dis 2001; 5(3):225-232.

11. Munsiff SS, Alpert PL, Gourevitch MN, Chang CJ, Klein RS. A prospective study of tuberculosis and HIV disease progression. J AIDS 1998; 19:361-366.

12. Lawn SD, Shattock RJ, Acheampong JW, Lal RB, Folks TM, Griffin GE et al. Sustained plasma TNF-alpha and HIV-1 load despite resolution of other parameters of immune activation during treatment of tuberculosis in Africans. AIDS 1999; 13(16):2231-2237.

13. Pape JW, Jean SS, Ho JL, Hafner A, Johnson WD. Effect of isoniazid prophylaxis on incidence of active tuberculosis and progression of HIV infection. Lancet 1993; 342:268-272.

14. Wilkinson D, Squire SB, Garner P. Effect of preventive treatment for tuberculosis in adults infected with HIV: systematic review of randomised placebo controlled trials. BMJ 1998; 317:625-629.

15. de Pinho AM, Santoro-Lopes G, Harrison LH, Schechter M. Chemoprophylaxis for tuber culosis and survival of HIV-infected patients in Brazil. AIDS 2001; 15(16):2129-2135.

16. Cheynier R., Gratton S., Halloran M., Stahmer I., Letvin N.L., Wain-Hobson S. Antigenic stimulation by BCG vaccine as an *in vivo* driving force for SIV replication and dissemination. Nature Medicine 1998; 4(4):421-427.

17. Croix DA, Capuano S, III, Simpson L, Fallert BA, Fuller CL, Klein EC et al. Effect of mycobacterial infection on virus loads and disease progression in simian immuno-deficiency virus-infected rhesus monkeys. AIDS Res Hum Retroviruses 2000; 20;16(17):1895-1908.

18. Zhou D, Shen Y, Chalifoux L, Lee-Parritz D, Simon M, Sehgal PK et al. Mycobacterium bovis bacille Calmette-Guerin enhances pathogenicity of simian immunodeficiency virus infection and accelerates progression to AIDS in macaques: a role of persistent T cell activation in AIDS pathogenesis. J Immunol 1999; 162(4):2204-2216.

19. Shen Y, Shen L, Sehgal P, Zhou D, Simon M, Miller M et al. Antiretroviral Agents Restore Mycobacterium-Specific T-Cell Immune Responses and Facilitate Controlling a Fatal Tuberculosis-Like Disease in Macaques Coinfected with Simian Immunodeficiency Virus and Mycobacterium bovis BCG . J Virol 2001; 75(18):8690-8696.

20. Chan J, Kaufmann SHE. Immune Mechanisms of Protection. In: Bloom BR, editor. Tuberculosis: Pathogenesis, Protection, and Control. Washington, DC: ASM Press, 1994: 389-415.

21. Orenstein JM, Fox C, Wahl SM. Macrophage as a source of HIV during opportunistic infections. Science 276, 1857-1861. 1997.

22. Wallis RS, Vjecha M, Amir-Tahmasseb M, Okwera A, Byekwaso F, Nyole S et al. Influence of tuberculosis on human immunodeficiency virus enhanced cytokine expression and elevated β_2_ -microglobulin in HIV-1-associated tuberculosis. J Infect Dis 167, 43-48. 1993.

23. Valone SE, Rich EA, Wallis RS, Ellner J. Expression of tumor necrosis factor a in vitro by human mononuclear phagocytes stimulated with whole mycobacterium bovis BCG and mycobacterial antigens. Infect Immun 1988; 56:3313-3315.

24. Poli G, Bressler P, Kinter A, Duh E, Timmer WC, Rabson A et al. Interleukin 6 induces human immunodeficiency virus expression in infected monocytic cells alone and in synergy with tumor necrosis factor alpha by transcriptional and post-transcriptional mechanisms. J Exp Med 1990; 172(1):151-158.

25. Schrier R.D., McCutchan J.A., Wiley C.A. Mechanisms of immune activation of human immunodeficiency virus in monocytes/macrophages. J Virol 1993; 67(10):5713-5720.

26. Osborn L, Kunkel S, Nabel GJ. Tumor necrosis factor α and interleukin 1 stimulate the human immunodeficiency virus enhancer by activation of the nuclear factor kB. Proc Natl Acad Sci 86, 2336-2340. 1989.

27. Lederman MM, Georges DL, Kusner DJ, Mudido P, Giam CZ, Toossi Z. *Mycobacterium tuberculosis* and its purified protein derivative activate expression of the human immunodeficiency virus. J AIDS 7, 727-733. 1994.

28. Zhang Y, Nakata K, Weiden M, Rom WN. Mycobacterium tuberculosis enhances human immunodeficiency virus-1 replication by transcriptional activation at the long terminal repeat. J Clin Invest 95, 2324-2331. 1995.

29. Mayanja-Kizza H, Wajja A, Wu M, Peters P , Nalugwa G, Mubiru F et al. Activation of beta-chemokines and CCR5 in persons infected with human immunodeficiency virus type 1 and tuberculosis. J Infect Dis 2001; 183(12):1801-1804.

30. Toossi Z. Cytokine circuits in tuberculosis. Infectious Agents and Disease 1996; 5:98-107.

31. Goletti D, Weissman D, Jackson RW, Graham NMH, Vlahov D, Klein RS et al. Effect of Mycobacterium tuberculosis on HIV replication: Role of immune activation. J Immunol 157, 1271-1278. 1996.

1. Rich EA, Chen ISY, Zack JA, Leonard ML, O'Brien WA. Increases susceptibility of differentiated mononuclear phagocytes to productive infection with human immunodeficiency virus-1. J Clin Invest 1992; 89:176-183.

33. Nakata K, Rom WN, Honda Y, Condos R, Kanegasaki S, Cao Y et al. *Mycobacterium tuberculosis* enhances human immunodeficiency virus-1 replication in the lung. Am J Respir Crit Care Med 155, 996-1003. 1997.

34. Collins KR, Mayanja-Kizza H, Sullivan BA, Quinones-Mateu ME, Toossi Z, Arts EJ. Greater diversity of HIV-1 quasispecies in HIV-infected individuals with active tuberculosis. J Acquir Immune Defic Syndr 2000; 24(5):408-417.

35. Kirschner D. Dynamics of co-infection with *M. tuberculosis* and HIV-1. Theoretical Population Biology 1999; 55:94-109.

36. Corbett EL, De Cock KM. The clinical significance of interactions between HIV and TB: morequestions than answers. Int J Tuberc Lung Dis 2001; 5(3):205-207.

37. Del Amo J, Malin AS, Pozniak AL, De Cock KM. Does tuberculosis accelerate the progression of HIV disease? Evidence from basic science and epidemiology. AIDS 1999; 13:1151-1158.

38. Wallis RS, Nsubuga P, Whalen C, Mugerwa RD, Okwera A, Oette D et al. Pentoxifylline therapy in human immunodeficiency virus-seropositive persons with tuberculosis: A randomized, controlled trial. J Infect Dis 174, 727-733. 1996.

39. Klausner JD, Makonkawkeyoon S, Akarasewi P, Nakata K, Kasinrerk W, Corral L et al. The effect of thalidomide on the pathogenesis of human immunodeficiency virus type 1 and M. tuberculosis infection. J Acquir Immune Defic Syndr Hum Retrovirol 1996; 11(3):247-257.

40. Tramontana JM, Utaipat U, Molloy A, Akarasewi P, Burroughs M, Makonkawkeyoon S et al. Thalidomide treatment reduces tumor necrosis factor alpha production and enhances weight gain in patients with pulmonary tuberculosis. Mol Med 1[4], 384-397. 1995.

41. Palella FJ, Jr., Delaney KM, Moorman AC, Loveless MO, Fuhrer J, Satten GA et al. Declining morbidity and mortality among patients with advanced human immunodeficiency virus infection. HIV Outpatient Study Investigators. N Engl J Med 1998; 338(13):853-860.

42. Wong JK, Hezareh M, Günthard HF, Havlir DV, Ignacio CC, Spina CA et al. Recovery of replication-competent HIV despite prolonged suppression of plasma viremia. Science 1997; 278(5341):1291-1295.

43. Barreiro P, Soriano V, Blanco F, Casimiro C, de la Cruz JJ, Gonzalez-Lahoz J. Risks and benefits of replacing protease inhibitors by nevirapine in HIV-infected subjects under long-term successful triple combination therapy. AIDS 2000; 14(7):807-812.

44. Raffi F, Bonnet B, Ferre V, Esnault JL, Perre P, Reliquet V et al. Substitution of a nonnucleoside reverse transcriptase inhibitor for a protease inhibitor in the treatment of patients with undetectable plasma human immunodeficiency virus type 1 RNA. Clin Infect Dis 2000; 31(5):1274-1278.

45. Sterling TR, Chaisson RE, Bartlett JG, Moore RD. CD4+ lymphocyte level is better than HIV-1 plasma viral load in determining when to initiate HAART [Abstract 519] Program and Abstracts of the 8th Conference on Retroviruses and Opportunistic Infections. Chicago, IL:Alexandria,VA: Foundation for Retrovirology and Human Health. Foundation for Retrovirology and Human Health 199. 2001.

46. Hogg RS, Yip B, Chan KJ, Wood E, Craib KJ, O'Shaughnessy MV et al. Rates of disease progression by baseline CD4 cell count and viral load after initiating triple-drug therapy. JAMA 2001; 286(20):2568-2577.

47. US Department of Health and Human Services and the Henry J.Kaiser Family Foundation. Guidelines for the use of antiretroviral gents in HIV-infected adults and adolescents. http://www.hivatis.org/trtgdlns.html 2000.

1. Hammer S, Gibb D, Havlir DV, Mofensen L , Van Beek I, Vella S et al. Scaling up antiretroviral therapyin resource-limited settings: Guidelines for a public health approach. Executive Summary, 5-19. 2002. Geneva, WHO.

49. Tebas P, Henry K, Mondy K, Deeks S, valdez H, Cohen C et al. Effects of prolonged discontinuation of successful antiretroviral therapy. The 1st International Conference on HIV Pathogenesis and Treatment , Abstract #: 31. 7-8-2001.

50. Parish MA, Raines C, Higgins M, Gallant JE. Treatment discontinuation in patients with marginal indications for HAART. Interscience Conference on Antimicrobial Agents and Chemotherapy , Abstract #: I- 673. 9-22-2001.

51. Lundgren JD, Vella S, Paddam L, Blaxhalt A, Vetter N, Clumeck N et al. Interrupting/ stopping antiretrovial therapy and the risk of clinical disease: results from the EuroAIDS study. 9th Conference on Retroviruses and Opportunistic Infections , Abstract #: 48. 2-24-2002.

52. Narita M, Stambaugh JJ, Hollender ES, Jones D, Pitchenik AE, Ashkin D. Use of rifabutin with protease inhibitors for human immunodeficiency virus-infected patients with tuberculosis. Clin Infect Dis 2000; 30(5):779-783.

53. Gea-Banacloche JC, Lane HC. Immune reconstitution in HIV infection. AIDS 1999; 13( (suppl A)):S25-S38.

54. Carr A, Marriott D, Field A, Vasak E, Cooper DA. Treatment of HIV-1-associated microsporidiosis and cryptosporidiosis with combination antiretroviral therapy. Lancet 1998; 351(9098):256-261.

55. Hadad DJ, Lewi DS, Pignatari AC, Martins MC, Vitti JW, Arbeit RD. Resolution of Mycobacterium avium complex bacteremia following highly active antiretroviral therapy. Clin Infect Dis 1998; 26(3):758-759.

56. Miralles P, Berenguer J, Garcia d, V, Padilla B, Cosin J, Lopez-Bernaldo de Quiros JC et al. Treatment of AIDS-associated progressive multifocal leukoencephalopathy with highly active antiretroviral therapy . AIDS 1998; 12(18):2467-2472.

57. Nunn P, Brindle R, Carpenter L, Odhiambo J, Wasunna K, Newnham R et al. Cohort study of human immunodeficiency virus infection in patients with tuberculosis in Nairobi, Kenya. Am Rev Respir Dis 1992; 146:849-854.

58. Perriens JH, Colebunders RL, Karahunga C, Willame J, Jeugmans J, Kaboto M et al. Increased mortality and tuberculosis treatment failure rate among human immunodeficiency virus seropositive compared to HIV seronegative patients with pulmonary tuberculosis treated with 'standard' chemotherapy in Kinshasa, Zaire. Am Rev Respir Dis 1991; 144:750-755.

59. Serwadda D, Mugerwa RD, Sewankambo NK, Lwegaba A, Carswell JW, Kirya GB et al. Slim disease: a new disease in Uganda and its association with HTLV-III infection. Lancet 1985; %19;2(8460):849-852.

60. Eriki PP, Okwera A, Aisu T, Morrissey AB, Ellner JJ, Daniel TM. The influence of human immunodeficiency virus infection on tuberculosis in Kampala, Uganda. Am Rev Respir Dis 1991; 143:185-187.

61. Moher D, Schulz KF, Altman DG. The CONSORT statement: revised recommendations for improving the quality of reports of parallel-group randomized trials. Ann Intern Med 2001; 134(8):657-662.

62. Castro KG, Ward JW, Slutsker L, Buehler JW, Jaffe HW, Berkelman RL. 1993 Revised Classification System for HIV Infection and Expanded Surveillance
Case Definition for AIDS Among Adolescents and Adults . MMWR 41. 1992.

63. Sonnenberg P, Ross MH, Shearer SC, Murray J. The effect of dosage cards on compliance with directly observed tuberculosis therapy in hospital. Int J Tuberc Lung Dis 1998;2: 168‑171.

64. Katabira E., Kamya M.R., Mubiru F.X., Bakyaita N.N. HIV Infection: Diagnostic and Treatment Strategies for Healthcare Workers. 2 ed. Kampala, Uganda: Makerere University Printery, 2000.

1. Girling DJ. Adverse effects of antituberculosis drugs. Drugs 1982;23: 56-74.

66. Patel AM, McKeon J. Avoidance and management of adverse reactions to antituberculosis drugs. Drug Safety 1995;12: 1-25.

67. Kopanoff DE, Snider DE, Jr., Caras GJ. Isoniazid-related hepatitis: a U.S. Public Health Service cooperative surveillance study. Am Rev Respir Dis 1978;117:991-1001.

68. Nolan CM, Goldberg SV, Buskin SE. Hepatotoxicity associated with isoniazid preventive therapy: a 7-year survey from a public health tuberculosis clinic. JAMA 1999;281:1014-1018.

69. Ormerod LP, Horsfield N. Frequency and type of reactions to antituberculosis drugs: observations in routine treatment. Tubercle 1996;77:37-42.

70. Zierski M, Bek E. Side-effects of drug regimens used in short-course chemotherapy for pulmonary tuberculosis. A controlled clinical study. Tubercle 1980;61:41-49.

71. Hong Kong Chest Service/British Medical Research Council. Controlled trial of 4 three-times-weekly regimens and a daily regimen all given for 6 months for pulmonary tuberculosis. Second report: the results up to 24 months. Hong Kong Chest Service/British Medical Research Council. Tubercle 1982;63: 89-98.

72. Steele MA, Burk RF, Des Prez RM. Toxic hepatitis with isoniazid and rifampin: a meta-analysis. Chest 1991;99:465-71.

73. Halloran EM, Haber M, Longini IM, Struchiner CJ. Direct and indirect effects in vaccine efficacy and effectiveness. Am J Epidemiol 1991; 133(4):323-331.

74. Lee E.T. Nonparametric Methods for Comparing Survival Distributions. Statistical Methods for Survival Data Analysis. New York: Wiley, 1992: 66-100.

75. nQuery Advisor: User's Guide. Los Angeles, Ca: Statistical Solutions, Ltd., 1999.

76. Snedecor GW, Cochran WG. Statistical Methods. 7 ed. Ames: Iowa University Press, 1980.

77. Rothman KJ, Greenland S. Modern Epidemiology. second ed. Philadelphia: Lippincott - Raven, 1998.

78. Kaplan El, Meier P. Nonparametric estimation from incomplete observations. JASA 1958; 53:457-481.

79. Peto R, Pike MC, Armitage P, Breslow NE, Cox DR, Howard SV et al. Design and analysis of randomized clinical trials requiring prolonged observation of each patient. Part 2: analysis and examples. Br J Cancer 1977; 35:1-39.

80. Kleinbaum DG, Kupper LL, Morgenstern H. Measures of association. Epidemiologic Research: Principles and Quantitative Methods. New York: Reinhold, 1982: 140-158.

81. Cox DR. Regression models and life-tables (with discussion). J R Stat Soc B 1972; 34:187-220.

82. Kalbfleisch JD, Prentice RL. The Statistical Analysis of Failure-Time Data. New York: Wiley, 1980.

83. Pekovic V, Mayanja H, Vjecha M, Johnson J, Okwera A, Nsubuga P et al. Comparison of three composite compliance indices in a trial of self-administered preventive therapy for tuberculosis in HIV-infected Ugandan adults. Uganda-Case Western Reserve University Research Collaboration. J Clin Epidemiol 1998; 51(7):597-607.

84. Whalen CC, Johnson JL, Okwera A, Hom DL, Huebner R, Mugyenyi P et al. A Trial of Three Regimens to Prevent Tuberculosis in Ugandan Adults Infected with the Human

Immunodeficiency Virus. N Engl J Med 1997; 337(12):801-808.

85. Laird NM, Ware JH. Random-effect models for longitudinal data. Biometrics 1982; 38:963-974.

86. Cnaan A, Laird NM, Slasor P. Using the general linear mixed model to analyse unbalanced repeated measures and longitudinal data. Stat Med 1997; 16(20):2349-2380.

1. Albert PS. Longitudinal data analysis (repeated measures) in clinical trials. Stat Med 1999; 18(13):1707-1732.
2. Hughes MD. Analysis and design issues for studies using censored biomarker

measurements with an example of viral load measurements in HIV clinical trials. Stat Med 2000; 19(23):3171-3191.

**APPENDIX I. Study Schedule, SCREENING and STEP 1**

Delaying HIV disease Progression with Punctuated Antiretroviral Therapy in Patients with Tuberculosis Study Enrollment, Initiation of anti-TB treatment and Laboratory Testing Schedule

|  | **Screening and Enrollment**  **Study Weeks** | | | | | | |
| --- | --- | --- | --- | --- | --- | --- | --- |
|  | **SCREEN** | **STEP 1** | | | | |  |
|  | **Screen** | **Initiation of anti-TB treatment/**  **Baseline Visit** | **Week 1** | **Week 2** | **Week** **3** | **Week 4^e^*** | **SV**^f^ |
|  |  |  | Screen for eligibility for randomization **^c,d^** | | |  |  |
| **Informed consent** | X | X |  |  |  |  |  |
| **H&P, weight** | X | X | X^c^ | X^c, d^ | X^c, d^ |  | X |
| **New diagnoses/ hospitalizations survey** |  |  | X^c^ | X^c, d^ | X^c, d^ |  | X |
| **PA chest X-ray** | X |  |  |  |  |  | X |
| **AE Survey** |  |  | X | X^c, d^ | X^c, d^ |  | X |
| **HIV Testing algorithm** | X |  |  |  |  |  |  |
| **Sputum AFB smear & culture on solid media** | XXX |  |  |  |  |  | XX |
| **MTB drug susceptibility** | X |  |  |  |  |  |  |
| ***β*-HCG Pregnancy test (females)** | X |  | X^c, d^ | X^c, d^ | X^c, d^ |  | X |
| **CBC w/ differential & platelet count**^a^ | X |  | X^c, d^ | X^c, d^, | X^c, d^ |  | X |
| **AST, serum total bilirubin** | X |  | X^c, d^ | X^c, d^, | X^c, d^ |  | X |
| **Serum creatinine** | X |  | X^c, d^ | X^c, d^, | X^c, d^ |  | X |
| **Serum Glucose** | X |  |  |  |  |  |  |
| **CD4+ lymphocyte count** | X |  |  |  |  |  | X |
| **HIV RNA Roche Amplicor** |  | X |  |  |  |  | X |
| **Adherence questionnaire** (**TB treatment)** |  |  | X | X | X |  | X |
| **Quality of life assessment** |  |  | X |  |  |  |  |
| **Urine INH metabolite test** |  |  |  |  |  |  |  |
| **PBMC Storage/ Immune Function Test^h^** |  | X |  |  |  |  | X |
| **Plasma & Buffy Coat storage^i^** |  | X |  |  |  |  | X |
| **Disease specific diagnostic tests** |  |  | X | X | X |  | X |
| **Store MTB isolate for DNA fingerprinting**^b^ | XX |  |  |  |  |  | X |
| **Step 2 Baseline** |  |  |  | X^g^ | X^g^ | X^g^ |  |

Abbreviations: SV: Sick Visit; H&P: history and physical; AE: Adverse event; PA: posterior anterior; AFB: acid fast bacteria

^a^ CBC and platelet count should be repeated if there are clinical signs of anemia (pallor, hematemesis, hematocezia, etc.) or thrombocytopenia (petechiae, bruises, etc).

^b^ Two initial *M. tuberculosis* isolates from each subject will be stored at -70°C for later DNA fingerprinting if necessary. All isolates from positive cultures of suspected relapse cases will be stored at -70°C for DNA fingerprinting with the patient’s initial isolate.

^c^ Eligibility Criteria for randomization into study arm assessed at week 1 to be randomized on week 2. A patient that does not meet eligibility criteria based on contraindications for starting Trizivir, may be reassessed at Week 2 or 3 to be randomized one week after assessment. Laboratory values must be within acceptable range within 14 days prior to randomization

^d^ Week 1 assessments used as step 2 baseline status (week 2 or 3 for those patients not eligible at week 2)

^e^ For patients who are not randomized by week 4, they will receive standard TB treatment and follow-up per step 2 schedule

^f^ Laboratory, radiologic, and microbiologic tests will be performed at sick visits as clinically indicated

^g^ For patients randomized at week 2 or 3, and for all patients (randomized or not randomized) at week 4.

^h^Flow cytometrry, EliSpot, whole blood assays (3 green top tubes, JCRC)

^i^Viral dynamics (purple top tubes, CoreLab)

^j^Done on screening samples after positive culture

**APPENDIX I. Study Schedule, STEP 2**

Delaying HIV disease Progression with Punctuated Antiretroviral Therapy in Patients with Tuberculosis

Study Intervention, Follow-up and Laboratory Testing Schedule

|  | **STEP 2** | | | | | | | | | | | | | | | | | | | | | | | | |
| --- | --- | --- | --- | --- | --- | --- | --- | --- | --- | --- | --- | --- | --- | --- | --- | --- | --- | --- | --- | --- | --- | --- | --- | --- | --- |
|  |  | **Study Intervention Phase**  **(Months)** | | | | | | | | **Post-Study Intervention Follow-up (prior to study endpoint)**  **(Months)** | | | | | | | | | | | | | | | |
|  | Base  Line (Wk0) | Wk 1 | **Wk 2** | 1 | **2** | **3** | **4** | **5** | **6** | **7** | **8** | **9** | **10** | **11** | **12** | **15** | **18** | **21** | **24** | **27** | **30** | **33** | **36** | Extended Follow-up^a^ | SV |
| **H&P, weight** |  |  | X | X | X | X | X | X | X | X | X | X | X | X | X | X | X | X | X | X | X | X | X | X | X |
| **New diagnoses/ hospitalizations survey** |  |  | X | X | X | X | X | X | X | X | X | X | X | X | X | X | X | X | X | X | X | X | X | X | X |
| **PA chest X-ray ^b,c^** |  | week 8, and between weeks 22 & 24 from start of anti-TB Rx | | | | | | | |  |  |  |  |  | X |  | X |  |  |  |  |  |  |  | X^l^ |
| **AE Survey** |  |  | X | X | X | X | X | X | X | X | X | X | X | X | X | X | X | X | X | X | X | X | X | X | X |
| **Sputum AFB smear & culture on solid media ^c^** |  |  | between wks 4-6, week 8, and between weeks 18-20 from start of anti-TB Rx (x2) | | | | | | |  |  |  |  |  | XX |  | XX |  |  |  |  |  |  |  | X^l^ |
| **MTB drug susceptibility** |  |  | As clinically indicated | | | | | | | | | | | | | | | | | | | | | | |
| **Pregnancy test (females) ^d^** |  |  | X | X | X | X | X | X | X | As clinically indicated | | | | | | | | | | | | | | | |
| **CBC w/ differential & platelet count ^e^** |  |  | X | X | X | X |  |  | X |  |  | X |  |  | X | X | X | X | X | X | X | X | X | X | X^l^ |
| **AST, serum total bilirubin ^f, g^** |  |  | X | X | X | X |  |  | X | As clinically indicated | | | | | | | | | | | | | | | |
| **Serum creatinine ^h^** |  |  | As clinically indicated | | | | | | | | | | | | | | | | | | | | | | |
| **CD4+ lymphocyte count ^i^** |  |  | X | X |  | X |  |  | X |  |  | X |  |  | X | X | X | X | X | X | X | X | X | X | X^l^ |
| **HIV RNA Roche Amplicor** | X |  | X |  |  | X |  |  | X |  |  | X |  |  | X |  | X |  | X |  | X |  | X | X^m^ | X^l^ |
| **Adherence Questionaires (TB and ARV treatment) ^j^** |  |  | X | X | X | X | X | X | X |  |  |  |  |  |  |  |  |  |  |  |  |  |  |  | X |
| **Quality of life assessment** |  |  |  | |  |  |  |  | X |  |  |  |  |  | X |  | X |  | X |  | X |  | X |  |  |
| **Urine INH metabolite test** |  |  | X | X | X | X | X | X | X |  |  |  |  |  |  |  |  |  |  |  |  |  |  |  |  |
| **Plasma Storage^o^** | X | X | X | X | X | X |  |  | X |  |  | X |  |  | X |  | X |  | X |  | X |  | X | X | X |
| **PBMC Storage/ Immune Function Test^n^** | X |  | X |  |  | X |  |  | X |  |  | X |  |  | X |  |  |  |  |  |  |  |  |  | X |
| **Store MTB isolate for DNA fingerprinting ^k^** |  |  | As clinically indicated | | | | | | | | | | | | | | | | | | | | | | |
| **Disease specific diagnostic tests** |  |  | As clinically indicated | | | | | | | | | | | | | | | | | | | | | | |

Abbreviations: SV: Sick Visit; H&P: history and physical; AE: Adverse event; PA: posterior anterior; ^g^AST and bilirubin will be drawn to monitor hepatitis in all patients initiated on NVP regimen, at weeks 2, 4

AFB: acid fast bacteria and monthly for first 20 weeks after NVP is started. After the initial 20-week period, clinical and laboratory

^a^Patients will be followed through the 5-year time period of the study . Beyond the 36-month visit, monitoring should continue every 2 to 3 months throughout NVP treatment.

patients will be seen every 4 months and evaluated as per month 36. ^h^Serum creatinine will be repeated if clinically indicated.

^b^Chest X-ray will be performed at early discontinuation (if possible) unless done within the previous ^i^CD4+ lymphocyte count will be repeated within 1 month from a CD4+ result < 250/uL to establish study

2 weeks and in all patients with suspected relapsed tuberculosis. outcome based on two measures.

^c^Sputum AFB smear and cultures on solid media and posterior anterior chest X-ray will be ^j^Adherence questionnaires will be administered for the duration of TB and ARV therapy, even when longer

preformed as clinically indicated in evaluation of suspected TB relapses. than 6 months.

^d^Female patients receiving punctuated ARVs will have a pregnancy test during scheduled visits ^k^All isolates from positive cultures of suspected relapse cases will be stored at -70°C for DNA fingerprinting

while on this intervention. with the patient’s initial isolated.

^e^CBC and platelet count should be repeated if there are clinical signs of anemia (pallor, ^l^Laboratory, radiologic, and microbiology tests will be performed at Sick Visits as clinically indicated.

hematemesis, hematocezia, etc.) or thrombocytopenia (petechiae, bruises, etc). ^m^HIV RNA beyond 36 months will be obtained annually or as clinically indicated.

^f^AST and bilirubin will be drawn if patient develops symptoms of hepatitis (nausea, vomiting, ^n^ Flow cytometrry, EliSpot, whole blood assays (3 green top tubes, JCRC)

malaise, jaundice, or fatigue). LFTs will be obtained after one month of therapy if the AST is elevated. ^o^ Viral dynamics (purple top tube, CoreLab)

**APPENDIX I. Study Schedule, STEP 3**

Study Endpoint Confirmed: CD4+ < 200 or AIDS Defining Illness Post Primary Study Outcome Follow-up and Laboratory Testing Schedule

|  | **Study Endpoint Confirmation and Follow-up Study Months** | | | | | | | | | | | | | | | | | | | |
| --- | --- | --- | --- | --- | --- | --- | --- | --- | --- | --- | --- | --- | --- | --- | --- | --- | --- | --- | --- | --- |
|  |  | | **STEP 3** | | | | | | | | | | | | | | | | | |
|  |  | | **Post-Primary Study Outcome Assessment to Evaluate Secondary Study objectives – Safety and ARV resistance**  **Follow-up^b^ (months)** | | | | | | | | | | | | | | | | | |
|  | **Confirm Primary outcome^a^** | **ARV Safety^i^** | **Pre ARV Base-line** | **Wk 2** | **1** | **2** | **3** | **6** | **9** | **12** | **15** | **18** | **21** | **24** | **27** | **30** | **33** | **36** | **Extended Follow-up ^c^** | **SV** |
| **H&P, weight** | X | X |  | X | X | X | X | X | X | X | X | X | X | X | X | X | X | X | X | X |
| **AE Survey** | X | X |  | X | X | X | X | X | X | X | X | X | X | X | X | X | X | X | X | X |
| **New diagnosis/hospitalizations** | X | X |  | X | X | X | X | X | X | X | X | X | X | X | X | X | X | X | X | X |
| **Pregnancy test (females)** | X | X |  | If suspected | | | | | | | | | | | | | | | | |
| **CD4+ lymphocyte count** | X |  |  |  |  |  | X | X |  | X |  | X |  | X |  | X |  | X | X | X^i^ |
| **CBC w/ differential & platelet count ^d^** | X | X |  |  |  |  | X | X |  | X |  | X |  | X |  | X |  | X | X | X^i^ |
| **PA chest x-ray ^e^** |  |  | As clinically indicated | | | | | | | | | | | | | | | | | |
| **Sputum AFB smear & culture on solid media ^e^** |  |  | As clinically indicated | | | | | | | | | | | | | | | | | |
| **MTB drug susceptibility** |  |  |  |  |  |  |  |  |  |  |  |  |  |  |  |  |  |  |  | X^i^ |
| **Serum AST, serum bilirubin ^f, g^** | X | X |  |  | x | X |  | X |  | X |  | X |  | X |  | X |  | X | X | X^i^ |
| **Serum creatinine ^h^** | X | X | As clinically indicated | | | | | | | | | | | | | | | | | |
| **HIV RNA Roche Amplicor** |  |  | X |  | x | X | X | X |  | X |  | X |  | X |  | X |  | X | X | X^i^ |
| **ARV treatment started ^i^** |  |  | x |  | | | | | | | | | | | | | | | | |
| **ARV Adherence Questionnaire** |  |  |  |  | X | X | X | X | X | X | X | X | X | X | X | X | X | X | X | X |
| **Quality of life assessment** |  |  | X |  |  |  |  | X |  | X |  | X |  | X |  | X |  | X |  |  |
| **Plasma Storage^n^** |  |  | X |  | x | X | X | X |  | X |  | X |  | X |  | X |  | X | X | X |
| **PBMC Storage/ Immune Function Test^m^** |  |  | X |  |  |  | X | X |  |  |  |  |  |  |  |  |  |  |  | X |
| **HIV RNA genotyping ^j^** |  |  | As clinically indicated | | | | | | | | | | | | | | | | | |
| **Store MTB isolate for DNA fingerprinting ^k^** |  |  | As clinically indicated | | | | | | | | | | | | | | | | | |
| **Disease specific diagnostic tests** | X |  | As clinically indicated | | | | | | | | | | | | | | | | | |

Abreviations: SV: Sick Visit; H&P: history and physical; AE: Adverse event; PA: posterior anterior; ^f^AST and bilirubin will be drawn if patient develops symptoms of hepatitis (nausea, vomiting, malaise, jaundice, or

AFB: acid fast bacteria fatigue). LFTs will be obtained after one month of therapy if the AST is elevated.

^a^Primary Study Outcome is confirmed when the t2 CD4+ counts are found to be ^g^AST and bilirubin will be drawn to monitor hepatitis in all patients initiated on NVP regimen, at weeks 2, 4, and

below 250/mL within a 3 month time period or , an AIDS-defining condition (WHO stage IV classification **or**

a **definitive diagnosis of a severe bacterial infection that meets WHO Stage 3 classification** monthly for first 20 weeks after NVP is started. After the initial 20-week period, clinical and laboratory monitoring

should continue every 2 to 3 months throughout NVP treatment.

. ^h^Serum creatinine will be repeated if clinically indicated.

^b^The follow-up time post-event is contingent on the timeframe of the study. A patient will be ^i^ARV therapy will be started once safety is established. If patient doesn’t meet ARV safety evaluation then ARV

followed for a minimum of two years after enrollment of the last patient enrolled, for a maximum safety evaluation will be repeated monthly or as clinically indicated. If >2 Weeks have elapsed since time of

of 60 months. event, evaluation will be repeated before start of ARVs (section 5.3).

^c^Patients will be followed through the 5-year time period of the study . Beyond the 36-month ^j^HIV RNA genotyping will be performed on patients who fail to suppress HIV RNA < 200 copies/mL after 6 months

visit, patients will be seen every 4 months and evaluated as in month 36. of antiretroviral therapy.

^d^CBC and platelet count should be repeated if there are clinical signs of anemia (pallor, ^k^All isolates from positive cultures of suspected relapse cases will be stored at -70°C for DNA fingerprinting with

hematemesis, hematochezia, etc.) or thrombocytopenia (petechiae, bruises, etc). the patient’s initial isolate.

^e^Sputum AFB smear and cultures on solid media and posterior anterior chest X-ray will be ^l^Laboratory, radiolologic and microbiology tests will be performed at Sick Visits as clinically indicated.

performed as clinically indicated in evaluation of suspected TB relapses. ^m^Flow cytometrry, EliSpot, whole blood assays (3 green top tubes, JCRC) ^n^Viral dynamics (purple top tube, CoreLab)

**APPENDIX II.** **Modified WHO Staging System for HIV Infection and Disease in Adults and Adolescents, including definitive diagnostic methods and presumptive diagnostic criteria for diseases.**

| **Stage** | **Sign/ Symptom/ Disease** | **Definitive Diagnosis** | **Presumptive /clinical diagnosis** |
| --- | --- | --- | --- |
| Clinical stage I | Asymptomatic | Not applicable | No HIV related signs and symptoms |
|  | Persistent generalized lymphadenopathy | Histology | Painless enlarged lymph nodes >1 cm in two or more contiguous sites( excluding inguinal) in absence of known cause and persists for thee months or more |
| Clinical stage II | Weight loss, <10% of body weight | Documented weight loss<10% 0f body weight | Reported unexplained involuntary weight loss |
|  | Seborrheic dermatitis | Clinical diagnosis | Itchy scaly skin condition affecting hairy areas |
|  | Papular pruritic eruptions | Clinical diagnosis | Prurutic lesions often marked with post inflammatory pigmentation |
|  | Fungal nail infections | Clinical diagnosis | Paronychia,,onycholysis |
|  | Recurrent oral ulcerations( two or more episodes in last six months) | Clinical diagnosis | Apthous ulceration, typically painful with a halo of inflammation and a pseudo-membrane |
|  | Angular cheilitis | Clinical diagnosis | Splits or cracks at angle of the mouth, not due to vitamin or iron deficiency, respond to antifungal treatment |
|  | Herpes zoster | Clinical diagnosis | Painful vesicular rash in dermatomal distribution |
|  | Recurrent upper respiratory tract infections (current event plus one or more in last 6 months)) | Lab studies such as culture of suitable body fluid | Symptom complex, such as unilateral face pain( sinusitis), otitis media, tonsillopharyngitis without features of viral infection (such as coryza or cough) |

| Stage | Sign/ Symptom/ Disease | Definitive Diagnosis | **Presumptive /clinical diagnosis** |
| --- | --- | --- | --- |
| Clinical Stage III | Unexplained severe weight liss (> 10% of body weight) | Documented loss of more than 10% of body weight | Reported unexplained involuntary weight loss > 10% and visible thinning of face, waist and extremities with obvious wasting or BMI, 18.5 Kg/M^2^ |
|  | Unexplained chronic diarrhea, >1 month | Three or more stools observed and documented as unformed and two or more stools reveal no pathogens | Chronic diarrhea(loose or watery stools three or more times daily)reported for longer than one month |
|  | Unexplained persistenr fever (intermittent or constant), >1 month | Documented fever>37.5^0^C with negative blood culture,ZN, malaria slide, ,normal/unchanged Xray and no obvious focus of infection | Fever or night sweats>1 month, lack of response to antibiotics, antimalarial agents, with no other obvious foci of disease on examination. |
|  | Oral candidiasis (thrush) | Clinical diagnosis | Persistent or recurring curdlike plaques that can be scraped off or red patches on the toungue palate or lining of mouth |
|  | Oral hairy leukoplakia | Clinical diagnosis | Fine white small linear or corrugated lesions on lateral borders of the tongue that do not scrape off |
|  | Severe bacterial infections ( pneumonia,mengintis pyomyositis,bone or joint infection, bacteremia and severe pelvic inflammatory disease) | Isolation of bacteria from appropriate clinical specimens(usually sterile sites) | Fever accompanied by specific signs or symptoms that localize infection and response to appropriate antibiotic |
|  | Acute nerotizing ilcerative gingivitis or necrotizing ulcerative periodontitis | Clinical diagnosis | Severe pain, ulcerated gingival papillae, loosening of teeth, spontaneous bleeding, bad odour and rapid loss of bone and or soft tissue |
|  | Unexplained anemia(<8 g/dl),neutropenia(<0.5× 10^9^ per litre) or chronic(more than one month)thrombocytopenia(< 50× 10^9^ per litre) | Diagnosed on laboratory testing and not explained by other non-HIV conditions; ;not responding to standard therapy with heamatinics, antimalarial agents or anti helmintic agents | Not presumptive clinical diagnosis |

| Stage | Sign/ Symptom/ Disease | Definitive Diagnosis | Presumptive /clinical diagnosis |
| --- | --- | --- | --- |

| Clinical stage IV | HIV wasting syndrome | Documented weight loss>10% body weight  PLUS  two or more unformed stools negative for pathogens  OR  Documented temperature of > 37.5 ^0^C with no other cause of disease, negative blood culture, negative malaria slide and normal unchanged chest) | Unexplained involuntary weight loss(>10% baseline) with obvious wasting or BMI <18.5  PLUS  Unexplained chronic diarrhea reported for longer than 1 month  OR  Reports of fever or night sweats for moe than one month without oher cause and lack of response to antibiotics, antimalarial agents. Malaria must be excluded in malarious areas |
| --- | --- | --- | --- |
|  | Pneumocystis pneumonia | Ctology or immunofluorescent microscopy of induced sputum or bronchoalvealr lavage or histology of lung tissue | Dyspnea on exertion or non-productive cough of recent onset (within the previous 3 months)achypnoea and fever ; and  Chest radiograph evidence of diffuse bilateral interstitial infiltrates; and  Noevidence of a bacterial pneumonia cBilateral crepitations with or without reduced air entry |
|  | Recurrent Severe bacterial Pneumonia | Positive culture or antigen test of a compatible organism | Current episode plus one or more previous episodes in the past six months;acute onset(<2 weeks) of severe symptoms(such as fever,cough, dyspnoea and chest pain) PLUS new consolidation on clinical examination or chest Xray; ;response to antibiotics |
|  | Chronic herpes simplex virus infection( orolabial,genital or anorectal) of more than one month or visceral infection of any duration | Positive culture or DNA(by PCR) or compatible cytology or histology | Painful progressive anogenital or orolabial ulcerations, lesions caused by recurrence of herpes simplex virus infection and reported for more than one month. History of previous episodes. Visceral herpes simples virus requires definitive diagnosis |
|  | Esophageal candidiasis | Macroscopic appearance at endoscopy or bronchoscopy, or by microscopy or histology | Recent onset of retrosternal pain, or difficulty on swallowing(food and fluids) together with oral candida |
|  | Extra pulmonary tuberculosis | m. Tuberculosis isolation or compatible histology from appropriate site or radiological evidence of military TB | Ssystemic illness(suchas fever,nightsweats,weaknes and weightloss). Other evidence for extrapulmonary or disseminated TB varies by site.  Discreet peripheral lymphnode mycobacterium tuberculosis infection(especially cervical) is considered a less severe form of extrapulmonary tuberculosis |
|  | Kaposi Sarcoma | Microscopic appearance at endoscopy or bronchoscopy or by histology | Typical gross appeaence in skin, or oropharynx or persistent,initially flat,patches patches with a pink or violaceous colour,skin lesions that usually develop into plaques or nodule |

| Stage | Sign/ Symptom/ Disease | Definitive Diagnosis | Presumptive /clinical diagnosis |
| --- | --- | --- | --- |

|  | Cytomegalovirus disease(other than liver ,spleen or lymphnode) | Compatible histology or CMV demonstrated in CSF by culture or DNA(PCR) | rRetinitis only  Typical lesions on fundoscopic exam : discrete patches or fetinal whitening with distict borders spreading centrifugally often following blood vessels,associated with retinal vasculitis, hemorrhage and necrosis |
| --- | --- | --- | --- |
|  | CNS toxoplasmosis | Positive serum toxoplasma antibody AND (if available) single or multiple intracranial mass lesion on CT or MRI | Recent onset of focal nervous system abnomalities consistent with intracranial disease or reduced lvel of consciousness AND response within 10 day to specific therapy |
|  | HIV encephalopathy | Diagnosis of exclusion(if available)CT or MRI | Disabling cognitive and/or motor dysfunction interfering with activities of daily living, progressing over weeks or months in absence of a concurrent illness or condition other than HIV infection that might explain the findings |
|  | Extrapulmonary cryptococcosis(including meningitis) | Isolation of Cryptococcus neoformans from extrapulmonary site or positive cryptococcal antigen test on CSF | mMeningitis: usually subacute , fever with increasing severe headache, meningism, confusion,behaviour changes that respond to cryptococcal therapy |
|  | Disseminated non-tuberculous mycobacterial infection | Diagnosed by finding atypical mycobacterial species from stool,blood,body fluid or other body tissue, excluding the lungs | No presumptive clinical diagnosis |
|  | progressive multifocal leukoencephalopathy | Progressive nervous system disorder(cognitive dysfunction,gait/speech disorder,visual loss ,limb weakness and cranial nerve palsies) together with hypodense white matter lesions on neuro imaging or positive polymavirus JC PCR on CSF | No presumptive clinical diagnosis |
|  | Chronic cryptosporidiosis(with diarrhea lasting more than one month) | Cysts identified on modified ZN stain microscopic examination of unformed stool | No presumptive clinial diagnosis |
|  | Chronic Isosporiasis | identification of Isospora | No presumptive dianosis |
|  | Disseminated mycosis (such as coccidiomycosis,histoplasmosis or penicilliosis) | Histology,antigen detection orculture from clinical specimen or blood culture | No presumptive clinical diagnosis |
|  | Recurrent non-typhoid salmonella bacteraemia | Blood culture | No presumptive clinical diagnosis |
|  | Lympoma (cerebral or B-cell non-Hodgkin) | Histology of relevant specime or for central nervous system tumors, neuroimaging techniques | No presumptive clinical diagnosis |
|  | Invasive Cervical carcinoma | Histology or cytology | No presumptive clinical diagnosis |
|  | Visceral leishmaniasis | Diagnosed by histology(amastigotes visualized) or culture from any appropriate clinical specimen | No presumptive diagnosis |
|  | HIV –associated nephropathy | Renal biopsy | No presumptive clinical diagnosis |
|  | HIV associated cardiomyopathy | Cardiomegaly and evidence of poor left ventricular function confirmed by echocardiography | No presumptive clinical diagnosis |

**NOTE:** both definite and presumptive diagnosis are acceptable.

SOURCES: 1. ANNEX 1. WHO case definitions of HIV for surveillance and revised clinical staging and immunological classification of HIV –related disease in adults and children

**APPENDIX III. MU-JHU CORELAB Testing Algorithm for HIV Screening**

CORELAB TESTING ALGORITHM FOR HIV SCREENING –Jan 2003

| #1  Determine | #2  Statpak | #3  Unigold | DNA PCR  QL | Western Blot | Report as | Comment |
| --- | --- | --- | --- | --- | --- | --- |
| Negative |  |  |  |  | Negative |  |
| Positive | Positive |  |  |  | Positive |  |
| Positive | Negative | Negative |  |  | Negative |  |
| Positive | Negative | Positive |  |  | Positive |  |
| Invalid x 2 | Negative | Negative |  |  | Negative |  |
| Invalid x 2 | Positive | Positive |  |  | Positive |  |
| Invalid x 2 | Positive | Negative | x | x |  | Send for additional testing or report as indeterminate |
| Invalid x 2 | Negative | Positive | x | x |  | Send for additional testing or report as indeterminate |
| Positive | Invalid x 2 | Positive |  |  | Positive |  |
| Positive | Invalid x 2 | Negative | x | x |  | Send for additional testing or report as indeterminate |
| Positive | Negative | Invalid x 2 | x | x |  | Send for additional testing or report as indeterminate |

Reference Notes based on 2002 Corelab statistics:

1. Abbot Determine has a false positive rate of ≈ 1.7% (1-2 samples out of 100)
2. Approximately 16% of samples will require a second test( ie first test positive)
3. Approximately 2% of samples will require a third test ( ie first and second test discordant)
4. Less than 0.1% ( less than 1 out of 1000 samples) will require verification by DNAPCR or western Blot.

Manufacture Reference Information:

#1 Abbott Determine (Rapid) ([http://abbottdiagnostics.com](http://abbottdiagnostics.com/))

#2 Chembio HIV ½ Stat-Pak ([http://www.chembio.com](http://www.chembio.com/))

**#3 Trinity Uni-Gold (Rapid) (**[**http://www.trinitybiotech.com**](http://www.trinitybiotech.com/)**)**

**APPENDIX IV. Table For Grading Severity Of Adult Adverse Experiences**

**ABBREVIATIONS: Abbreviations utilized in the Table:**

ULN = Upper Limit of Normal LLN = Lower Limit of Normal

Rx = Therapy Req = Required

Mod = Moderate IV = Intravenous

ADL = Activities of Daily Living Dec = Decreased

**ESTIMATING SEVERITY GRADE**

For abnormalities NOT found elsewhere on the Tox Table, use the scale below to estimate grade of severity:

**GRADE 1 Mild** Transient or mild discomfort; no limitation in activity; no medical intervention/therapy required

**GRADE 2 Moderate** Mild to moderate limitation in activity - some assistance may be needed; no or minimal medical intervention/therapy required

**GRADE 3 Severe** Marked limitation in activity, some assistance usually required; medical intervention/therapy required, hospitalizations possible

**GRADE 4 Life-** Extreme limitation in activity, significant assistance

**threatening** required; significant medical intervention/therapy required, hospitalization or hospice care probable

**SERIOUS OR LIFE-THREATENING AEs**

ANY clinical event deemed by the clinician to be serious or life‑threatening should be considered a grade 4 adverse experience. Clinical events considered to be serious or life‑threatening include, but are not limited to:

seizures, coma, tetany, diabetic ketoacidosis, disseminated intravascular coagulation, diffuse petechiae, paralysis, acute psychosis

**MISCELLANEOUS**

> When two values are used to define the criteria for each parameter, the lowest values will be listed first.

> Parameters are generally grouped by body system.

> Some protocols may have additional protocol specific grading criteria.

**PARAMETER GRADE 1 GRADE 2 GRADE 3 GRADE 4**

MILD MODERATE SEVERE POTENTIALLY LIFE THREATENING

HEMATOLOGY

Hemoglobin 8.0 g/dL - 9.4 g/dL 7.0 g/dL - 7.9 g/dL 6.5 g/dL - 6.9 g/dL <6.5 g/dL

Absolute Neutrophil 1000 - 1500/mm^3^ 750 - 999/mm^3^ 500 - 749/mm^3^ <500/mm^3^

Count

Platelets 75,000 - 99,000/mm^3^ 50,000 - 74,999/mm^3^ 20,000 - 49,999/mm^3^ <20,000/mm^3^

Prothrombin Time (PT) >1.0 - 1.25 X ULN >1.25 - 1.5 X ULN >1.5 - 3.0 X ULN >3 X ULN

PTT >1.0 - 1.66 x ULN >1.66 - 2.33 x ULN >2.33 - 3.0 x ULN >3.0 x ULN

Methemoglobin 5.0 - 10.0% 10.1 - 15.0% 15.1 - 20.0% >20%

CHEMISTRIES

SODIUM

Hyponatremia 130 - 135 meq/L 123 - 129 meq/L 116 - 122 meq/L <116 meq/L

Hypernatremia 146 - 150 meq/L 151 - 157 meq/L 158 - 165 meq/L >165 meq/L

POTASSIUM

Hypokalemia 3.0 - 3.4 meq/L 2.5 - 2.9 meq/L 2.0 - 2.4 meq/L <2.0 meq/L

Hyperkalemia 5.6 - 6.0 meq/L 6.1 - 6.5 meq/L 6.6 - 7.0 meq/L >7.0 meq/L

PHOSPHATE

Hypophosphatemia 2.0 - 2.4 mg/dL 1.5 - 1.9 mg/dL 1.0 - 1.4 mg/dL <1.0 mg/dL

CALCIUM - (corrected for albumin)

Hypocalcemia 7.8 - 8.4 mg/dL 7.0 - 7.7 mg/dL 6.1 - 6.9 mg/dL <6.1 mg/dL

Hypercalcemia 10.6 - 11.5 mg/dL 11.6 - 12.5 mg/dL 12.6 - 13.5 mg/dL >13.5 mg/dL

MAGNESIUM

Hypomagnesemia 1.2 - 1.4 meq/L 0.9 - 1.1 meq/L 0.6 - 0.8 meq/L <0.6 meq/L

BILIRUBIN

Hyperbilirubinemia >1.0 - 1.5 x ULN >1.5 - 2.5 x ULN >2.5 - 5 x ULN >5 x ULN

**PARAMETER GRADE 1 GRADE 2 GRADE 3 GRADE 4**

MILD MODERATE SEVERE POTENTIALLY LIFE THREATENING

GLUCOSE

Hypoglycemia 55 - 64 mg/dL 40 - 54 mg/dL 30 - 39 mg/dL <30 mg/dL

Hyperglycemia 116 - 160 mg/dL 161 - 250 mg/dL 251 - 500 mg/dL >500 mg/dL

(nonfasting and

no prior diabetes)

Triglycerides ________ 400 - 750 mg/dL 751 - 1200 mg/dL >1200 mg/dL

Creatinine >1.0 - 1.5 x ULN >1.5 - 3.0 x ULN >3.0 - 6.0 x ULN >6.0 x ULN

URIC ACID

Hyperuricemia 7.5 - 10.0 mg/dL 10.1 - 12.0 mg/dL 12.1 - 15.0 mg/dL >15.0 mg/dL

LIVER TRANSAMINASE (LFTs)

AST (SGOT) 1.25 - 2.5 x ULN >2.5 - 5.0 x ULN >5.0 - 10.0 x ULN >10.0 x ULN

ALT (SGPT) 1.25 - 2.5 x ULN >2.5 - 5.0 x ULN >5.0 - 10.0 x ULN >10.0 x ULN

GGT 1.25 - 2.5 x ULN >2.5 - 5.0 x ULN >5.0 - 10.0 x ULN >10.0 x ULN

Alk Phos 1.25 - 2.5 x ULN >2.5 - 5.0 x ULN >5.0 - 10.0 x ULN >10.0 x ULN

PANCREATIC ENZYMES

Amylase >1.0 - 1.5 x ULN >1.5 - 2.0 x ULN >2.0 - 5.0 x ULN >5.0 x ULN

Pancreatic amylase >1.0 - 1.5 x ULN >1.5 - 2.0 x ULN >2.0 - 5.0 x ULN >5.0 x ULN

Lipase >1.0 - 1.5 x ULN >1.5 - 2.0 x ULN >2.0 - 5.0 x ULN >5.0 x ULN

**PARAMETER GRADE 1 GRADE 2 GRADE 3 GRADE 4**

MILD MODERATE SEVERE POTENTIALLY LIFE THREATENING

CARDIOVASCULAR

Cardiac Arrhythmia ________ Asymptomatic; Recurrent/persistent Unstable dysrhythmia,

transient dysrhythmia, dysrhythmia; symptomatic hospitalization and

no Rx req Rx req Rx req

Hypotension Transient orthostatic Symptoms correctable IV fluid req, no Hospitalization req

hypotension, no Rx with oral fluid Rx hospitalization req

Hypertension Transient, increase Recurrent; chronic Acute Rx req; outpatient Hospitalization req

>20 mm/Hg; no Rx increase >20 mm/Hg, hospitalization possible

Rx req

Pericarditis Minimal effusion Mild/mod asymptomatic Symptomatic effusion, Tamponade OR pericardi-

effusion, no Rx pain, EKG changes iocentesis OR surgery req

Hemorrhage, blood ________ Mildly symptomatic, Gross blood loss OR Massive blood loss OR

Loss no Rx required 1-2 units transfused >2 units transfused

GASTROINTESTINAL

Nausea Mild OR transient; Mod discomfort OR Severe discomfort OR Hospitalization req

reasonable intake intake decreased minimal intake for

maintained for <3 days >3 days

Vomiting Mild OR transient; Mod OR persistent; Severe vomiting of all Hypotensive shock OR

2-3 episodes per day 4-5 episodes per day; food/fluids in 24 hrs OR hospitalization req for

OR mild vomiting OR vomiting lasting orthostatic hypotension IV Rx req

lasting <1 week >1 week OR IV Rx req

Diarrhea Mild OR transient; Mod OR persistent; Bloody diarrhea; OR Hypotensive shock OR

3-4 loose stools per 5-7 loose stools per orthostatic hypotension hospitalization req

day OR mild diarrhea day OR diarrhea OR >7 loose stools/day

lasting <1 week lasting >1 week OR IV Rx required

Oral Discomfort/ Mild discomfort, Difficulty swallowing Unable to swallow solids Unable to drink fluids;

Dysphagia no difficulty but able to eat and IV fluids req

swallowing drink

Constipation Mild Moderate Severe Distention w/vomiting

**PARAMETER GRADE 1 GRADE 2 GRADE 3 GRADE 4**

MILD MODERATE SEVERE POTENTIALLY LIFE THREATENING

RESPIRATORY

Cough (for Transient; no Rx Treatment associated Uncontrolled cough; ________

aerosol studies) cough; inhaled systemic Rx req

bronchodilator

Bronchospasm Acute Transient; no Rx; Rx req; normalizes No normalization Cyanosis; FEV1 <25%

FEV1 70% - <80% with bronchodilator; with bronchodilator; (or peak flow) OR

(or peak flow) FEV1 50%-<70% (or FEV1 25% - <50% (or intubated

peak flow) peak flow), retractions

Dyspnea Dyspnea on Dyspnea with normal Dyspnea at rest Dyspnea requiring

exertion activity O_2_ therapy

NEUROLOGIC

Neuro-cerebellar Slight incoordination Intention tremor OR Ataxia requiring Unable to stand

OR dysdiadochokinesia dysmetria OR slurred assistance to walk or

speech OR nystagmus arm incoordination

interfering with ADLs

Neuro-psych/mood ________ _________ Severe mood changes Acute psychosis req

requiring medical hospitalization

intervention

Paresthesia Mild discomfort; no Mod discomfort; Severe discomfort; OR Incapacitating; OR

(burning, Rx req non-narcotic narcotic analgesia req not responsive to

tingling, etc) analgesia req with symptomatic narcotic analgesia

improvement

Neuro-motor Mild weakness in Mod weakness in feet Marked distal weakness Confined to bed or

muscle of feet but (unable to walk on (unable to dorsiflex wheel chair because of

able to walk and/or heels and/or toes), toes or foot drop), and muscle weakness

mild increase or mild weakness in mod proximal weakness

decrease in reflexes hands, still able to e.g., in hands interfering

do most hand tasks with ADLs and/or requiring

and/or loss of assistance to walk and/or

previously present unable to rise from chair

reflex or development unassisted

of hyperreflexia and/or

unable to do deep knee

bends due to weakness

**PARAMETER GRADE 1 GRADE 2 GRADE 3 GRADE 4**

MILD MODERATE SEVERE POTENTIALLY LIFE THREATENING

Neuro-sensory Mild impairment (dec Mod impairment (mod Severe impairment Sensory loss involves

sensation, e.g., dec sensation, e.g., (dec or loss of limbs and trunk.

vibratory, pinprick, vibratory, pinprick, sensation to knees or

hot/cold in great toes) hot/cold to ankles) wrists) or loss of

in focal area or and/or joint position sensation of at least

symmetrical distri- or mild impairment mod degree in multiple

bution that is not different body areas

symmetrical (i.e., upper and lower

extremities)

URINALYSIS

Proteinuria

Spot urine 1+ 2 - 3+ 4+ Nephrotic syndrome

24 hour urine 200 mg-1 g loss/day OR >1 - 2 g loss/day OR >2 - 3.5 g loss/day OR Nephrotic syndrome OR

<0.3% OR 0.3 - 1.0% OR >1.0% OR >3.5 g loss/day

<3 g/l 3 - 10 g/l >10 g/l

Gross Hematuria Microscopic only Gross, no clots Gross plus clots Obstructive OR

transfusion req

MISCELLANEOUS

Fever 37.7 - 38.5C OR 38.6 - 39.5C OR 39.6 - 40.5C OR >40.5C OR

oral >12 hours 100.0 - 101.5F 101.6 - 102.9F 103 - 105F >105F

Headache Mild; no Rx req Mod; or non-narcotic Severe; OR responds to Intractable; OR requiring

analgesia Rx initial narcotic Rx repeated narcotic Rx

Allergic Reaction Pruritus without Localized urticaria Generalized urticaria Anaphylaxis

rash angioedema

Cutaneous/Rash/ Erythema, pruritus Diffuse maculopapular Vesiculation OR moist ANY ONE: mucous membrane

Dermatitis rash OR dry desquamation OR involvement, suspected

desquamation ulceration Stevens-Johnson (TEN),

erythema multiforme, necrosis req surgery/ exfoliative dermatitis

**PARAMETER GRADE 1 GRADE 2 GRADE 3 GRADE 4**

MILD MODERATE SEVERE POTENTIALLY LIFE THREATENING

Local Reaction Erythema Induration <10mm OR Induration >10mm OR Necrosis of skin

(2^o^ parenteral Rx - inflammation OR ulceration

not vaccination or phlebitis

skin test)

Fatigue Normal activity Normal activity Normal activity reduced Unable to care for

reduced <25% reduced 25-50% >50%; cannot work self

**APPENDIX V. Guidelines For Estimating Grade Of Severity For Adverse Experiences Not Otherwise Specified**

| **CRITERIA** | **GRADE 1 TOXICITY** | **GRADE 2 TOXICITY** | **GRADE 3 TOXICITY** | **GRADE 4 TOXICITY** |
| --- | --- | --- | --- | --- |
| KARNOFSKY SCORE  OR  SELF CARE ACTIVITIES, IMPACT  ON ACTIVITIES OF  DAILY LIVING (ADL)  OR  MEDICAL CARE  NEEDED | 80-90  Transient or mild discomfort, no limitations on activity.  No medical treatment required; monitor condition | 70-80  Mild to moderate  discomfort; may work full-time; some assistance may be required  May require minimal medical treatment or monitoring | 50-70  Marked impact; ADLs limited; may work part-time with some assistance  Requires medical care and possible hospitalization | < 50  Completely disabled; needs full assistance to perform ADLs; unable to work  Requires active medical treatment and hospitalization |

**APPENDIX VI. Paradoxical Clinical Reactions in TB Subjects Treated with**

**Highly Active Antiretroviral Therapy**

Background

In patients treated for TB, transient clinical deterioration has been observed. Most commonly this has been reported in patients with tuberculomas or lymphadenopathy. These “paradoxical” reactions have been attributed to enhanced immunologic recognition and inflammatory response of tuberculous antigens. They are usually self-limiting, but often require steroids for symptomatic relief.

Recently, Narita et al. (1998) reported a high frequency (36%) of paradoxical reactions in HIV-infected subjects undergoing treatment for TB. These reactions typically occurred about 2 weeks after the initiation of highly active antiretroviral therapy (HAART) and were characterized by fever and worsening adenopathy and/or pulmonary infiltrates, or central nervous system abnormalities in those with evidence of tuberculosis DNC disease.

Definition of Suspected Paradoxical Reactions

Paradoxical reactions are defined as (1) new persistent fevers (temperature ≥ 101.5°F) which develop after the initiation of HAART and which last for more than 1 week without an identifiable source (e.g., multiple blood cultures, urine and sputa testing, and other procedures when clinically indicated); OR (2) marked worsening or emergence of intrathoracic lymphadenopathy, pulmonary infiltrates; OR (3) worsening or emergence of cervical adenopathy on serial physical examinations, OR worsening of other tuberculous lesions or manifestations, such as cutaneous peritoneal or central nervous system (CNS inflammatory pathology.

References

Begon, M, Harper, JL, Townsend CR. Ecology: individuals populations and communities. Blackwell Scientific, Oxford,1986.

Chloremis, CB, Padiatellis, C, Zoumboulakis, D, Yannakos, D. Transitory exacerbation of fever and roentgenographic findings during treatment of tuberculosis in children. Am Rev Tuberc 1955;75:527-36.

McLean AR, Emery VC, Webster A, Griffiths PD. Population dynamics of HIV within individual after treatment with zidovudine. AIDS 1991;5:485-9.

Narita M, Ashkin, D, Hollender, ES, Pitchenik, AE. Paradoxical worsening of tuberculosis following antiretroviral therapy in patients with AIDS. Am J Respir Crit Care Med 1998;158: 157-61.

Vanham G, Edmonds K, Qing, L et al. Generalized immune activation in pulmonary tuberculosis: co-activation with HIV infection. Clin Exp Immunol 1996;103:30-4

**APPENDIX VII. Internal Quality Management Plan**

Overview

Quality Management (QM) is the overall process comprising both Quality Assurance and Quality Control activities into a planned and systematic program at all clinical research sites.

- Quality Control (QC) is defined as the prospective review process of 100% of the collected data. These activities are generally carried out by the individuals involved in the daily data collection and management process.
- Quality Assurance (QA) is defined as the retrospective sampling of records to compare source documents to completed case report forms. Consistency and completeness of documentation can also be evaluated at this time. QA activities are generally carried out by an individual with a clinical background.

The goal of QM is to promote the collection of valid data by continually evaluating the systems for collecting and reviewing data. While the QM Plan was developed for the TBRU, it will be applied to other research activities undertaken by the Uganda-CWRU Research Collaboration. The QM plan has the following components:

- Regularly scheduled QA and QC activities
- Evaluation of QA and QC findings
- Identification of areas in need of improvement
- Implementing corrective actions as needed
- Periodic review of plan for personnel compliance
- Annual review of QM plan and revision as needed
- Points of quality management

Quality Management Activities

*This section describes the QA and QC procedures that are employed at each of the nine stages of clinical research.*

A. Quality Control

There are nine points in the course of clinical research where Quality Control Activities are implemented (see figure). The key points are listed below with the associated quality measures.

1. Prior to patient contact:

All patient charts will be checked by filing staff to assure that protocol-specified laboratory requisitions and data collection forms are in place before the patient visit. These forms and requisitions are relevant not only to the protocol, but also to the interval on study.

2. Point of patient contact:

After a subject has been evaluated and before leaving the clinic or before a research team leaves a subject’s home, data collection forms which were completed at that contact will be cross-checked by another member of the research team. Any queries will be resolved at that time before ending the contact with the subject. This is a review for completeness of data collection and is not intended to be a clinical review.

3. Twenty-four hours after patient contact:

Within twenty-four hours of the patient contact, the Clinical Coordinator will review all documentation for the current visit in the context of prior documentation and patient management. This review will generate further patient evaluation, if necessary, and assure protocol and GCP compliance.

4. Scanning:

Automated data entry system is utilized. The first step in this system is the creation of an electronic image of the data collection instrument via scanning at the research site. These electronic images are then transferred to the Data Coordinating Center for optical character recognition automated data entry. At the time of scanning, all key variables on all data collection instruments will be reviewed by Key QC Personnel or other data team members under the direction of Key QC Personnel. This objective of this review is to make sure that all fields are complete and any out of range laboratory value is appropriately addressed. If necessary, queries will be generated and resolved by site clinical staff prior to the creation of an electronic image. In the event that a research site is unable to produce electronic images of data collection instruments, the key variables will be reviewed prior to transmission of paper images of the forms to the Data Coordinating Center.

5. Receipt of data at the Data Coordinating Center:

The remote research sites should provide the Data Coordinating Center with an inventory of forms that are being transferred. The Data Coordinating Center will compare the inventory of forms sent with those received. Any discrepancies will be queried as described below.

Upon arrival at the Data Coordinating Center, all electronic or paper data collection instruments are reviewed by visual inspection. This visual inspection will be performed on 100% of variables on all forms and will target:

missing variables

incorrect ID numbers

adherence to protocol

appropriate documentation of out of range laboratory values and adverse events.

This process will be executed by Data Coordinating Center Key QC Personnel. If necessary, queries will be generated and resolved by the site clinical staff at this step. The query process is further defined in the next section.

6. Automated entry:

At the time of automated entry, all variables will be verified for accurate optical character recognition. Databases will also be reviewed at this point to validate appending of new entries to the Access databases. This process will be conducted under the direction of Data Coordinating Center Key QC Personnel.

7. Database program check:

After data entry, the Access databases will be imported in SAS where programs will be run by Data Coordinating Center Key QC Personnel to:

Identify missing variables

Perform logic checks

Identify data entry errors

Determine frequencies of variables and measures of central tendency

Identify adverse events

These programs will be written with input from the Medical Officers, PI, or other clinical personnel. This process will be executed by Coordinating Center Key QC Personnel. If necessary, queries will be generated and resolved by the site clinical staff at this step.

8. Merging Data:

After database cleaning programs have been run, multiple databases will be merged to form one analytical dataset. SAS programs will be run to validate the merging process. This step will be performed by Coordinating Center Key QC Personnel. If necessary, queries will be generated and resolved by the site clinical staff at this step.

9. Analytical Coding:

As data analysis program are written and tested, data will be evaluated for consistency and accuracy. If necessary, queries will be generated and resolved by the site clinical staff at this step. This step will be performed by Coordinating Center Key QC Personnel or the Data Analyst.

B. Quality Assurance

Quality Assurance activities are conducted semi-annually on a sample of patient/subject charts. This is a retrospective, longitudinal review of patient records. Coordination and implementation of these activities are the responsibility of Key QA Personnel. QA activities include, but is not limited to:

1. Monitoring of study protocols – Key QA Personnel will make sure that subject visits and test schedules are adhered to according to the approved protocol.
2. Performing data/process audits according to schedule – Semi-annually, a sample of the current enrollment from each study will be audited. Key QA Personnel will facilitate this process including review of subject records, communication of audit findings to Coordinating Center, and implementation of any resultant corrective measures.
3. Facilitation of peer audits: Key QA Personnel will work with the Medical Officers and Clinical Coordinators to facilitate peer audits of subject records as requested by the Regulatory Consultant. QA Personnel will then compare audit findings, address any resultant queries, and in-service clinical staff as needed.
4. Provide timely reports to the Investigators and Component as requested, on the progress of a protocol or any deviation from an approved protocol.
5. Audit a defined number of cases at the start of a new protocol and forward QA results to the Regulatory Consultant for compilation and report generation. Key QA Personnel will also be an active participant in resultant activities such as form revision or further training of clinical staff.
6. Review of study regulatory binders at least twice annually to make sure it is complete and current.

Tools

*This section further defines specific tools utilized in the QM plan.*

Quality Control

Quality Control tools include but are not limited to:

- Quarterly study-specific reports summarizing number and type of queries will be generated by Coordinating Center Key QC Personnel and distributed to the PI, Protocol Team, and Regulatory Consultant.
- The Coordinating Center Key QC Personnel will also produce quarterly study-specific reports on query resolution time.
- Frequency statistics or measures of central tendency on key variables will be reported monthly to the PI and Protocol Team

Quality Assurance

Quality Assurance tools include but are not limited to:

- A thorough review all documentation from the first 5 patients enrolled in a new study will take place
- Semi-annually, a percent of the current enrollment of each study will be audited
- Audit findings will be forwarded to the Regulatory Consultant for compilation, report preparation, and identification of areas in need of improvement.

A QA tool (Attachment I) has been developed to facilitate standard and complete QA audit. The QA tool will be completed at the remote research sites for each patient audited and returned to the Regulatory Consultant.

Regulatory

A Regulatory Review Tool (Attachment II) has been developed to facilitate standard and complete review of study regulatory documentation. The regulatory tool will be completed each time a regulatory binder is audited. The completed tool will be promptly forwarded to the Regulatory Consultant who will work with Key QA Personnel to facilitate obtaining any missing or incomplete documents.

Key Indicators

*This section describes key variables/indicators which will be closely evaluated in the execution of the Quality Management Plan.*

Quality Control

- Missing data points
- Laboratory values out of range without comment
- Data consistency
- Proper use of GCP in making corrections to CRFs
- Key variables on-site, all variables at the Coordinating Center
- Logic

Quality Assurance

- Informed consent
- Eligibility criteria
- Laboratory tests
- Concomitant medications
- Adverse Event reporting
- Study/clinical endpoints
- Study drug/dose management
- Missed visits
- TB treatment/drug susceptibility
- Treatment/study discontinuation
- TB treatment outcome

Regulatory

- Study protocol
- Amendments to protocol
- Informed consent form (IRB approved version)
- IRB approval documentation for all protocol versions
- Annual progress report and re-approvals
- FDA 1572 Statement of Investigator
- CVs for all investigators
- Laboratory certification
- Laboratory normal range values for all participating labs
- Site monitoring sign-in log
- Severe adverse event reports
- All correspondence (except financial)
- Investigator drug brochure
- Signature list for study staff
- Screening log

Audit Priorities

*This section defines how audit priorities will be determined in the execution of the Quality Management Plan.*

Quality Control

Certain documentation will be subject to one hundred percent review in the execution of this plan including but not limited to:

- Key variables will be reviewed by the research site data management staff prior to transfer of data to the Coordinating Center.
- After data is transferred to the Coordinating Center, a 100% review of all variables will be conducted through visual inspection
- Computer programs will be used to review 100% of data collected at the point of database cleaning, data merge, and analytical coding.
- All severe adverse events
- All recognized protocol violations
- All main study outcomes
- All deaths on study

Quality Assurance

Quality assurance audits will be conducted semi-annually. QA audits will focus on process and outcome. QA audits of process will be performed through site visits, key steps in data collection, and patient management. QA audits of outcome will be performed by selecting a ten percent sample of the current enrollment of each trial will be selected for peer review audit. The ten percent selection will be based on new or unexpected SAEs occurring in the last six months, any patient terminated during the last six months, and a random selection. All study outcome events, serious adverse events, and deaths will be in a timely manner by qualified personnel. A greater percentage of cases may be selected for audit if documentation for a particular protocol is found to be problematic. The Component Leader, Regulatory Consultant, Senior Data Manager and Key QA personnel may be involved in determining which cases are to be audited.

In addition to the semi-annual audit, the documentation for the first 5 patients enrolled in a new protocol will be audited at 4, 8, and 12 weeks post enrollment. This audit of new protocols will be completed by the Key QA personnel identified at each site.

Regulatory

A review of the regulatory binder for each protocol will be conducted upon site visits by the Coordinating Center Staff. In some instances, the regulatory binder may be shipped to the Coordinating Center for review. Each regulatory binder will be audited no less than twice annually.

New Staff

Documentation generated by new staff members will be audited for the first 5 cases per protocol by peer review. This process will be implemented by the Key QA person at each site, with feed back given to the new hire, on-site PI, Component Leader and Regulatory Consultant. After performance on the first 5 cases has been assessed, additional cases may be audited if necessary and additional training provided.

Corrections Process

*This section describes methods that will be used in correcting deficiencies identified in the QM process.*

Quality Control

Problems identified via the quality control process will be corrected by standard data query. The Coordinating Center will be ultimately responsible for the query process.

A standard query process is a useful tool in collecting accurate, consistent data. This tool can be utilized at any stages of the clinical research process. The query generation and resolution steps are as follows:

1. A team member may question a variable at any point of the data collection process.
2. For purposes of tracking, a log is maintained which includes:
   1. question
   2. the study or protocol name
   3. form or data collection instrument
   4. interval on study in which the data was collection
   5. patient identification number
   6. variable in question
   7. date of query generation
   8. date of query resolution
   9. disposition of query
3. The question is written in a clear manner and sent to the site clinical staff. The query should include:
   1. the study or protocol name
   2. form or data collection instrument
   3. interval on study in which the data was collection
   4. patient identification number
   5. variable in question
4. The site clinical staff responds to the query in writing. If necessary, data collection instruments and source documents are revised. At times, a memo to patient file may be indicated. The response is sent to the Coordinating Center or generator of the query
5. Queries may be discussed in regularly scheduled meetings/conference calls which include the Protocol Team, Component Leader, and Investigators.
6. Upon receipt of the query response, the query log is updated to indicate that the query is resolved and the disposition recorded.
7. If necessary, study databases are revised with any appropriate accompanying documentation.

Quality Assurance

All items identified as in need of correction by the quality assurance audit will be documented on the QA tool. The on-site Key QA personnel will be responsible for making sure corrections have been made and providing feedback of such to the Regulatory Consultant. This process may also generate queries via the standard query process described above. In addition, the on-site Key QA personnel will facilitate any additional training or inservicing required as a direct result of the quality assurance audit.

Regulatory

The regulatory checklist will identify any missing or incomplete regulatory documentation. The on-site Key QA staff will coordinate corrective measures and report completion of such to the Regulatory Consultant in a timely manner.

Results Reporting

*This section describes which QM activity results will be reported.*

Quarterly quality control reports, semi-annual quality assurance audit reports, and semi-annual regulatory audit reports will be made available to the Director, Component Leaders, Regulatory Consultant, on-site Investigators and Protocol Team. These reports will be inclusive of any required corrective actions and a timeline for completion.

Staff Training

*This section describes the responsibilities for training employees in the Quality Management Plan.*

The Regulatory Consultant will assure that the on-site Key QA personnel are trained in all aspects of their responsibility. The Regulatory Consultant will also work with the Component Leaders and on-site Key QA personnel to assure all on-site staff are trained in their role in the Internal QM Plan. The Regulatory Consultant will provide continued education to on-site staff members during site visits and to Coordinating Center staff members twice annually. Each training session will be documented with attendance recorded.

Data management staff will be trained in QC procedures by the Senior Data Manager. On-site Data Managers will be responsible for assuring appropriate QC procedures are followed by all on-site data management staff.

Plan Revisions

*This section describes procedures for annual review of the Quality Management Plan.*

The entire Quality Management Plan will be reviewed annually by the Regulatory Consultant, Investigators, and Data Management Team. Specifically, the QM plan will be reviewed for staff compliance, query rate improvements, audit finding improvements, and feasibility of implementation. Input will be solicited from on-site Key QA personnel and data management staff. Findings of the review and suggested revisions for the coming year will be presented to the Director and senior level management for approval.

Reviewer: Review Date:

Study:

|  | Present | Absent | Comments |
| --- | --- | --- | --- |
| Study protocol |  |  |  |
| Amendments to protocol |  |  |  |
| Informed Consent form (IRB approved version) |  |  |  |
| IRB approval documentation for all protocol versions |  |  |  |
| Annual progress reports and re-approvals |  |  |  |
| FDA 1572 Statement of Investigator |  |  |  |
| CVs for all investigators |  |  |  |
| Laboratory certification |  |  |  |
| Laboratory normal range values for all participating labs |  |  |  |
| Site monitoring sign-in log |  |  |  |
| Severe adverse event reports |  |  |  |
| All correspondence (except financial matters) |  |  |  |
| Investigator Drug Brochure |  |  |  |
| Signature list for Study Staff |  |  |  |
| Screening Log |  |  |  |

Comments: (Reference item # and provide comments regarding missing or incorrect item)

________________________________________________________________________________________________________________________________________________________________________________________________________________________________________________________________________________________________________________________________________________________________________

Signature of person completing Regulatory Audit________________________________

Corrective Action:

________________________________________________________________________________________________________________________________________________________________________________________________________________________________________________________________________________________________

Date of Correction:

Signature of person making correction:

Table of Activities

I. Quality Control

| Activity | **Personnel** | **Timetable** | **Documentation** |
| --- | --- | --- | --- |
| 100% Eligibility Verification | Clinical Coordinator | Patient Enrollment | CRF signature |
| 100% Review of files prior to patient visit to ensure appropriate documentation and testing requisitions | Data Staff | Files sent to reception prior to patient visit | Sign off |
| 100 % Data Editing | Home visitor or Data Management Staff at Research Site | Point of patient contact | Scannable sign-off |
| 100% Clinical Cross-check | Medical Officer at Research Site | Time of clinic visit | Scannable sign-off |
| 100% Clinical Coordinator Review | Clinical Coordinator at Research Site | Within 24 hours of patient contact | Scannable sign-off |
| 100% Data management review of key variables prior to scanning or transmission to Coordination Center | Data Management Staff – Research Site | Scanning, week of data collection | Scannable sign-off |
| 100% Data Management Review of all variables | Data Management Staff | Within 3 days of form receipt | Scannable sign-off |
| 100% Comparison of database to forms verified | Students/data clerk | Time of reading and verification |  |
| Frequency check for outliers and logic checks | Data Management Staff | Monthly | Reports |
| Categorization of queries | Data Management Staff | Monthly | Reports |
| Adverse Event Programs | Data Management | Weekly | Reports |
| 10% Longitudinal file audit per study | Medical Officer/Research Nurse | Quarterly | Audit Tool |
| Intensive review of first 5 patients enrolled in a new trial | Medical Officer/Research nurse | Weeks 4, 8 and 12 | Audit Tool |
| 10 % Database audit/comparison of database to CRF or source document (i.e., laboratory logs) | Data management | Semi-annually | Reports |
| 10% review of patient files for appropriate compilation of study forms and requisitions | Data management | Monthly |  |

**II. Quality Assurance**

| **Activity** | **Personnel** | **Timetable** | **Documentation** |
| --- | --- | --- | --- |
| Review of regulatory binders for IRB approvals, IRB amendments, correspondence, 1572, Investigator CVs, staff training | Off-site visitors | Quarterly | Checklist |

STUDY TERMINATION CHECKLIST

| TASKS | PROPOSED DEADLINE | DATE COMPLETED | COMPLETED BY |
| --- | --- | --- | --- |
| Subjects | | | |
| Summarize patients’ last laboratory results and complete a referral to appropriate clinic as necessary |  |  |  |
| Schedule close out visit for all subjects after last clinical data collected |  |  |  |
| Maintain contact with patients and notify them of treatment study assignment and study findings |  |  |  |
| Test Article | | | |
| Inventory all unused and used drugs and diluents and finalize/reconcile all drug accountability records |  |  |  |
| Dispose of or return any unused vials per sponsors instructions and ensure correct documentation |  |  |  |
| Data | | | |
| Confirm all CRFs completed and transferred to Coordinating Center |  |  |  |
| Confirm all data queries satisfactorily resolved |  |  |  |
| Ensure all CRF binders and source documents are complete and available |  |  |  |
| Destroy all unused CRFs |  |  |  |
| Confirm all study databases are clean |  |  |  |
| Regulatory | | | |
| Inventory and file all signed informed consent forms in master file |  |  |  |
| Prepare and submit Final Study Report to IRB and sponsor and notify IRB of study termination |  |  |  |
| **Study Files** | | | |
| Clean patient charts, ensure that all loose papers are bound and unnecessary papers are disposed of. |  |  |  |
| Inventory and box screening charts, patient charts, x-rays, and regulatory binders for shipment to the Coordinating Center |  |  |  |
| Study Supplies | | | |
| Destroy all study specific lab requisitions |  |  |  |
| Inventory and store study specific equipment and supplies |  |  |  |
| Biological Samples | | | |
| Final disposition of samples - JCRC |  |  |  |
| Final disposition of samples - Core Lab |  |  |  |
| Final disposition of samples - Wandegeya |  |  |  |

Quality Assurance Audit Tool

| ID #____________ Study___________ Review Period________to__________ | | | | |
| --- | --- | --- | --- | --- |
| **Reviewer:** | **Review Date:** | **YES** | NO | NA |
| Informed Consent | | | | |
| 1. Signed, dated, and time recorded in ink by patient or parent/ guardian. | |  |  |  |
| 1. Signed prior to any screening procedures | |  |  |  |
| 1. IRB approved version in effect at time of consent matches consent version. | |  |  |  |
| Eligibility | | | | |
| 1. Eligibility checklist compares to source documentation for each question. Eligibility verified. | |  |  |  |
| Laboratory Results | | | | |
| 1. For the period being audited, all lab tests as required by protocol are completed. | |  |  |  |
| 1. If there are any missing lab tests, is there a reason documented for the omission? | |  |  |  |
| 1. Refer to protocol for requirements for toxicity reporting. Are all toxicities as required by the reporting requirements of the protocol recorded in the source documents and the CRFs? If dosage changes required by protocol for toxicities, are they made as required? | |  |  |  |
| 1. Are appropriate patient identifiers and date of specimen collection present on hardcopy lab results? | |  |  |  |
| Concomitant Medications | | | | |
| 1. Refer to protocol being audited. Are any prohibited concomitant medications being taken by the patient? | |  |  |  |
| 1. Are concomitant medications recorded in source documents? | |  |  |  |
| 1. Are concomitant medications (as required by protocol) recorded in CRFs? | |  |  |  |
| Adverse Event Identification and Reporting | | | | |
| 1. Are there any missed AEs and SAEs? | |  |  |  |
| 13. Are AEs/SAEs reported correctly, timely, and graded? | |  |  |  |
| Clinical Endpoints | | | | |
| 1. If the patient has reached an endpoint, is it source documented, with CRFs completed properly? | |  |  |  |
| 1. Has the patient reached an endpoint which is unreported or reported incorrectly? | |  |  |  |
| Missed Visits | | | | |
| 1. Are all missed visits documented in source documents with attempts to follow up with patient documented? | |  |  |  |
| TB Treatment/Drug Susceptibility | | | | |
| 1. Are the patient’s drug susceptibility testing results found in the source document? | |  |  |  |
| 1. If the patient was found to be resistant to any standard TB medications, was an alternate treatment regimen recorded in the source document? | |  |  |  |
| TB Treatment Outcome | | | | |
| 1. Is the patient’s TB treatment outcome supported by the source documents? | |  |  |  |
| Treatment/Study Discontinuation | | | | |
| 1. If the patient was terminated from the protocol early, is there clear documentation of the reason and all relevant laboratory tests? | |  |  |  |

Comments: (Reference item # and provide comments regarding missing or incorrect item)

Signature of person completing QA Audit Tool

Corrective Action:

Date of correction:

Signature of person making correction:

**Informed Consent for Screening**

Investigators

Christopher Whalen, M.D., M.S.

Case Western Reserve University School of Medicine

Cleveland, Ohio, U.S.A.

Telephone: 001-216-368-4192

Diane Havlir, M.D.

University of California, San Francisco

San Francisco, California, U.S.A

Telephone: 001-415-476-4082, ext. 400

Roy D. Mugerwa, M.B.Ch.B.,

Makerere Medical School – Mulago Hospital and Complex

Uganda-CWRU Research Collaboration

Kampala, Uganda

Telephone: 256-41-540-718

Your Rights

This form gives you information about screening for a research study that will also be discussed with you. Once you have had all your questions answered about the screening, and if you agree to be screened for this study, you will be asked to sign this form. You will be given a copy of this form to keep.

It is your choice to decide if you want to be screened for this study. You can leave the screening at any time. If you do not join the screening, your medical care will not change. You will not lose any benefits that you would otherwise have. You can be screened for and join another research study later, if one is available. You may ask all the questions you want after reading and listening to an explanation of this form.

Before you decide whether or not you want to volunteer to be screened for the study, you will be told the purpose of screening for this study. You will be told how it may help you, any risks to you, and what is expected of you. This process is called informed consent.

Background and Purpose of the Study

Tuberculosis is a serious infection that can affect the lungs and other important parts of the body. TB

is a common disease and can be life-threatening when left untreated. People who are infected with HIV, the virus that causes slim or AIDS, are much more likely to develop TB if they happen to breathe in the germ. HIV and TB together are common in Uganda and affect both men and women.

Current medications used to treat TB are almost always able to kill the TB germs. The treatment of TB with TB medications alone in a person with HIV infection may not always work as well as in a person without HIV. TB is particularly bad for people with HIV, because even though the TB is treated, it may make the HIV infection worse.

This research study, sponsored by the Unites States (U.S.) National Institutes of Health (NIH)/NAID, will study the treatment of HIV infection with antiretroviral drugs for a shortened amount of time and given the same time as medicines to treat the disease tuberculosis (TB). This research study is being done because the doctors want to see if this kind of treatment can help people with both HIV and TB avoid getting sicker with HIV/AIDS . This research study is being done to also see if this kind of treatment of both anti-TB medicines and antiretroviral drugs for a shortened amount of time is safe for people who have both HIV and TB.

Purpose of Screening for this Study

Screening is done to identify patients that might meet the criteria for a research study. You will be screened to see if you have both HIV and TB. You will not be able to join this study if you are not infected with HIV, the virus that causes AIDS. If you do not have HIV infection, the doctors will refer you to the National TB Program or you maybe able to join another research study for the treatment of your TB disease. You will also not be able to join this study if you do not have TB disease in your lungs. If you do not have TB disease, you will be referred to an appropriate clinic for your health problem.

You may not be able to join if your blood tests show that you have health problems such as severe liver or kidney problems or other serious health problems. You may not join this study if you have more advanced HIV infection. In more advanced HIV infection there are less CD4 cells, which are blood cells in the body that help fight infections. The study doctors believe that TB disease makes HIV infection worse, more so in people with a greater number of CD4 cells. If you have less CD4 cells so that the doctors think this shortened study treatment of antiretroviral drugs for only 6 months may not benefit you, the doctors will refer you to clinics that are experienced in treating HIV/AIDS. The doctors will refer you to the National TB Program for the treatment of your TB disease.

If you are pregnant or breastfeeding, you will not be able to join this study. The doctors will refer you to the National TB Program for the treatment of your TB disease. The doctors will refer you to the Mulago AIDS clinic and The AIDS Support Organization of Uganda (TASO) for management of HIV disease.

Screening Procedures

Whether or not you decide to be screened for the study, you can be examined by a medical doctor and have a chest X‑ray and sputum tests to see if you have TB. You can receive regular medical treatment based on the results of all of these tests in an appropriate clinic.

If you agree to be screened, the doctors will do the following:

- Ask you questions about your past health, any problems or illnesses you have had and any medicines you are taking now or in the recent past..
- Perform a check up, called a physical examination. This will include weighing you, and measuring your heart rate, blood pressure, and breathing rate.
- Take an X-ray picture of your lungs to check on how the TB disease may be spread throughout your lungs. If you have had an X-ray of your chest done in the 2 weeks before you are asked to join this study, the doctors may not need to repeat this.
- Ask you to cough up sputum into three different cups. This sputum will be used to be sure that you are infected with TB, and that it is not a different germ making you sick.
- Take blood from your arm (about 4 tablespoons). This blood will be used to do the following:
  - check on your general health, including the health of your kidneys, liver, and blood
  - measure the number of CD4 cells which are blood cells that help fight infection, you have in your body. If the number of CD4 cells is below a certain number, it may mean you have more advanced HIV infection and you will not be able to join the study.
  - test to see if you are infected with HIV, the germ that causes AIDS. You will be tested for HIV even if you have been tested elsewhere before this. If your blood test shows that you are not infected with HIV you will not be able to join the study.
- If you are a woman, take some of your urine to check to see if you are pregnant. In some cases, your doctor may use your blood to perform this test. If you are pregnant you will not be able to join the study. You will be referred to an antenatal clinic that provides treatment to prevent mother- to- child transmission of HIV.

You will be told the results of all your tests, whether or not you decide to join the study. You will be able to take the results of your tests to another doctor for other care, if you are unable to join or decide not to join the study.

The results of all your tests will be kept confidential. Your blood will only be used for the tests required for screening for this study, unless you agree to their use for other studies. You will have a chance on this form to show if you will allow these samples and information about your health to be studied at a later time or not. After all tests have been completed on blood taken from you, and if you do not want to allow your blood to be studied in other TB or AIDS related research, any remaining blood will be destroyed.

You will be asked to return to the clinic in several days to see if you can be in the study, based on the results of the tests. If you qualify for the study, and still want to take part in this research, you will receive a second informed consent, which includes more detail about the study. If you do not qualify for the study, you will be told where you can go for further care.

HIV Testing and Counseling

As mentioned above, if you agree to be screened for the study, your blood will be sent to the laboratory for testing to see if you are infected by the HIV virus, the virus that causes AIDS. You will speak with a health care worker or counselor before and after your HIV test. The counselor will talk to you about HIV and how it is spread and how it is treated. You can ask the counselor any questions you have before your blood is tested. The counselor will give you the results of your test as soon as it is available. If you have questions about your test result, you may ask the study doctors or counselor questions about the HIV test at any time.

If your HIV test is positive, it means that you have been infected with the HIV virus. Whether or not you are able to join the study, or you decide not to, you will be given more information on how to avoid giving this virus to other people, where you can get healthcare for HIV infection, and how to protect your health as well as possible. If your HIV test is negative you will not be able to join the study. You will be counseled on how to avoid getting HIV infection.

The results of your tests will be kept confidential. Medical staff who are helping to take care of you will see your HIV test results. The sponsor of the study and other people who work on this study will also see your HIV test result, but not with your name attached to the result.

Risks

Blood drawing may cause some minor pain, bleeding or bruising where the needle enters the body, and, rarely, may cause fainting, felling of being lightheaded, infection, or the formation of a blood clot at the blood draw site.

Testing for and learning your HIV status may cause you anxiety.

If you take part in this research, you will have one or more x-rays of your chest to see if you have TB. These x-rays involve a small amount of radiation. Participating in this research gives you about the same amount of radiation as you would get from living in a high altitude city for 12 days, or taking 4 long airplane flights.

Benefits

You may have no direct benefit from agreeing to be screened this study. You may benefit by knowing your HIV test results. Taking part in screening for this study may help identify health problems you would not otherwise know about.

Alternative Treatments

Participating in screening for this study is voluntary. If you choose to not be screened for this study or if you decide to leave the study, you will receive the best standard TB drug treatment at the National TB Programme. If you are HIV infected you can receive care and ARV treatment that you would have to pay for at the Mildmay Clinic, the JCRC clinic, and Nsambya Hospital. You can also receive healthcare at the Mulago AIDS clinic and The AIDS Support Organization of Uganda (TASO) for management of HIV disease.

Confidentiality

Personal information will be collected from you but only the people working on the study will see it. No one else will know that you took part in screening for the study. A study number, which will only be known to you, the study personnel, and officials at the National Institutes of Health (NIH), will be used instead of your name. The code of numbers will be stored in a safe place. Only the study personnel and officials at the National Institutes of Health will have access to your medical records and personal identifiers. If you agree, other researchers may want to study information about your health and or your blood that is learned from this screening visit. They may be interested in studying these even if you are not able to enroll in the main study. These researchers would only be allowed to do so if they have approval from the JCRC Ethics Committee and the Ugandan National Council and Technology that shows what they will study and how they will protect your identity, and if you give permission for them to do so. You can say you do not want them to have access to your screening information or blood, and still be screened for this study.

In order to assure the project is in compliance with NIH regulation, U.S. auditors, NIH staff, the data safety monitoring board (DSMB), local Ministry of Health, or the Ethics Committee may look at or copy records that might show your name or study number.

Costs

There will be no cost to you for the screening clinic visits and screening laboratory tests.

Research Related Injuries

National Institutes of Allergy and Infectious Disease (NIAID) has no program for compensation for research-related injury. However, if you are injured as a result of taking part in screening, the study clinic will give you immediate treatment for your injuries. If needed, you will then be told where you may receive additional treatment for injuries. The cost of this treatment is free.

You are not giving up any of your legal rights by signing this informed consent document.

Questions or Problems

You should contact Dr. Roy Mugerwa at the clinic or by phone at 041-534-262 if you have any questions or concerns about the screening. You should contact Dr. Jesse Kagimba, the Chairman of the JRCR – IRB/REC, tel. 077431656 or the JCRC – IRB/REC office, 041 270 622 if you have any concerns about your rights as a research subject while you are undergoing the screening process.

Retention of Samples

With your permission, the doctors would like to store whatever is remaining of your blood sample after they use it for screening you for this study. They would not take extra blood from you. They would like to be able use these stored specimens for future studies. If you agree, they will store your sample with some information about you, such as your study number, initials, age, race, sex and information about your health problem. They could then do experiments on your samples and/or study aspects of your health noted in your medical chart in the future which might let them understand better why your body reacts the way it does to TB and to the medicine you will take.

Future researchers would only be able to study your samples, or your health information, if they obtain separate approval from the JCRC Ethics Committee, and the Ugandan National Council of Science and Technology. They would have to explain what they plan to study, how they plan to use your samples and/or information, and how your privacy will be protected before they could use any samples or data. They would not know your name, and would have access only to your screening number. Your name will not be released or associated with any of the information or samples studied now or in the future.

You can also agree to only let the doctors keep your samples if they take away all means of identifying your sample. They would not keep your study number, initials, age, race, sex and data about your health problem. The kinds of research the doctors can do with this kind of sample is more limited, but they still might be used. This means that you will not be able to change your mind in the future and tell us to discard your specimens, since we will not be able to identify which specimens belong to you.

You can say “no”, that you do not want these samples saved, or your health studied, in the future or you can say that the study doctors can only keep your samples and information if they take your study number and other identifiers off of them, and you can still be in this study.

Consent

I have been given a copy of this form and know that a copy of this form will be kept in my study file. I understand that I may leave the study at any time or that the study doctors may ask me to leave the study if it is felt to be in my best interests. I understand that by signing below or making my mark, I agree to join the study and follow the study procedures to the best of my ability.

I am giving my consent to have information collected for the purposes of screening for the PART study.

I am also marking whether I give my consent to store my personal information and blood for use in a future study. I understand that the researchers will request permission from appropriate authorities before they use my information or blood for research. I understand that I can say “no,” that I do not want these samples saved, or my health information studied in the future, and can still be in this study.

Subject’s initials should be placed on the line that indicates whether or not they give permission for samples to be retained.

_______ I DO give permission for blood and other samples and information about my health to be stored and used in future TB studies.

_______ I DO give permission for blood and other samples and information about my health to be stored and used in future TB studies only if all information about me that could be connected to the samples or health information is destroyed first.

_______ I DO NOT give permission for blood and other samples, or my health information, to be stored and used in future TB studies.

**Summary of your rights as a participant in a research study**

Your participation in this research study is voluntary. Refusing to participate will not alter your usual health care or involve any penalty or loss of benefits to which you are otherwise entitled. If you decide to join the study, you may withdraw at any time and for any reason without penalty or loss of benefits. If information generated from this study is published or presented, your identity will not be revealed. In the event new information becomes available that may affect the risks or benefits associated with this study or your willingness to participate in it, you will be notified so that you can decide whether or not to continue participating.

**Authorization to Use and Disclose your Information**

You authorize Professor Mugerwa and other investigators, Makerere University and University Hospitals of Cleveland, and researchers at Case Western Reserve University and their employees to use and disclose information concerning you and your identity, medical history and information collected during this study for the following purposes: to study if TB and HIV treatment work better when given separately or together, and to study your sputum and blood later as part of future TB and HIV research. Such information may also be disclosed or used by others involved in or overseeing the study including the UHC Institutional Review Board, the study sponsor and its agents, as well as U.S., European, your and other governmental, regulatory and accrediting agencies. Foreign laws governing privacy, use and disclosure of health information may provide less protection than the laws of your country. Once disclosed your information may be redisclosed by others who are not required to maintain the privacy of your information. You may withdraw authorization to collect additional information about you at any time by writing to the local Principal Investigator, but information already collected may be continue to be used and disclosed. This authorization has no expiration date.

Consent for Screening

For Adults (age greater than 18 years):

____________________ _____________________ ________________

Subject's Signature Subject's Name (printed) Date

For Children (Age 13 to 17 years):

____________________ _____________________ _________________

Parent or Guardian’s Signature Parent or Guardian’s Name (printed) Date

Assent (for Children Age 13 to 17):

____________________ _____________________ _________________

Child's Signature or Mark Child’ Name (printed) Date

(if capable)

Signature of Person Obtaining Consent Date / Time

## Printed Name and Title of Person Obtaining Consent

## (Must be study investigator or individual who has been designated in the Checklist to obtain consent.)

##

Signature of Witness Date / Time

(Witness signature is required **only** if oral translation is performed at time of consent for participant who does not read or write well. A witness signature is **not** required if participant can read or write.)

### ___________________________________________ Date_____________

Signature of Principal Investigator or designee

(Affirming subject eligibility for the Study and that informed consent has been obtained.)

**Study Consent**

**Study Consent**

Investigators

Christopher Whalen, M.D., M.S.

Case Western Reserve University School of Medicine

Cleveland, Ohio, U.S.A.

Telephone: 001-216-368-4192

Diane Havlir, M.D.

University of California, San Francisco

San Francisco, California, U.S.A

Telephone: 001-415-476-4082 ext. 400

Roy D. Mugerwa, M.B.Ch.B.,

Makerere Medical School – Mulago Hospital and Complex

Uganda-CWRU Research Collaboration

Kampala, Uganda

Telephone: 256-41-540-718

Your Rights:

This form gives you information about a research study that will also be discussed with you. Once you have had all your questions answered about the study, and if you agree to be in the study, you will be asked to sign this form. You will be given a copy of this form to keep.

It is your choice to decide if you want to be in this study. You can leave the study at any time. If you do not join the study, or if you leave the study, your anti-TB care will not change. You will not lose any benefits that you would otherwise have. You can join another research study later, if one is available. You may ask all the questions you want to after reading and listening to an explanation of this form.

Before you decide whether or not you want to volunteer to join the study, you will be told the purpose of the study. You will be told how it may help you, any risks to you, and what is expected of you. This process is called informed consent.

Background and Purpose

Tuberculosis is a serious infection that can affect the lungs and other important parts of the body. TB is a common disease and can be life-threatening when left untreated. People who are infected with HIV, the virus that causes slim or AIDS, are much more likely to develop TB if they happen to breathe in the germ that causes TB. HIV and TB together are common in Uganda and affect both men and women. Current medications used to treat TB are almost always able to kill the TB germs. The treatment of TB with TB medications alone in a person with HIV infection may not always work as well as in a person without HIV. TB is particularly bad for people with HIV, because even though the TB is treated, it may make the HIV infection worse.

Purpose of the Study

The purpose of this study, sponsored by the Unites States (U.S.) National Institutes of Health (NIH)/NAID, is to learn whether giving treatment both for TB and HIV at the same time is as good at curing TB and controlling HIV as treating TB first and then giving HIV treatment only when signs in the blood show that HIV infection is getting worse. To do this, the doctors will study people with TB and HIV. In this study, all patients will receive the usual treatment for TB. In this study, half of the patients will take a short course of antiretroviral (ARV) treatment for HIV for only 6 months, at the same time as the TB treatment. Half of the patients will wait until there are signs in the blood of severe HIV infection before starting the ARV treatment for HIV. HIV medications can sometimes cause serious side effects, especially when taking TB medication at the same time. So it may be healthier to wait than to take the ARV medication right away. The doctors want to learn whether to start the ARV medication earlier and treat for a short time or wait until more signs point toward the need for treatment. In this study, all patients will be given ARV treatment for HIV if, or when, the HIV disease becomes severe as found by blood tests. This research study is also being done to see if this way of treating TB and HIV is safe for people who have both infections.

You have been asked to take part in this study because the doctors have found that you have TB. You can join this study because you have both HIV and TB. About 350 people will take part in the study in Uganda. This study will last for about 5 years. All people on the study will be followed for at least 2 years after study enrollment. If you are enrolled early in the study, you will be followed until the last person enrolled finishes 2 years on study. Therefore, since the doctors think it will take about 3 years for all people to join, if you join the study and complete all the visits, you will be in this study for at least 2 years (24 months) and at the most 5 years (60 months).

Study Treatment

1. TB Treatment

All persons in this study will take these TB medicines for 6 months. The TB medicines that will be used are 4 standard drugs called isoniazid, rifampicin, pyrazinamide and ethambutol. You will take all these four drugs by mouth every day for 2 months. After 2 months, your pyrazinamide and ethambutol will be stopped and you will continue to take isoniazid and rifampicin by mouth every day for four additional months. This is the usual way TB is treated in Uganda and other places in the world.

2. Antiretroviral (ARV) Treatment Assignment

If you agree to be in the study, after you start the TB medications, your health will be followed weekly for up to 4 weeks to see if your health will allow you to be treated with ARVs. If your health permits, you may start ARVs two weeks after you start your TB medicines. If your health does not permit it at this point, for example, if you are found to be anemic, the doctors will check your health again one week later (Week 3). At that time, your health may have improved and you may be able to join the study. If not, the doctors will check you health again in another week (Week 4). Your health may improve to allow you to begin ARV treatment by week 4. If it is still not possible for you to start ARVs at week 3 or 4, you will stay on this study and you will receive your TB medicines and continue to be followed on the study. However, you will not start ARVs until your HIV disease is found to have gotten worse. You will be able to start ARV treatment later, if it is safe to do so.

If your health does allow you to start ARVs, the doctors will see if you will take them with your TB medicines or not. This will be decided by chance, like flipping a coin. One-half of the patients who are able to start ARVs will be treated with TB medications alone. The other half will be treated with TB medicines and a short-course of ARVs at the same time. If your health permits you to take ARVs, your chance of taking TB medicines and the short-course of ARVs is 1 in 2.

3. ARVs Used in this Study

The main antiretroviral drug used in this study is called Trizivir. Trizivir is medicine that contains three antiretroviral drugs in each tablet. These three medicines contained in Trizivir are called lamivudine, zidovudine and abacavir. Trizivir is approved for the treatment of HIV infection in both the U.S.A. and Uganda. Although Trizivir may not be the most effective HIV treatment for you we have chosen it because it is easy to use and may be taken with your tuberculosis medicine.

Another ARV drug that may be given to you if the doctor decides a bad reaction has occurred due to Trizivir is called Combivir, which is a medicine that contains lamivudine and zidovudine, two of the medicines that are also in Trizivir. Another ARV that might be used is called efavirenz, and the fourth one is called nevirapine. Combivir, efavirenz and nevirapine are also approved drugs for the treatment of HIV infection in both the U.S.A. and Uganda. The doctors may use any one of these ARVs depending on your health, the severity of your HIV infection, and how you react to your medicines.

4. ARV Treatment for All Subjects with Worsening HIV Disease

While you are on the study, your HIV infection may become worse. When your HIV disease worsens, your body has great difficulty fighting off infections. One way to see whether your disease is getting worse is to measure the number of a certain kind of blood cell in your blood. These cells are called CD4 cells, and when HIV disease gets worse, the number of CD4 cells in your blood goes down.

The doctors will measure your CD4 cells in your blood every 3 months after you are on the study, or more often if the doctors think it is necessary. They will do this to see if your HIV infection has become worse. They may also find your HIV disease is getting worse if you get seriously sick. If the study doctor’s find that your HIV infection has worsened, by finding that your CD4 cells are low or you have infections that are common to AIDS disease, you will be given antiretroviral drugs at that time, if it is safe to use them. If you are already receiving ARVs, the doctors may decide to change the medicine you are taking.

5. Treatment with ARVs After Study Completion

At the end of the study, you will be referred to clinics in Kampala that treat people who have HIV/AIDS with antiretroviral drugs. At that time, you will be responsible for buying your own antiretroviral drugs, if you wish to keep taking them. The antiretroviral drugs may be different than what was used during the study, depending on what is available at this time. The current cost of ARV treatment in Uganda is around $30 - 150 US per month, and may be more or less. The price of ARV drugs may not be the same at the end of the study as it is today.

Study Procedures

Whether you wait to take the ARV medicines or take the short-course of ARV drugs during TB treatment, you will be asked to come to the clinic for regular checks on your health and to be sure that your TB is cured.

You will take the TB medicines by mouth for 6 months. The doctors will need to be sure that you are taking all the medicines you are assigned to take. A health care provider or treatment supervisor will watch you swallow your TB medicines 6 days per week. This may be done in your home, at the clinic, at your workplace, or another convenient place arranged by you and your treatment supervisor. A health care provider or treatment supervisor will also watch you take one of two daily doses of ARV medication 6 days per week. You will be asked to take the second daily dose without supervision and return the pill packets to the treatment supervisor or home visitor.

You will continue to have check-ups on your health after you finish your TB treatment. You will be seen at the clinic every 3 months from 2-5 years, depending on whether you are one of the earliest or latest person to join the study. You can also come to the clinic if you feel sick, even if you are not scheduled for a visit.

There are three steps in this study. They are called Step 1, 2, and 3. These steps are explained below:

- Step 1 is the period of time from when you enter the study and are started on TB medications until the doctors determine whether your health will permit you to take ARVs with your TB medicines, if you should be randomized to receive those. This may last from 1 to 4 weeks after you consent to join this study. If the doctors find your health will allow you to take ARVs with your TB medicines, and if you are assigned to take them, you will enter Step 2. If your health will not allow it, you will take your TB medicines, and you will be followed according to the Step 2 follow-up schedule, which is explained below.
- Step 2 is the period of time from when you find out whether you will be taking ARVs with your TB medicine or not until the time the study is over or you get sicker with HIV, if you do at all. If you get sicker with HIV while you are on study and can safely take ARV treatment, you will enter Step 3.
- Step 3 is the period of time from when your HIV disease has worsened until the end of the study. You will enter Step 3 when your HIV disease has worsened and it is safe to start daily antiretroviral therapy. Once we know it is safe to treat you with the antiretroviral medicines, you will be started on them and followed closely by the study doctors and nurses. If you are already taking ARVs, the doctor may change your ARV medicine.

There are certain procedures and health checks that will be done at each step of the study. These are described below:

1. Step 1

1. Baseline Visit

At the beginning of the study, if you agree to join and take part in this research, you will:

- Have a physical exam, which includes taking your weight
- Have blood taken from your arm (2 tablespoons). This blood will be used to measure the number of HIV viral cells, and for storage for later use.
- Speak with the home visitor and decide where your DOT will be given.
- Take your first dose of TB drugs.
- Answer questions about how you have been feeling overall in your health and ability to do the activities you normally do (at baseline, Week 1, and months 12, 18, 24, 30, and 36)
- Be taken home by the home visitor and a driver.

b. Step 1 Follow-up Visits

You will be asked to come to the clinic each week while you are on Step 1. At these visits, the doctors will check your health. The doctors will ask if you are having any trouble with your TB medicines or if you have had any bad reactions to them. At Week 1, you will have a thorough physical exam and lab tests done to see if your health will allow you to start on the study antiretroviral therapy if you are assigned to that treatment. If by Week 2, you are eligible to start on the study intervention then you will enter Step 2 of the study, and start following a different study schedule. Otherwise, if the study doctors find that you are not ready at Week 2, you will be asked to return to the clinic at Week 3 and up to Week 4, to see if you will be able at these times to start on the study intervention. At all weekly these visits, you will:

- Have a physical exam at Weeks 1 and 2 (and Weeks 3 and 4 if needed)
- Answer questions about your health, and if you are having any new problems at Weeks 1 and 2 (and Weeks 3 and 4 if needed)
- Answer questions about your TB treatment at Weeks 1 and 2 (and Weeks 3 and 4 if needed)
- If you are a female of reproductive potential, have a urine pregnancy test done weekly up to Week 4. In some cases, the doctors may use blood to perform this test.
- Have blood taken from your arm (4 tablespoons) at Weeks 1 and 2 (and Weeks 3 and 4 if needed). This blood will be used to check your health, to see how your body is fighting the HIV and TB diseases, and also to study the HIV virus that is in your body.

2. Step 2

a. Step 2 – Before starting study intervention: Assessments at the Time of ARV Treatment Assignment

If your study doctors find that it is safe for you to take ARV medicines, you will:

- Have a physical exam.
- Answer questions about your health, and if you are having any new problems
- Have blood taken from your arm (2 tablespoons)
- Be assigned to either start a short-course of ARV therapy with your TB medication or continue on TB medication only.

If the study doctors find that you are not able to take the antiretroviral medicines, you will skip this visit, and be followed according to Step 2 – Follow-up visits schedule.

b. Step 2 – Study Intervention and Post-Intervention Follow-up Visits

During Step 2, you will be asked to come to the study clinic one and also two weeks after starting your treatment and then once a month during the first year, once every 3 months until you complete at least 2 years on this study. All persons enrolled in this study will come to follow-up visits until the last person enrolled completes 2 years on study, which could result in a total time on study of 5 years. If you are one of these people enrolled early in the study, you will be seen every 3 months during the second and third year, and then once every 4 months for remainder of the study. The health checks listed below will be done at the months listed, for as long as you are on study, but for at least 2 years.

At your Step 2 follow-up visits, the doctors will check your health. The doctors will ask if you are having any trouble with your medicines or if you have had any bad reactions to them. At each of these visits, you will:

- Have a physical exam (every visit).
- Answer questions about your health, and if you are having any new problems (every visit)
- Answer questions about your TB and ARV treatment (Week 1, Week 2, Month 1, 2, 3, 4, 5, 6)
- Give two sputum samples to see if the TB germ is still growing (Week 2, Month 1, 2, 5, 12, and 18 from starting on TB medication and if the doctors suspect you may have developed TB)
- Have a urine test to see if you are taking your TB medication (Week 2, Month 1, 2, 3, 4, 5, 6)
- If you are a female of reproductive potential, have a urine pregnancy test done (Week 2, Month 1, 2, 3, 4, 5, and 6, and whenever pregnancy is suspected). Some times the doctors may perform this test on your blood.
- Have blood taken from your arm for routine safety and liver tests (about 2 tablespoons) and to see if your HIV disease is getting worse, (Week 1, Week 2, Month 1, 2, 3, 6, 9, 12, 15, 18, 21, and 24 or longer to include 27, 30, 33, 36, and then every four months thereafter)
- Have a chest X-ray (Month 2, 6, 12 and 18 from starting TB medication and if the doctors suspect you may have developed TB).
- Have disease specific diagnostic tests as clinically indicated
- Answer questions about how you have been feeling overall in your health and ability to do the activities you normally do (at months 12, 18, 24, 30, and 36)

You may be asked to come to the clinic in between scheduled visits for more blood tests, another sputum sample, or other tests. You might be asked to come to these visits to check on your health or to follow-up on any tests you may have.

3. Step 3

a. Diagnosis of AIDS

While you are still in Step 2, the doctors will be checking your blood and health to see if your HIV disease worsens. If it does, then you will enter Step 3. In this part of the study, your clinic visits will be changed to follow your health more closely. You will come to the clinic when the doctors first find that you have AIDS and you will:

- Have a physical exam.
- Answer questions about your health, and if you are having any new problems
- Be started on ARV therapy, if it safe to do so, and continue ARV therapy until the end of the study. If it is not safe to start ARV therapy, then your doctor will examine you monthly, or as needed, until it is safe to start ARV therapy.
- Have a urine pregnancy test done
- Have 2 tablespoons (up to 30 ml) blood taken from your arm to see if it is safe to start ARV medication
- Have disease specific diagnostic tests as clinically indicated
- Answer questions about how you have been feeling overall in your health and ability to do the activities you normally do

b. Step 3 - Follow-up Visits following development of AIDS

If the study doctors find that you have developed AIDS, you will be asked to come to the clinic 2 weeks after starting your HIV medication, then every month for the first three months after you first developed AIDS and then every three months for up to 36 months or until the last patient enrolled on the study has been in the study for 2 years. At each of these visits, you will:

- Have a physical exam.
- Answer questions about your health, and if you are having any new problems
- Answer questions on your antiretroviral treatment
- Have a pregnancy test done (if the doctors think you may be pregnant)
- Have 2 tablespoons of blood taken from your arm (Month 1, 2, 3, 6, 12, 18, 24 and every 3 months for the next year and every 4 months thereafter) to see how your are responding to the medication
- Have disease specific diagnostic tests (as clinically indicated)
- Have a chest X-ray (if the doctors suspect you may have developed TB).
- Give two sputum samples to see if the TB germ is still growing (if the doctors suspect you may have developed TB)
- Answer questions about how you have been feeling overall in your health and ability to do the activities you normally do (Months 12, 18, 24, 30, and 36)

Any time that you come to a study visit at the clinic, you should tell the doctor if you are having any problems. You will need to tell the doctors if you have been admitted to a hospital or evaluated at a clinic since your last study visit. You will need to tell your doctors if you started any new medications or herbal remedies. If the doctors think that you might be getting sick from TB again, they may need to take some blood and sputum, and they may need to take an X-ray picture of your lungs to see if it is TB that is making you sick.

Sick Visits

If you feel sick or need to see a doctor, you may come to the clinic at any time. You do not need to wait until your regular appointment. If you are admitted to the hospital, please tell the research staff, or have someone in your family tell them if you are unable. If you need to leave the study early, inform the study doctors.

Risk of Standard TB Therapy

The medicines given for TB can cause side effects (bad reactions). These medicines are well known, and these side effects can be managed if you develop them. Side effects seen with these medicines can include: fever, rash, stomach upset, vomiting, loss of appetite, flushing (turning red), headache, joint aches, rashes, itching, tiredness, liver and urination problems. In rare instances, TB medications can cause abnormalities in the blood that causes increased risk of bleeding. Your medications may be changed if you have a problem with one or more of the drugs given in standard TB therapy.

Risks of Study Drugs

Although the medicines used in this study are used to treat HIV, these medicines may cause some unwanted effects. Although not all of these side effects may occur, if they do occur they may need medical attention. The drugs used in this study may have side effects (bad reactions), some of which are listed below. Please note that these lists do not include all the side effects seen with these drugs. These lists include the more serious or common side effects with a known, or possible relationship. The study doctors will give you more information for the drugs you will be given. If you have questions concerning the additional study drug side effects please ask the medical staff. You should let the doctors know if you are feeling any side effects or symptoms while taking study drugs. You should also let the doctors know if you become pregnant while on study. Your medications may need to be adjusted or stopped. Even if you have to stop a medicine because of side effects, you will not have to leave the study and you will be asked to return to clinic at the regularly scheduled visits.

Antiretroviral drugs, which may be given in this study, can cause changes in where body fat is located in the body of certain people. There also may be increased fat in areas where there was little or no body fat before. Why this happens and long term effects of these conditions are still not known at this time.

General Risks of Some of the HIV Drugs

Antiretroviral drugs used in this study include a class of drugs known as “nucleoside analogues”, or “nucleoside reverse transcriptase inhibitors” (NRTIs). The drugs used on this study, lamivudine, zidovudine and abacavir, and the combinations of these drugs in Trizivir® and Combivir®, belong to this group of medicines. These medicines can cause serious side effects and even death. These medicines can cause a build up of poisons in the blood (called lactic acidosis), an enlarged liver that may cause liver failure, and even death. The liver complications and death are more common in women taking these medicines. Some signs that you might have a build up of poisons in your blood include: unexplained weight loss, stomach discomfort, nausea, vomiting, fatigue, weakness, and shortness of breath.

1. Risks of Abacavir Sulfate (ZIAGEN®) *GlaxoSmithKline*

The following side effect has been associated with use of abacavir:

Some people may become allergic to abacavir. An allergic reaction may include many different symptoms, such as: fever, rash, feeling tired, upset stomach, vomiting, loose or watery stools, abdominal pain, cough, sore throat, shortness of breath, achiness, sores or rashes in the eyes or mouth, or a general feeling of illness.

These symptoms usually appear within the first six weeks after starting this drug but can occur at any time during treatment. This reaction can be severe and could even lead to death if abacavir is not stopped. The severe form of allergic reaction can also recur if abacavir is restarted after it has been stopped and can even lead to death. For this reason, if the study doctors find that you have had this kind of severe allergic reaction, they will ask you to return ALL of your abacavir pills to the clinic right away. Because the severe abacavir allergy could kill you, you will never again be able to take abacavir to treat your HIV infection. After your reaction to abacavir has gone away, the doctors will offer you treatment with efavirenz plus Combivir or nevirapine plus Combivir or another appropriate ARV regimen.

The frequency and clinical presentation of the allergic reaction to abacavir in children is similar to that seen in adults. In addition to those symptoms listed for adults, this syndrome may be seen as weakness or sluggishness, poor appetite, difficulty breathing, or a clinical picture similar to symptoms seen with infection of the blood.

**IF YOU THINK YOU MIGHT BE DEVELOPING A REACTION TO ABACAVIR, DO NOT TAKE ANY MORE DOSES AND CONTACT THE DOCTOR AT THE SITE IMMEDIATELY. YOU WILL ALSO BE GIVEN A CARD WITH THE CLINIC PHONE NUMBER AND INFORMATION TELLING YOU WHAT YOU SHOULD DO IF YOU THINK YOU ARE HAVING AN ALLERGIC REACTION.**

**NOTE**: Severe or fatal allergic-type reactions can occur within hours after abacavir is restarted in patients who have interrupted abacavir therapy. Allergic-type reactions to abacavir can occur in patients who have had no prior identified history or whose symptoms were previously unrecognized. If you interrupt abacavir for any reason, immediately contact the medical staff at the site. If your doctor decides to restart abacavir, you may need to be monitored more closely in the clinic or in the hospital.

Other side effects include:

- Upset stomach
- Vomiting
- Vague overall feeling of discomfort
- Feeling tired
- Decrease in appetite
- Loose or watery stools
- Abdominal pain
- Headache
- Serious skin rashes or irritations
- Increased triglycerides
- Inflammation or swelling of the pancreas with abdominal pain
- pain

Zidovudine (Retrovir®) *GlaxoSmithKline*

The following side effects have been associated with use of zidovudine:

- Decrease in the number of white blood cells that help fight infection
- Decrease in the number of red blood cells that may cause weakness, dizziness, and fatigue
- Muscle aches, weakness, and wasting
- Headache
- Upset stomach
- Vomiting
- Decrease in appetite
- Vague overall feeling of discomfort
- Lack of energy
- Feeling tired
- Sleeplessness
- Heartburn

Lamivudine (EPIVIR®) *GlaxoSmithKline*

The following side effects have been associated with the use of lamivudine:

- Decrease in the number of white blood cells that help fight infection
- Decrease in the number of red blood cells that may cause weakness, dizziness, and fatigue
- Muscle or joint aches, weakness
- Rash
- Nausea
- Vomiting
- Loose or watery stools
- Stomach cramps or pain
- Enlarged liver or liver irritation
- Heartburn
- Vague overall feeling of discomfort
- Lack of energy
- Feeling tired
- Sleeplessness
- Depression
- Increase level of acid in the blood (lactic acidosis)

3. Risks of Abacavir/Lamivudine/Zidovudine (as Trizivir®) and Lamivudine/Zidovudine (as Combivir®) Fixed-Dose Combination Tablets:

No new or unexpected side effects are observed with the abacavir 300-mg, lamivudine 150-mg, zidovudine 300-mg tablet (as Trizivir) or the lamivudine 150-mg/zidovudine 300-mg (as Combivir) than those observed when each drug is given separately. If you are a woman and become pregnant while receiving Trizivir, you may continue on this drug while you are pregnant.

4. Risks of Efavirenz (as SUSTIVA/ or STOCRIN) *DuPont Pharmaceuticals*

If you do not tolerate abacavir, you may be started on efavirenz. As with other medications, it has side effects. The effects on mental function include:

- Dizziness
- Trouble sleeping such as inability to sleep, abnormal dreams, and drowsiness
- Confusion
- Difficulty concentrating
- Hallucinations
- A feeling of strangeness and losing touch with reality
- An exaggerated feeling of well-being
- Agitation or anxiety

If alcohol or mind- or mood-altering drugs are used with efavirenz, it is possible that the above symptoms could become worse.

Serious psychiatric problems include:

- Depression, which may be severe
- Suicidal thoughts or attempts (rarely)
- Aggressive behavior
- Psychosis-like symptoms, such as abnormal thinking, paranoia, and delusions

People with a history of psychiatric problems may be at greater risk for these serious psychiatric problems.

Other risks include:

- Rash
- Upset stomach
- Loose or watery stools
- Headache

Increases in a substance in the blood (a type of a pancreatic enzyme) which can mean problems with the pancreas, such as inflammation or swelling of the pancreas with abdominal pain

- Increase in cholesterol
- Increase in triglycerides
- Abnormal liver function tests and inflammation of the liver (hepatitis)
- Abnormal vision
- Fever
- An abnormal or unusual distribution of body fat

Efavirenz may cause birth defects in people. You should not become pregnant while taking efavirenz as this may harm your unborn baby. Because this drug could harm an unborn baby, you will need to have a pregnancy test done on your blood or urine no more than 48 hours before you start taking efavirenz. If you become pregnant, notify the doctors with the study. If you are not pregnant, but you are able to become pregnant (that means that you are not surgically sterile or that you have not completed menopause at least 2 years ago, you will need to agree to use at least TWO forms of contraception, one of which must be a barrier method (condom, diaphragm, or cervical cap) and the other must be a reliable form of birth control such as hormonal contraception or spermicide. You will also have to agree to use these methods for the entire time you take efavirenz and for six weeks after you stop taking it. The clinic staff can assist you, if needed, in helping you obtain the forms of birth control needed if you are a woman who might become pregnant while on efavirenz.

5. Risks of Nevirapine (Viramune®) *Boehringer Ingelheim Pharmaceuticals, Inc*.

The following serious side effects have been associated with use of Nevirapine:

Severe liver damage that can cause death may occur. People with higher CD4 cell counts are at increased risk for developing liver damage, which is often associated with a rash. Women with CD4 cell counts greater than 250, including pregnant women receiving chronic nevirapine therapy, are at even higher risk for developing liver damage. For this reason, women with CD4 counts greater than 250 or men with CD4 counts greater than 400 wil not be able to take nevirapine as an alternate treatment. People who have abnormal liver function tests before starting nevirapine and people with active Hepatitis B or C infection are also at higher risk for liver damage.

If you are developing liver damage, you may have one or more of the following:

Tiredness

- General feeling of illness
- Loss of appetite
- Nausea
- Pale stools
- Dark urine
- Yellowing of the skin or whites of your eyes
- Liver tenderness or abnormal liver function tests

Rash is the most common side effect associated with Nevirapine. Rash occurs more often in women. Most rashes occur early during treatment. The rash may be severe and rarely may cause death. One of the risk factors for developing serious skin reactions includes failure to take nevirapine properly during the first 14 days of treatment.

Hypersensitivity reactions (HSR), which can rarely be fatal, may occur. Symptoms associated with an HSR include rash, fever, fatigue, muscle or joint aches, blisters, mouth sores, facial swelling, red eyes and irritation of the eyes, general feeling of discomfort, hepatitis, kidney problems, and/or changes in white blood cell levels.

The risk of people developing any of the serious side effects listed above is greatest during the first few months of treatment, but these side effects also can occur later. If you develop any of the side effects listed above, no matter how long you have been receiving nevirapine, you must contact your health care provider right away and try to be seen by the medical staff at your site before your next dose. If you and your doctor then decide to stop your treatment because of symptomatic hepatitis, hypersensitivity or severe skin reactions, you should never take Nevirapine again.

Other side effects include:

• Fever

• Headache

• Upset stomach

Risks of Taking both Standard TB therapy and Antiretroviral drugs

There is a risk of more side effects when TB and ARV medications are taken together. There may also be other risks, which are still unknown, as a result of taking these drugs in combination. Sometimes, people taking HIV medicines with TB medicines feel like they are getting sicker with their TB, and have symptoms like increased fever. You should let the doctor know if you are having these problems.

Risk of Drawing Blood

Blood drawing may cause some minor pain, bleeding or bruising where the needle enters the body, and, rarely, may cause fainting, felling of being lightheaded, infection, or the formation of a blood clot at the blood draw site.

Risks of Chest x-ray

If you take part in this research, you will have one or more x-rays of your chest to see if you have TB. These x-rays involve a small amount of radiation but will cause no harm to your body.

Other Information

There is a risk of serious and/or life-threatening side effects when non-study medications are taken with study drugs. For your safety, you must tell the study doctor or nurse about all medications or home remedies you are taking before you start the study and also before you take any treatment. You must also tell the study doctor or nurse before taking any non-study medications or home remedies while you are on the study. In addition, you must tell the study doctor or nurse before enrolling in any other clinical trials while on the study.

Pregnancy

The medicines used in this study may be unsafe for unborn babies. The risks to unborn babies for each drug are listed in the section called “Risks.” If you are having sex that could lead to pregnancy, you must agree not to become pregnant or make a woman pregnant.

Because of the risk involved, if you are on antiretroviral therapy then you and your partner must use a reliable method of birth control. The study staff will discuss the options with you. They can advise you as to what options are considered reliable, and where you can obtain these if you need to. If you are taking efavirenz, you must use TWO methods of birth control, one of which must be a barrier method. You must continue to use your birth control method regardless of which ARV you are taking until **6 weeks** after stopping . You may choose from the birth control methods listed below. If you are on TB medication that includes rifampicin **the choice of contraception must also include a barrier method**:

- Hormonal birth control drugs that prevent pregnancy given by pills, shots or placed under the skin
- Male or female condoms with or without a cream or gel that kills sperm
- Diaphragm or cervical cap with a cream or gel that kills sperm
- Intrauterine device (IUD)

If you are assigned to receive study drugs that require more or less strict birth control than what is described, the study staff will discuss your options.

If you are a woman who can become pregnant, you must have a pregnancy test before you enter this study. Women who are pregnant or breast-feeding their baby may not join the study. If you think you may be pregnant at any time during the study, tell your study staff right away. The study staff will talk to you about your choices. If you become pregnant during the study, you will remain part of the study, if you wish. You will be referred to an antenatal clinic for your obstetric care. If you are receiving Trizivir at the time that pregnancy is discovered, you may continue taking the medication for the duration of your pregnancy. If you are receiving efavirenz when pregnancy is discovered, you must stop it immediately as it is harmful to your baby. Study investigators may substitute it with nevirapine, if your blood test shows that it is safe to do so, or recommend another combination that is safe for you and your baby.

Benefits

Taking part in this study may help the researchers find out if people can take ARVs at the beginning of their HIV infection, and then not again until much later, and perhaps have fewer side effects and a better response to TB treatment. Your health will be followed more closely than usual while you are on the study, which may help you to feel better. You will get TB medicines as part of this study. Apart from the medicines, you may receive no benefit from this study. However, knowledge gained from this study may in the future help others infected with HIV.

New Findings

You will be told of any new information learned during the course of the study that might cause you to change your mind about staying in the study. At the end of the study, you will be told when study results may be available and how to learn about them.

Reasons For Withdrawal From The Study Without Your Consent

You may have to leave the study for any of the following reasons: if your doctors decide that staying in the study would be harmful to you, if you do not keep your follow‑up visits at the clinic, or if the study is canceled.

Alternative Treatments

Joining this study is voluntary. If you choose to not join the study or if you decide to leave the study, you can receive the best standard TB drug treatment at the National TB Programme. If you leave the study by your own decision, you may be able to obtain your own treatment with ARVs, but you may also find that there are no treatment alternatives available to you.

If you are HIV infected you can receive care and ARV treatment for a fee at the Mildmay Clinic, the JCRC clinic, and Nsambya Hospital. You can also receive healthcare at the Mulago AIDS clinic and The AIDS Support Organization of Uganda (TASO) for management of HIV disease.

Alternative treatments for HIV infection are protease inhibitor drugs, nonnucleoside reverse transcriptase inhibitor drugs, and nucleoside analogue reverse transcriptase inhibitor drugs. There are also other investigational treatments available, although all of these treatments may not be available in Uganda. Before you decide to take part, your study team will give you information about the benefits and risks of the treatments available in Uganda.

Confidentiality

Personal information will be collected from you but only the people working on the study will see it. No one else will know that you took part in the study. During the study, a number will be used on your medical records instead of your name. You will know this number as will the study personnel. Officials at the National Institutes of Health (NIH) in the United States will also know the number. Only the study personnel and officials at the National Institutes of Health will be able to link your medical records and personal study number.

This study must comply with the rules and regulations set down by the NIH because this is the sponsoring institution. As a result, experts who are asked to evaluate the progress of the study may look at or copy records that might show your name or study number. The experts are under strict instructions to keep all material from the study confidential. These experts may be from any of the following organizations: NIH/NIAID, Data Safety and Monitoring Board, Ethics Committees, or members of the local Ministry of Health.

No publications based on this research will contain your name.

Costs

There will be no cost to you for the study drugs, your clinic visits during the study and for examinations and laboratory tests required in this study. You will pay for any costs for hospital­ization or treatment for medical problems that are not related to the study.

Research Related Injuries

National Institutes of Allergy and Infectious Disease (NIAID) has no program for compensation for research-related injury. However, if you are injured as a result of taking part in this study, the study clinic will give you immediate treatment for your injuries. If needed, you will then be told where you may receive additional treatment for injuries. The cost of this treatment is free.

You are not giving up any of your legal rights by signing this informed consent document.

Questions or Problems

You should contact Dr. Roy Mugerwa at the clinic or by phone at 041-534-262 if you have any questions or concerns about this study once you join. You should contact Dr. Jesse Kagimba, the Chairman of the JRCR – IRB/REC, tel. 077431656 or the JCRC – IRB/REC office, 041 270 622 if you have any concerns about your rights as a research subject while on this study.

Retention of Samples

With your permission, the doctors would like to store two teaspoonfuls of blood on most occasions when blood is drawn during the study. They would use these specimens for future studies. If you agree, they will store your sample with some information about you, such as your study number, initials, age, race, sex and information about your health problem. They could then do experiments on your samples in the future which might let them understand better why your body reacts the way it does to TB and to the medicine you will take.

You can also agree to only let the doctors keep your samples if they take away all means of identifying your sample. They would not keep your study number, initials, age, race, sex and data about your health problem. The kinds of research the doctors can do with this kind of sample is more limited, but they still might be used. This means that you will not be able to change your mind in the future and tell us to discard your specimens, since we will not be able to identify which specimens belong to you.

You can say “no”, that you do not want these samples saved, or you can say that the study doctors can only keep your samples if they take your study number and other identifiers off of them, and you can still be in this study.

**Summary of your rights as a participant in a research study**

Your participation in this research study is voluntary. Refusing to participate will not alter your usual health care or involve any penalty or loss of benefits to which you are otherwise entitled. If you decide to join the study, you may withdraw at any time and for any reason without penalty or loss of benefits. If information generated from this study is published or presented, your identity will not be revealed. In the event new information becomes available that may affect the risks or benefits associated with this study or your willingness to participate in it, you will be notified so that you can decide whether or not to continue participating.

**Authorization to Use and Disclose your Information**

You authorize Professor Mugerwa and other investigators, Makerere University and University Hospitals of Cleveland, and researchers at Case Western Reserve University and their employees to use and disclose information concerning you and your identity, medical history and information collected during this study for the following purposes: to study if TB and HIV treatment work better when given separately or together, and to study your sputum and blood later as part of future TB and HIV research. Such information may also be disclosed or used by others involved in or overseeing the study including the UHC Institutional Review Board, the study sponsor and its agents, as well as U.S., European, your and other governmental, regulatory and accrediting agencies. Foreign laws governing privacy, use and disclosure of health information may provide less protection than the laws of your country. Once disclosed your information may be redisclosed by others who are not required to maintain the privacy of your information. You may withdraw authorization to collect additional information about you at any time by writing to the local Principal Investigator, but information already collected may be continue to be used and disclosed. This authorization has no expiration date.

Consent

I understand that by signing below or making my mark, I agree to join the study and follow the study procedures to the best of my ability.

I have been given a copy of this form and know that a copy of this form will be kept in my study file. I understand that I may leave the study at any time or that the study doctors may ask me to leave the study if it is felt to be in my best interests.

I also understand that the researchers are asking me if they can keep some of my blood and other samples for future research. I understand that I can say “no”, that I do not want these samples saved, and can still be in this study.

Subject’s initials should be placed on the line that indicates whether or not they give permission for samples to be retained.

_______ I DO give permission for blood and other samples to be stored and used in future TB studies.

_______ I DO give permission for blood and other samples to be stored and used in future TB studies only if all information about me that could be connected to the samples is destroyed first.

_______ I DO NOT give permission for blood and other samples to be stored and used in future TB studies.

Signatures:

For Adults (age 18 years or greater):

____________________ _____________________ ______________

Subject's Signature Subject's Name (printed) Date

For Children (Age 13 to 17 years):

____________________ _____________________ _____________

Parent or Guardian’s Signature Parent or Guardian’s Name Date

(printed)

Assent (for Children Age 13 to 17):

____________________ _____________________ ______________

Child's Signature or Mark Child’ Name (printed) Date

(if capable)

**Signature of Person Obtaining Consent Date / Time**

## Printed Name and Title of Person Obtaining Consent

## (Must be study investigator or individual who has been designated in the Checklist to obtain consent.)

##

**Signature of Witness** **Date / Time**

(Witness signature is required **only** if oral translation is performed at time of consent for participant who does not read or write well. A witness signature is **not** required if participant can read and write.)

### ___________________________________________ Date_____________

Signature of Principal Investigator or designee

(Affirming subject eligibility for the Study and that informed consent has been obtained.)

Viral Dynamics and Viral Diversity of HIV among Patients with HIV-TB Co-infection Receiving Anti-retroviral Treatment in Uganda

Investigators: Padmini Srikantiah, Joseph Wong, Diane Havlir, Edwin Charlebois, Roy D. Mugerwa, Alphonse Okwera, W. Henry Boom, Christopher C. Whalen

Background:

In the chronically HIV-infected patient, plasma HIV RNA levels represent the steady state of a dynamic equilibrium between production and clearance of HIV RNA. The initiation of antiretroviral treatment (ART) results in an exponential reduction of plasma HIV RNA during the first 7-10 days of therapy (1, 2). This first phase of decay reflects both the clearance of productively infected cells and the efficacy of an antiretroviral drug (3). Other factors that influence the rate of decay include the infectivity of the virus and the viral burst size of infected cells. Subsequent second-phase HIV RNA decay continues until a steady state of residual viremia is reached. The length of second-phase decay is estimated to range between 33 and 260 days, and the level of residual viremia is predicted by baseline HIV DNA level (4). The development of bacterial and viral co-infections in an HIV-infected person can result in states of heightened immune activation (5), which may lead to the generation of additional permissive host cells for virus replication and thus affect the rate of first and second phase decay.

Among persons with HIV in Africa, active tuberculosis (TB) represents the most commonly encountered opportunistic infection. HIV-infected persons who develop TB may have high pre-existing HIV viral loads (6), and the development of active TB in HIV-infected persons may lead to further increases in the plasma HIV-1 RNA load (7). Furthermore, TB/HIV co-infection is accompanied by marked systemic immune activation. The expansion of ART to countries in Africa where both TB and HIV are endemic provides an opportunity to study the potential effects of immune activation due to TB on HIV viral dynamics after initiation of ART.

The susceptibility of viral subtype variants to a particular ART regimen may also influence the dynamics of HIV plasma RNA decay. Although most published data on viral dynamics relate to HIV subtype B, the viral diversity of HIV-1 subtypes in Africa varies greatly from that found in the United States. In Uganda, the two principal subtypes of HIV-1 encountered are A and D (8). These subtypes may differ not only from subtype B, but may also vary from one another. Findings from a cohort study among HIV-1 positive individuals in Uganda suggested that, in comparison with subtype A, patients with subtype D infection had faster disease progression (9). Recent data from the Rakai district in Uganda also suggest that infection with subtype D virus is associated with lower rates of HIV transmission and may preferentially infect cells with CXCR4 co-receptor, potentially explaining the faster progression of disease (Rakai). Data from perinatal studies in Uganda also indicate that the development of resistance to nevirapine after single dose prophylaxis in pregnant women is higher in subtype D than subtype A virus (10). These studies suggest that subtype-specific characteristics between A and D may affect not only the dynamics of viral decay, but also the level to which viral suppression is achieved, the progression of immunosuppression, and the development of antiretroviral resistance. The presence of dual HIV infection, which has been associated with higher HIV RNA viral load and faster CD4+ lymphocyte decline (11, 12), may add another layer of complexity to these issues.

With the expansion of ART in Africa, the need for feasible and inexpensive methods of monitoring response to HIV treatment that can be used in resource-limited settings becomes more urgent. The use of dried blood spots (DBS) as an alternative method for blood collection has recently been shown to be effective for assessing CD4+ lymphocyte count and HIV RNA viral load (13, 14). Dried blood spots have also been successfully used to monitor the development of resistance to ART among women in South Africa treated with perinatal nevirapine prophylaxis (15).

The PART trial provides an ideal framework to study the differences in first and second phase viral decay over a spectrum of immune activation among patients co-infected with HIV and TB. Differences in the characteristics of viral decay can be compared between co-infected patients who are receiving punctuated ART to those who are randomized to the delayed therapy arm. Furthermore, it presents an opportunity to characterize the predominant HIV subtypes in this patient population and evaluate how HIV subtype may affect viral dynamics, viral suppression, and the development of anti-retroviral resistance in the co-infected patient. Finally, it presents an opportunity to evaluate DBS methods for assessing CD4+ lymphocyte counts, HIV RNA viral load and ART resistance in an already heavily monitored patient population.

Specific Aims:

1. Determine the predictors of phase 1 and phase 2 HIV viral decay and viral suppression among HIV-TB co-infected individuals who receive ART. When at least 20 patients with sufficient virology data have been accrued in each PART study arm, we will also compare the differences in viral decay characteristics among co-infected patients randomized to punctuated ART to those randomized to delayed ART. Potential predictors that will be evaluated are:
   1. Markers of immune activation at initiation of anti-TB therapy
   2. Markers of immune activation at initiation of ART
   3. HIV subtype
   4. HIV co-receptors
   5. Baseline HIV DNA and RNA
   6. Baseline ART resistance or polymorphism
   7. Baseline CD4+ lymphocyte count
   8. Presence of HIV dual infection
2. Understand the frequency and predictors of anti-retroviral resistance among HIV-TB co-infected individuals among study participants who are randomized to receive immediate ART. Factors that will be measured and evaluated as potential predictors are:
   1. Baseline HIV viral subtype
   2. Baseline HIV genotype
   3. Markers of immune activation at initiation of anti-TB therapy and initiation of ART
   4. Baseline HIV DNA and RNA
   5. Adherence to anti-retroviral regimen
3. Determine the predictors of HIV viral diversity among HIV-TB co-infected individuals. Patients will be assessed at baseline and six months for the presence of quasi-species and new recombinant forms. The influence of ART in TB will be examined as a potential predictor of for the development of viral diversity at six months.
4. Compare the results of CD4, HIV RNA viral load and anti-retroviral resistance testing from dried blood spot testing to those found using traditional venipuncture methods.

Patient population for virology sub-analyses:

Patients enrolled in the PART study at the TBRU in Kampala, Uganda will be included in these analyses. These patients are recruited from the National TB and Leprosy Program (NTLP) clinic in Kampala.

Analytic Design:

For this prospective nested sub-analysis of PART one additional blood draw will be scheduled to answer questions related to viral dynamics, viral subtypes, and anti-retroviral resistance. Additional virology and immunologic assays will be performed on banked serum and plasma specimens obtained during routine blood draws as outlined in the PART protocol.

In addition to the currently scheduled tests at baseline (HIV RNA and CD4 lymphocyte count), we will determine baseline HIV DNA, HIV genotype, HIV viral subtype, HIV co-receptor, presence of dual infection, and markers of immune activation (CD38+, DR+). We propose the addition of a plasma HIV RNA level at day 7 and day 14, and the addition of a CD4 lymphocyte count level at day 14 and day 28 (month 1) of the study intervention phase. At six months, testing to determine HIV subtype and presence of dual infection or recombinant variants, HIV DNA and RNA load, as well as antiretroviral resistance testing will be repeated in all patients. For patients randomized to receive immediate ART, repeat antiretroviral resistance testing will also be scheduled at the time of first positive VL specimen after discontinuation of ART at six months.

Viral decay after initiation and discontinuation of ARV will be analyzed assuming biphasic exponential decay characteristics. Comparisons of decay rates will be made using non-p

arametric methods (paired or unpaired Wilcoxon test). Clinical measurements of immune activation and viral RNA and DNA load, as well as baseline CD4+ lymphocyte count and markers of viral subtype and baseline resistance will be fitted in a multivariate regression model to determine independent predictors of first and second phase viral decay rates.

The presence of anti-retroviral resistance will be determined by standard sequencing methods. To determine independent predictors of resistance mutations, measurements of viral dynamics, including HIV RNA and DNA, and viral decay rates, as well as viral subtype will be analyzed in a multivariate model.

Multi-region hybridization assay techniques will be used to quantify the presence of HIV viral subtypes, recombinant strains, and dual infection. Virologic and immunologic characteristics at baseline will be assessed to identify independent predictors of viral diversity.

Ethical considerations:

One additional study visit with one additional blood draw will be scheduled to accommodate the stated analytic goals. Patients may experience momentary discomfort during this additional blood draw. Adverse medical events are not anticipated. However, providers will manage untoward events or injuries according to routine practice. The virology sub-analyses does not require the collection of any additional, unique data, and will be conducted using information that has already been approved to be collected. The PART study informed consent has been amended to request permission from patients for one additional scheduled blood draw and clinic visit.

REFERENCES:

1. Ho DD, Neumann AU, Perelson AS, Chen W, Leonard JM, Markowitz M. Rapid turnover of plasma virions and CD4 lymphocytes in HIV-1 infection. Nature 1995;373:123-6.

2. Wei X, Ghosh SK, Taylor ME, Johnson VA, Emini EA, Deutsch P, et al. Viral dynamics in human immunodeficiency virus type 1 infection. Nature 1995;373:117-22.

3. Perelson AS, Neumann AU, Markowitz M, Leonard JM, Ho DD. HIV-1 dynamics in vivo: virion clearance rate, infected cell life-span, and viral generation time. Science 1996;271:1582-6.

4. Havlir DV, Strain MC, Clerici M, Ignacio C, Trabattoni D, Ferrante P, et al. Productive infection maintains a dynamic steady state of residual viremia in human immunodeficiency virus type 1-infected persons treated with suppressive antiretroviral therapy for five years. J Virol 2003;77:11212-9.

5. Lawn SD, Butera ST, Folks TM. Contribution of immune activation to the pathogenesis and transmission of human immunodeficiency virus type 1 infection. Clin Microbiol Rev 2001;14:753-77, table of contents.

6. Day JH, Grant AD, Fielding KL, Morris L, Moloi V, Charalambous S, et al. Does tuberculosis increase HIV load? J Infect Dis 2004;190:1677-84.

7. Morris L, Martin DJ, Bredell H, Nyoka SN, Sacks L, Pendle S, et al. Human immunodeficiency virus-1 RNA levels and CD4 lymphocyte counts, during treatment for active tuberculosis, in South African patients. J Infect Dis 2003;187:1967-71.

8. Hu DJ, Baggs J, Downing RG, Pieniazek D, Dorn J, Fridlund C, et al. Predominance of HIV-1 subtype A and D infections in Uganda. Emerg Infect Dis 2000;6:609-15.

9. Kaleebu P, Ross A, Morgan D, Yirrell D, Oram J, Rutebemberwa A, et al. Relationship between HIV-1 Env subtypes A and D and disease progression in a rural Ugandan cohort. Aids 2001;15:293-9.

10. Eshleman SH, Guay LA, Mwatha A, Brown ER, Cunningham SP, Musoke P, et al. Characterization of nevirapine resistance mutations in women with subtype A vs. D HIV-1 6-8 weeks after single-dose nevirapine (HIVNET 012). J Acquir Immune Defic Syndr 2004;35:126-30.

11. Grobler J, Gray CM, Rademeyer C, Seoighe C, Ramjee G, Karim SA, et al. Incidence of HIV- 1 Dual Infection and Its Association with Increased Viral Load Set Point in a Cohort of HIV-1 Subtype C-Infected Female Sex Workers. J Infect Dis 2004;190:1355-9.

12. Gottlieb GS, Nickle DC, Jensen MA, Wong KG, Grobler J, Li F, et al. Dual HIV-1 infection associated with rapid disease progression. Lancet 2004;363:619-22.

13. Mwaba P, Cassol S, Nunn A, Pilon R, Chintu C, Janes M, et al. Whole blood versus plasma spots for measurement of HIV-1 viral load in HIV-infected African patients. Lancet 2003;362:2067-8.

14. Mwaba P, Cassol S, Pilon R, Chintu C, Janes M, Nunn A, et al. Use of dried whole blood spots to measure CD4+ lymphocyte counts in HIV-1-infected patients. Lancet 2003;362:1459-60.

15. Gordon M, Graham NM, Bland R, Rollins N, Claassen M, Coovadia HM, et al. Resistance patterns in mother-infat pairs following single dose nevirapine (NVP) for the revention of mother-to-child transmission (MTCT) of HIV-1. In: XV International AIDS Conference; 2004; Bangkok, Thailand; 2004.
