# Supplementary material for: Tuberculosis Treatment in HIV Infected Ugandans with CD4 Counts >350 Cells/mm3 Reduces Immune Activation with No Effect on HIV Load or CD4 Count
Source: PLoS One. 2010 Feb 22;5(2):e9138. doi: 10.1371/journal.pone.0009138 (PMC2825253; doi:10.1371/journal.pone.0009138)
Supplement: Figure S2 — IRB Approval from the Joint Clinical Research Center in Kampala, Uganda. (0.04 MB PDF) [file pone.0009138.s004.pdf]

Joint Clinical Research Centre

Vision A Vibrant Self Sustaining Centre of Excellent in Medical Research, Training and Healthcare Services

Plot 893 Ring Road Butikiro House- Mengo
P O Box 10005 Kampala Uganda
Fax 256-414- 342632
Email jcrc@jcrc co ug
Website http//www jcrc co ug

Telephone 256-414-270283
270622
273515
342521
Helpline 256-414-273062

IRB OFFICE

September 25, 2008

APPROVED
25 SEP 2008
JOINT CLINICAL RESEARCH CEN
IRB/ REC

Roy D Mugerwa, MBChB, M Med
Principal Investigator
Uganda – CWRU Research Collaboration
Kampala, Uganda

RE: STUDY RENEWAL APPROVAL

Your submission of the protocol **"A151219: Delaying HIV Disease Progression with Punctuated Antiretroviral Therapy in Patients with Tuberculosis in Uganda" version 3.0, 6 November 2006,** including the request for annual renewal, annual report and consent documents was reviewed by this IRB on September 25 2008 This to inform you that approval has been granted for study renewal, for a period of one year **with effect from October 18, 2008 to October 18, 2009.**

This is to also remind you to update and renew your study with the Uganda National Council for Science and Technology as well We wish you luck as you continue with this important study

Sincerely yours

Jesse Kagimba, M D , M Sc
Chairman JCRC-IRB/REC

c c Uganda National Council for Science and Technology
