# Supplementary material for: Tuberculosis Treatment in HIV Infected Ugandans with CD4 Counts >350 Cells/mm3 Reduces Immune Activation with No Effect on HIV Load or CD4 Count
Source: PLoS One. 2010 Feb 22;5(2):e9138. doi: 10.1371/journal.pone.0009138 (PMC2825253; doi:10.1371/journal.pone.0009138)
Supplement: Figure S3 — IRB approval from University Hospitals-Case Medical Center. (0.22 MB PDF) [file pone.0009138.s005.pdf]

UNIVERSITY HOSPITALS CASE MEDICAL CENTER

INSTITUTIONAL REVIEW BOARD FOR HUMAN INVESTIGATION

The University Hospitals Institutional Review Board has reviewed the proposal and informed consent

Submitted by: Whalen, Christopher and Mahan, Scott

Entitled: Delaying HIV Disease Progression with Punctuated Antiretroviral Therapy in Patients with Tuberculosis in Uganda

UH IRB Number: 11-03-04

Please be advised that with respect to: (1) The rights and welfare of the individuals
(2) The appropriateness of the methods to be used to secure informed consent
(3) The risks and potential medical benefits of the investigation

The Board Considers This Project:

☑ **FULLY ACCEPTABLE, without reservation; approved through** **11/10/2009**

☐ **NOT ACCEPTABLE for reasons noted:**

REMARKS: The continuing review is due by the date noted above.
IRB requires prompt reporting of the completion of a study.
Please reference the IRB number on future reviews and correspondence

Expedited Approval per 45 CFR 46.110(b)(2)

Changes to Protocol

Changes to Consent Form (English consent)

Changes to Consent Form (Lugandan consent)

HIPAA Authorization Revised (Privacy Board)

Pregnant Women/Fetuses under 45 CFR 46.204

Date of Committee Review:

Date of Approval: 3/3/2009

TYPE PROJECT ☐ New ☐ Renewal ☑ Addendum/Amendment

HUMAN RISK ☑ Yes ☐ No

SOURCE OF SUPPORT ☐ None ☐ Departmental ☑ Outside Funding

Agency: NIH Agency Study Number: AI51219

ARE ANY OF THE FOLLOWING INVOLVED? ☐ No ☑ Yes

☑ Minors ☐ Neonates ☐ Fetuses/Abortuses ☐ Prisoners ☑ Pregnant Women ☐ Mentally Retarded ☐ Mentally Disabled

Protocols involving children approved under

☐ 45 CFR 46.404 ☑ 45 CFR 46.405 ☐ 45 CFR 46.406*

*Both parents must give their permission unless one parent is deceased, unknown, incompetent, or not reasonably available, or when only one has legal responsibility for the care and custody of the child.

The UHCMC IRB operates under the HHS Federal Wide Assurance of Compliance number 00003937 and IRB registration numbers 00000684 and 00001691

Joseph Gibbons, MD, Chairman;
Barbara Daly, PhD, RN, Vice Chair; or Claudia Hoyen, MD, Vice Chair, or Paul Smith, DO, Vice Chair

EZ

Revised 09/01/2004
