# Supplementary material for: Tuberculosis Treatment in HIV Infected Ugandans with CD4 Counts >350 Cells/mm3 Reduces Immune Activation with No Effect on HIV Load or CD4 Count
Source: PLoS One. 2010 Feb 22;5(2):e9138. doi: 10.1371/journal.pone.0009138 (PMC2825253; doi:10.1371/journal.pone.0009138)
Supplement: Figure S4 — Enrollment Flow diagram. (0.03 MB DOC) [file pone.0009138.s006.doc]

Flow of participants through each stage of the larger Phase 3 clinical trial and clarification of those analyzed for the current sub-study.

Assessed for eligibility (n=4119)

Excluded (n= 3866)

Not meeting inclusion criteria

(n= 3857)

Refused to participate

(n= 4)

Other reasons

(n= 5)

**Allocation**

**Analysis**

**Follow-Up**

**253 Enrolled**

Analyzed (n=38)

Excluded from analysis (n= 72)

Reason for exclusion: missing flow analysis timepoints (n=72)

Withdrawn (n=7)

Give reasons

Death (n=3)

Lost to follow-up (n=2)

Withdrew consent (n=1)

Not eligible (lab error) (n=1)

Discontinued intervention (n= 0)

# Give reasons N/A

Allocated to TB Treatment Only

(n= 117)

Received allocated intervention

(n=117)

Did not receive allocated intervention

(n= 0 )

Give reasons N/A

Withdrawn (n= 15)

Give reasons

Death (n=5)

Lost to follow-up (n=5)

Withdrew consent (n=3)

Not eligible (lab error) (n=1)

MDR TB (n=1)

Discontinued intervention (n= 0)

Allocated to HIV and TB Treatment

(n= 115)

Received allocated intervention

(n=115)

Did not receive allocated intervention

(n= 0)

Give reasons N/A

No immune analyses yet performed on this arm.

232 Randomized
