# Supplementary material for: Tuberculosis Treatment in HIV Infected Ugandans with CD4 Counts >350 Cells/mm3 Reduces Immune Activation with No Effect on HIV Load or CD4 Count
Source: PLoS One. 2010 Feb 22;5(2):e9138. doi: 10.1371/journal.pone.0009138 (PMC2825253; doi:10.1371/journal.pone.0009138)
Supplement: Figure S1 — IRB approval from the Ugandan Council of Science and Technology. (0.03 MB PDF) [file pone.0009138.s003.pdf]

# Uganda National Council For Science and Technology

*(Established by Act of Parliament of the Republic of Uganda)*

UNCST

***Your Ref:***..........................

***Our Ref:***....................MV 838

***Date:***.............................
23/03/09

Prof. Roy Mugerwa
Principal Investigator
Uganda-CASE Research Collaboration
P.O Box 663
Kampala

Dear Prof. Mugerwa,

The Uganda National Council for Science and Technology (UNCST) has granted your request for approval to continue with the study entitled, **"Delaying HIV disease progression with punctuated antiretroviral therapy in patients with tuberculosis in Uganda, Version 3.0, dated 6 November 2006"** The approval will expire on March 26, 2010. If, however, it is necessary to continue with the research beyond this expiry date, a request for continuation should be made to the Executive Secretary, UNCST.

The Resident District Commissioner of Kampala District in which the study will be conducted is informed by copy of this letter, and is kindly requested to give you the necessary assistance to accomplish the study.

Yours sincerely,

Leah Nawegulo
for: Executive Secretary
UGANDA NATIONAL COUNCIL FOR SCIENCE AND TECHNOLOGY

***LOCATION/CORRESPONDENCE***

***Plot 3/5/7, Nasser Road***
[illegible]

***COMMUNICATION***

**TEL: (256) 414-250499, (256) 414 705500**
**FAX: (256) 414-234579**
